# Spatiotemporal Spike Coding of Behavioral Adaptation in the Dorsal Anterior Cingulate Cortex

**DOI:** 10.1371/journal.pbio.1002222

**Published:** 2015-08-12

**Authors:** Laureline Logiaco, René Quilodran, Emmanuel Procyk, Angelo Arleo

**Affiliations:** 1 INSERM, U968, Paris, France; 2 Sorbonne Universités, UPMC Univ Paris 06, UMR_S 968, Institut de la Vision, Paris, France; 3 CNRS, UMR_7210, Paris, France; 4 Escuela de Medicina, Departamento de Pre-clínicas, Universidad de Valparaíso, Hontaneda, Valparaíso, Chile; 5 Stem Cell and Brain Research Institute, Institut National de la Santé et de la Recherche Médicale U846, 69500 Bron, France; 6 Université de Lyon, Université Lyon 1, Lyon, France; University of Oxford, UNITED KINGDOM

## Abstract

The frontal cortex controls behavioral adaptation in environments governed by complex rules. Many studies have established the relevance of firing rate modulation after informative events signaling whether and how to update the behavioral policy. However, whether the spatiotemporal features of these neuronal activities contribute to encoding imminent behavioral updates remains unclear. We investigated this issue in the dorsal anterior cingulate cortex (dACC) of monkeys while they adapted their behavior based on their memory of feedback from past choices. We analyzed spike trains of both single units and pairs of simultaneously recorded neurons using an algorithm that emulates different biologically plausible decoding circuits. This method permits the assessment of the performance of both spike-count and spike-timing sensitive decoders. In response to the feedback, single neurons emitted stereotypical spike trains whose temporal structure identified informative events with higher accuracy than mere spike count. The optimal decoding time scale was in the range of 70–200 ms, which is significantly shorter than the memory time scale required by the behavioral task. Importantly, the temporal spiking patterns of single units were predictive of the monkeys’ behavioral response time. Furthermore, some features of these spiking patterns often varied between jointly recorded neurons. All together, our results suggest that dACC drives behavioral adaptation through complex spatiotemporal spike coding. They also indicate that downstream networks, which decode dACC feedback signals, are unlikely to act as mere neural integrators.

## Abstract 2

The decoding of spike trains from the dorsal Anterior Cingulate Cortex reveals that spike timing is relevant for cognitive information transmission and behavioral prediction, and it suggests that downstream areas are unlikely to act as mere neural integrators.

## Author Summary

In classical views of how information is processed in the brain, cognitive areas are often thought to encode incoming signals by a simple summation of spikes—action potentials fired by neurons and transmitted along the nerves—elicited by different cues. It is through this summation of spikes that cognitive areas are hypothesized to combine information from different cues and build memories. We investigated whether summation is relevant during the processing of signals emitted by the dorsal anterior cingulate cortex, a brain area which is thought to control behavioral adaptation in response to feedback cues that indicate the animal’s performance during a task. We found that a mere summation of spikes emitted in response to feedback cues actually extracts significantly less information compared to a code that also takes into account the timing of the spikes. Furthermore, we discovered that this temporal structure of the spike discharges predicts well the future behavior of monkeys. Overall, our findings suggest that the brain areas processing the signals emitted by the dorsal anterior cingulate cortex are sensitive to spike times and, thus, are unlikely to implement a mere approximate summation of inputs.

## Introduction

Behavioral adaptation is the process by which animals extract the rules of their environment and learn to respond to cues to increase their chances of survival. The frontal areas of the brain, including the dorsal anterior cingulate cortex (dACC), are involved in driving this process, although the underlying neuronal mechanisms are not well understood [1]. Most studies have focused on the number of spikes discharged by single dACC units after informative events occur. Other potentially informative features of the neural response, such as reproducibility of spike timing across trials, have typically been ignored. The reason for that may be the apparent unreliability of spike timing when observing frontal activity, which is consistent with theoretical studies [2]. Accordingly, most models of cognitive processing [3–5] rely on stepwise firing rate inputs, therefore disregarding the potential impact of the temporal structure of the driving signals. In the specific case of dACC, a recent theory [1] suggests that this area transmits a graded signal: the expected value of engaging cognitive resources to adapt the behavior. This signal has to be remembered from the moment when the current behavioral policy appears to be improper until the moment when a more appropriate strategy can be implemented. Hence, a simple neural integrator [6–9], which by construction is insensitive to spike timing, would be well suited to decode and memorize this signal. This neural integrator could be implemented by the lateral prefrontal cortex [10], which is a plausible dACC target during behavioral adaptation [11].

Nevertheless, some brain regions are known to be sensitive to both the timing [12] and the spatial distribution [13] of spikes within their inputs. These features may improve information transfer between neurons through, for instance, coincidence detection [14]. Some studies reported the presence of a temporal structure in frontal activity, including in dACC [15–23]. However, these observations are not sufficient to make conclusions about the relevance of this temporal structure for the downstream network’s dynamics and for the decision about future behavior. Indeed, to the best of our knowledge, there exists no study comparing the reliability and correlation with behavior of spike count and spike timing in individual frontal neurons during a cognitive task. Comparing spike count versus spike timing sensitive decoders is central to the general view of temporal coding [24]. In this framework, temporal coding can be defined as the improvement of information transmission based on sensitivity to spike timing within an encoding time window [24]. In fact, the temporal structure can be present but still not improve decoding, because spike timing and spike count can carry redundant information [25,26]. Furthermore, the temporal structure can be informative but still fail to correlate with behavior, suggesting that downstream processes disregard it and rely solely on neural integration [27,28].

**Fig 1:**
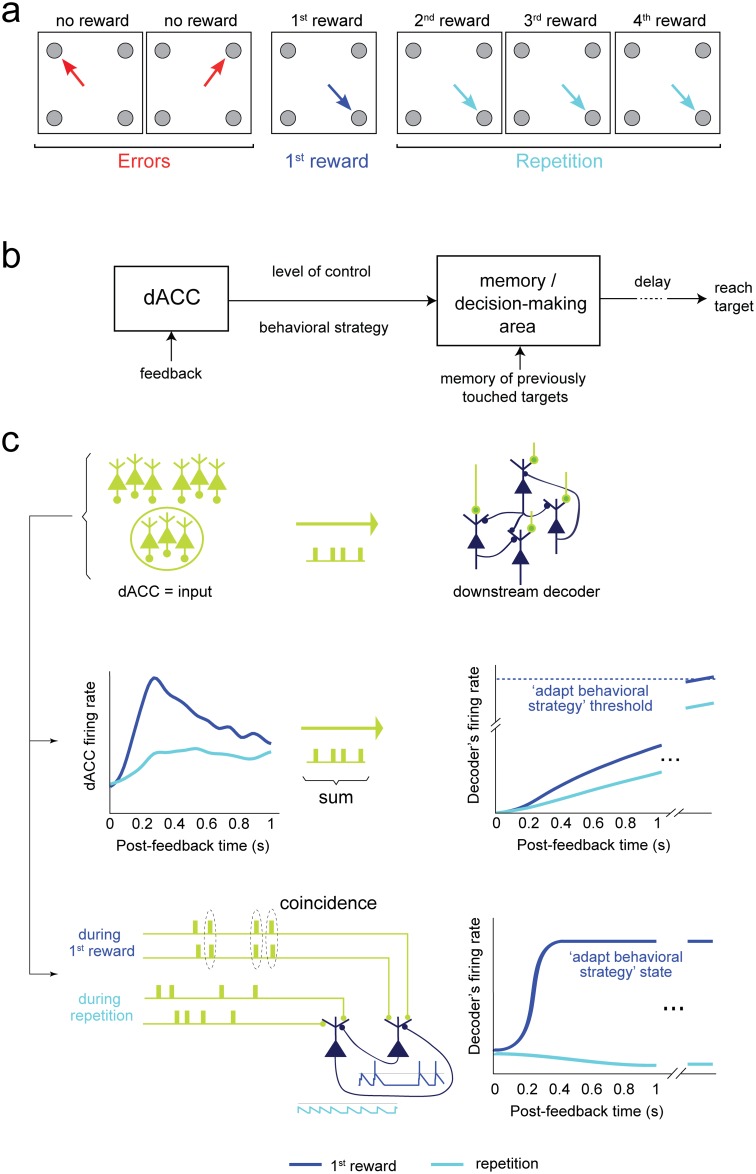
Task and proposed neural mechanisms. **(a)** During exploration, monkeys had to find, by trial-and-error, which of four targets resulted in a reward. After receiving the first reward, monkeys entered a repetition period and received additional rewards by touching the same target. **(b)** Plausible dACC role in the task [1,29–31]: it processes feedback information (error or reward) to signal a behavioral strategy (either exploration, switch toward repetition, or repetitive behavior). It would also signal the adaptive value of updating the behavioral strategy (“level of control”). A downstream area would combine dACC signals with a memory of previous choices to decide which target to choose next. **(c)** Spike count versus timing sensitive decoding of dACC signals. *Middle*: a neural integrator decoder [7,9,10] responding with a firing rate proportional to the sum of input dACC spikes. The decoder maintains a memory of past inputs and can store a continuum of level of control values. dACC neurons firing preferentially during either errors, first rewards, or both [29] could project to different neural integrators. *Bottom*: an example of spatiotemporal decoder that is sensitive to the temporal structure of dACC spike trains and implements a memory. The connections between neurons create two stable states, with high and low firing. The high-activity state sustained through recurrent connections signals the need to adapt behavior. This decoder would be sensitive to its input temporal structure, with some patterns favoring the transition to, and/or stability of, the high-activity state [32]. This scheme illustrates how temporal coincidences in the input could favor the discharge of downstream neurons.

Here, we address the issue of temporal coding in dACC. We use recordings from monkeys engaged in a trial-and-error learning task [29], in which performance relied on reward-based decision making and behavioral adaptation (Fig 1a and Materials and Methods). The task consisted of finding by trial and error which one of four targets was rewarded. Each trial led to the touch of a target and feedback: a reward if the touch was correct, nothing otherwise. In each block of trials (i.e., a problem), monkeys first explored the different targets in successive trials. The first reward indicated discovery of the correct response. This was followed a period in which they could repeatedly touch the correct target in three successive trials to exploit and receive additional rewards. The firing rate of single dACC units was previously shown to increase at feedback time during either exploration, repetition, or when switching between those two states [29]. Hence, dACC neurons may signal whether and/or how behavior should be adapted.

In this context, we probe the putative structure and function of a downstream neuronal network decoding dACC feedback-driven signals. To do so, we investigate to what extent the temporal structure of dACC spike trains, during post-feedback firing, could improve information transmission and predict behavior (Fig 1b). Assuming a neural integrator decoding scheme, the downstream network would compute and maintain the memory of the need for behavioral adaptation on the basis of the number of spikes emitted by dACC (Fig 1c, **middle**). Alternatively, the downstream network could be sensitive to the spatiotemporal structure of dACC activity (Fig 1c, **bottom**). For instance, temporal coincidences in the afferent dACC signals could favor the switch to, and maintenance of, a high-activity state in the downstream network to encode behavioral adaptation ([32], see also [33,34]).

We bring forth evidence for a spatiotemporal decoding of dACC activity. First, we find that there are informative temporal patterns in single units leading to a decoding more efficient than with spike integration. The optimal decoding time scale is in the range of 70–200 ms. Second, some spike coincidences across jointly recorded neurons are advantageous for decoding. Furthermore, the data suggest that downstream neurons could benefit from a non-linear *spatio*temporal integration of inputs. Finally, we develop a new method to evaluate to what extent dACC temporal patterns can predict the behavior of monkeys comparatively to spike count. Importantly, we find that temporal patterns are significantly and sizably predictive of the upcoming response time of monkeys.

## Results

To investigate temporal coding in dACC, we analyzed the activity of 145 and 189 individual neurons from monkey M and P, respectively.

### Optimal Temporal Sensitivity Improves Decoding of Single Units’ Behavioral Adaptation Signals

We first tested how single-trial, single-unit dACC activity could send signals that could drive behavioral adaptation after feedback. Behavioral adaptation occurred either after the first reward (thus switching from exploration to repetition) or after any error during exploration (Fig 1a). Signaling the need for adaptation requires that spike trains emitted during either first reward or errors can be discriminated from those emitted during repetitions (referred to as first reward and error discrimination analyses, respectively).

**Fig 2:**
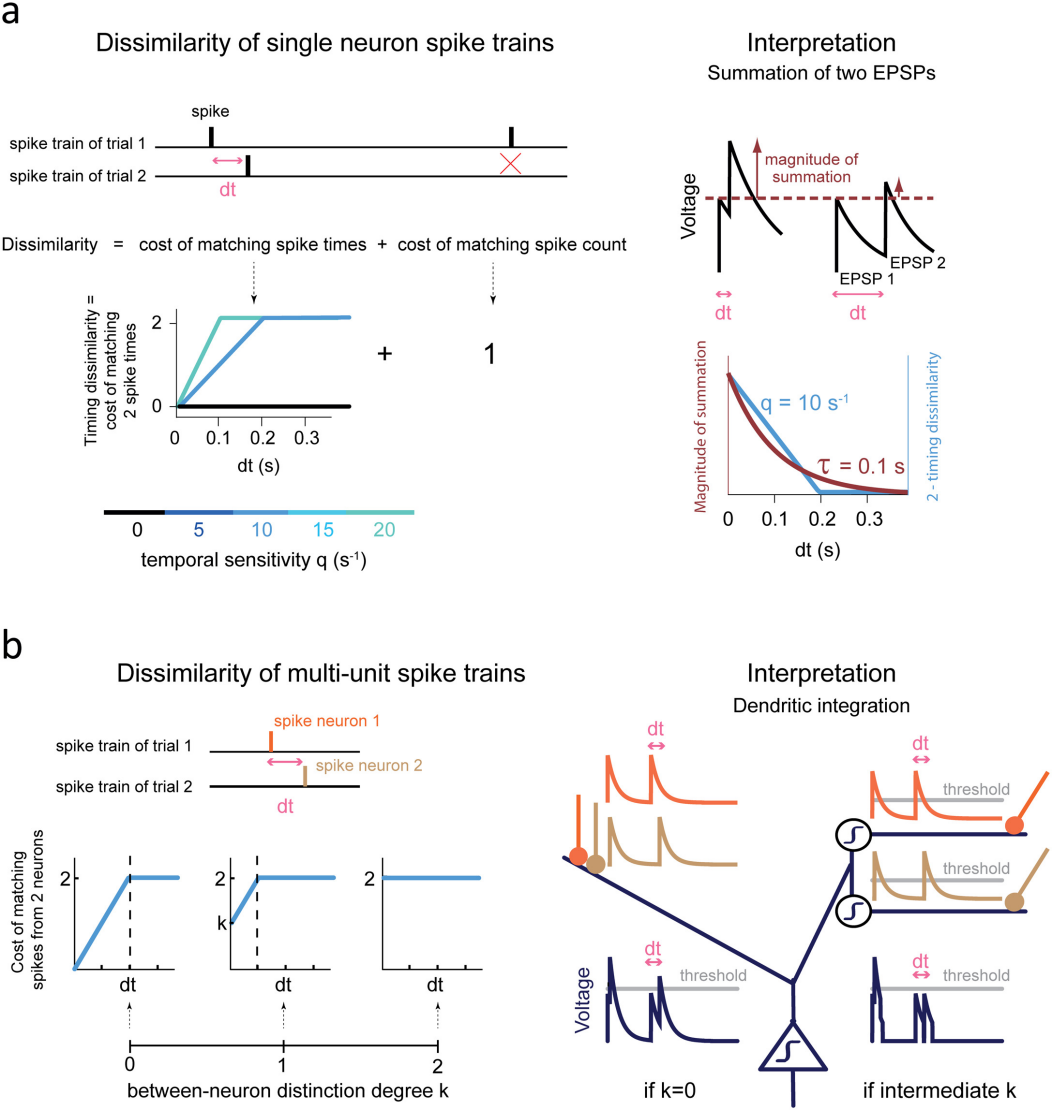
Decoding method. **(a)** Dissimilarity of single neuron spike trains. Left: the dissimilarity is the sum of the costs of matching spike times and cancelling the spike count difference [35]. The cost of matching spike times depends on the parameter q (temporal sensitivity). When q = 0 s^-1^ (black curve), the dissimilarity only reflects the difference in spike count. For q > 0 s^-1^, the dissimilarity increases as q times the interspike interval dt before saturating at 2. Right: Each value of q > 0 can be related to a given time scale of Excitatory Post Synaptic Potentials (EPSPs, here taken as simple exponential traces: up). Indeed, decoding with this q value and decoding by summation of these EPSPs both lead to a similar sensitivity to spike timing. For instance, q = 10 s^-1^ corresponds to a 0–200 ms range of dt for which the dissimilarities are smaller than 2 (the maximum). This can be matched to the range of dt with efficient summation of 2 EPSPs decaying with 100 ms time scale (S1 Text, section **4**). The 0–200 ms range of dt therefore gives rise to “temporal coincidences.” **(b)** Dissimilarity of multi-unit spike trains. *Left*: computation of the dissimilarity between two spike trains, each of which contains spikes from 2 neurons [13]. The dissimilarity depends on the parameter k, which determines the degree of distinction between the 2 neurons. The cost of matching 2 spikes is increased of a term k if the 2 spikes were emitted by 2 different neurons. As k increases, the matching of spikes emitted by the same neuron is favored. For higher values of k, there is a smaller range of between-neuron interspike intervals leading to dissimilarities smaller than 2 (i.e., leading to a temporal coincidence). *Right*: higher values of k can, for instance, be related to larger non-linearities in dendrites (here taken as thresholds and symbolized by a step within a circle). In the left dendrite, there are no non-linearities: synapses are close and depolarizations due to synaptic inputs can be directly summed and trigger firing (by crossing the threshold of the soma twice). This mirrors a maximal between-neuron summation, i.e., k = 0. Conversely, in the right dendrite the two synapses are on different sub-branches which both possess a threshold non-linearity. These thresholds (below which the synaptic currents are not transmitted to the soma) can prevent effective summation for large interspike intervals (second spike pair). This mirrors decoding with intermediate k values, causing only smaller interspike intervals to be associated with small dissimilarities between neurons (i.e. temporal coincidences).

Neurons in dACC could show early post-feedback responses specific to behavioral adaptation [29]. Therefore, we analyzed spike trains starting at the onset of the feedback delivered 600 ms after target touch. We will refer to any post-feedback time interval (i.e., following either an error, first reward, or repetition) as a “task epoch.” We quantified to what extent spike trains emitted during different task epochs were discriminable by a downstream decoder by classifying them based on a spike train dissimilarity measure [35]. This dissimilarity measure computed the minimal cost to transform the first spike train into the second one through two possible recursive operations: (i) adding or removing a spike, for a cost of 1; and (ii) changing the timing of a spike by dt, for a cost of *q dt ≤ 2*. Note that the maximum cost allowing two spikes to be temporally matched (coincidence detection) is 2 because it corresponds to the cost of removing and adding one spike (Fig 2a and Materials and Methods). This measure allows different temporal sensitivities of a downstream decoder to be evaluated by varying the parameter q. A value of q = 0 s^-1^ describes a decoder sensitive to pure spike count. On the other hand, a larger q value corresponds to a decoder sensitive to precise spike times. The larger the q value, the smaller the maximum interspike interval leading to coincidence detection, and the more the decoder disregards spike count. We stress that even when the neural activity is temporally structured, sensitivity to spike timing does not necessarily improve decoding. For instance, spike timing and spike count might provide redundant information, and then a neural integrator could be more robust (S1 Text, Sec. 4).

We quantified the classification performance (i.e., how well, on average, a spike train was correctly associated to the task epoch with the most similar activity) by computing the mutual information between the predicted distribution of spike trains across task epochs and the true distribution (Materials and Methods). Throughout this article, mutual information values are expressed as percentage of the maximum value corresponding to perfect discrimination. Information values were computed for different analysis windows, all starting 1 ms after feedback time and with increasing duration. In this way, the state of a putative decoder of dACC feedback-related discharges could be evaluated at different delays after the start of the decoding process.

#### Optimal temporal sensitivity mediates information improvement in a majority of single neurons

**Fig 3:**
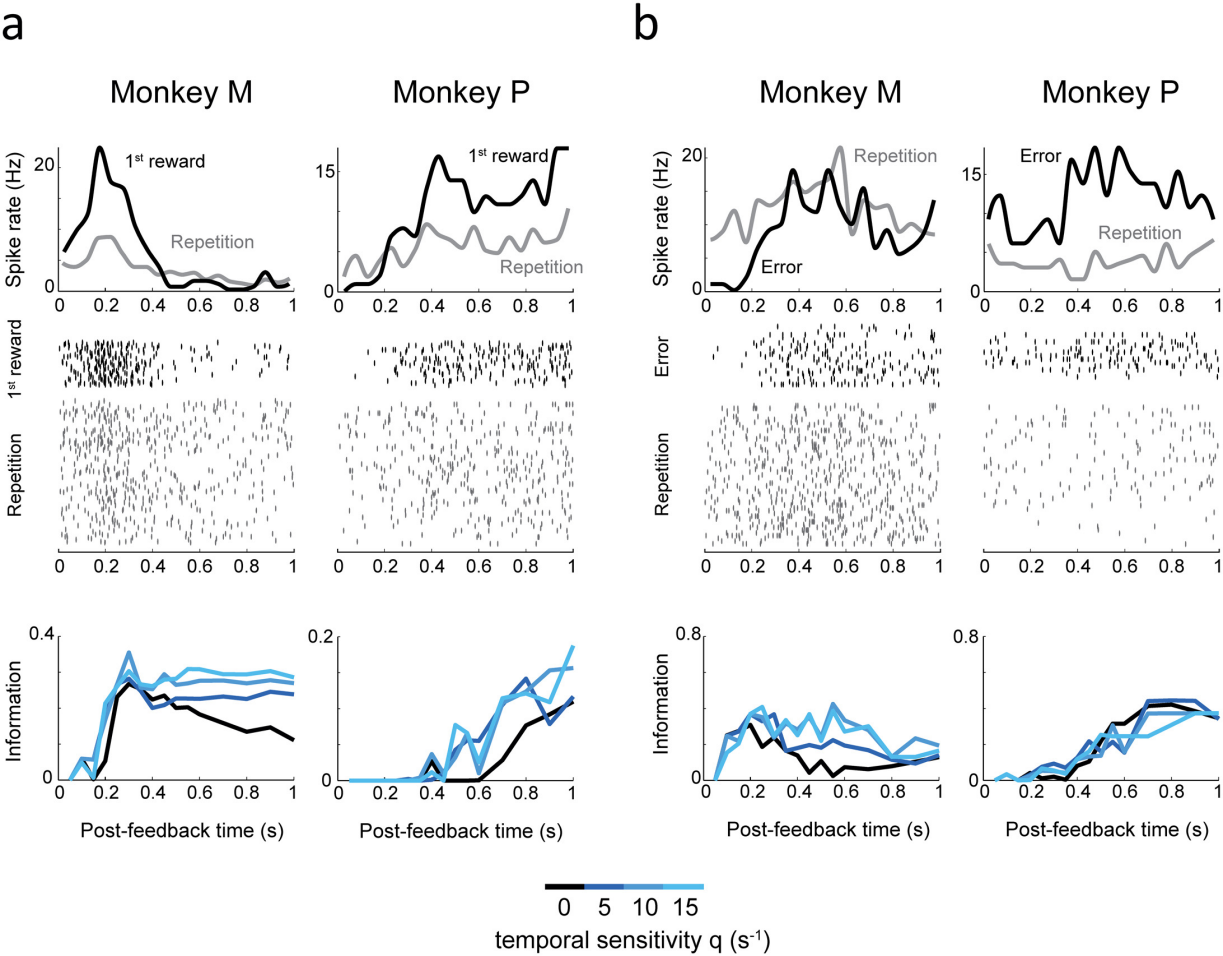
Examples of single-unit dACC activities decoded with different temporal sensitivities. **(a)** Spike densities (*top*) and raster plots (*middle*) during first reward (black curve) and repetition (grey curve) task epochs. The classification performance between first reward and repetition spike trains (i.e., information) is shown in the bottom graphs, the time in the abscissa being the time at which the analysis window (and thus, the decoding process) ends. Two neurons, from the two monkeys, are shown. These samples show that temporal sensitivity can improve classification performance. **(b)** Same as (a) but for errors and repetition in two other neurons from the two monkeys.

**Fig 4:**
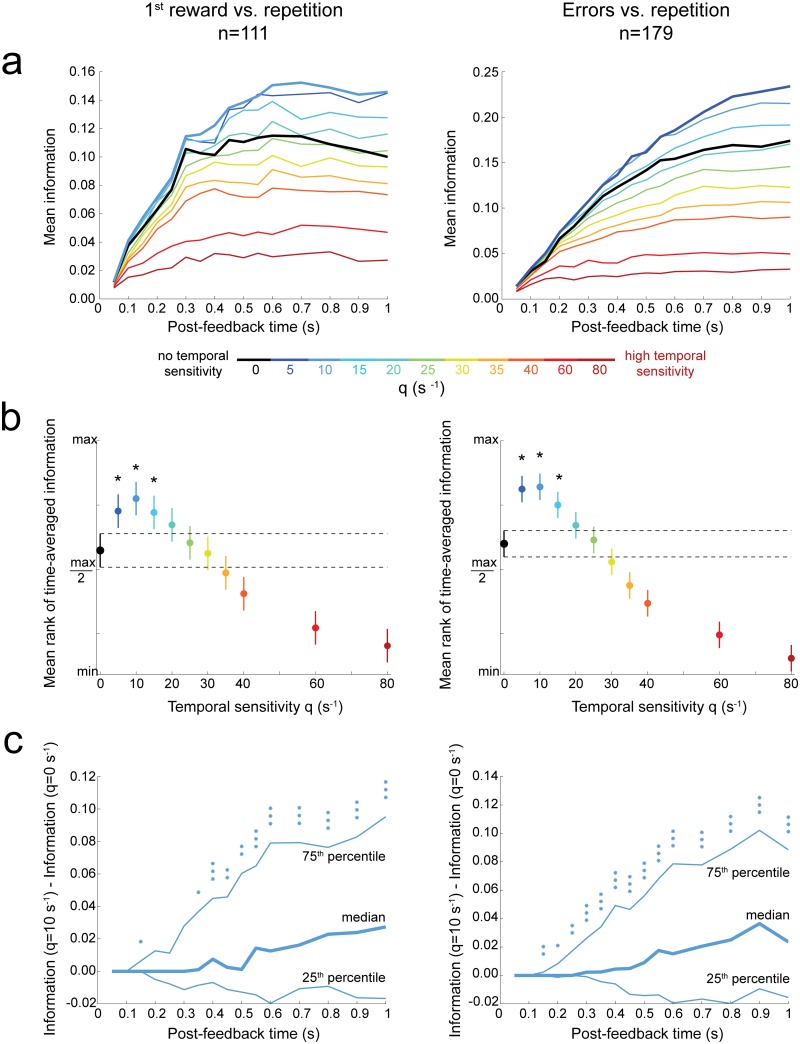
Optimal temporal sensitivity improves decoding of single unit behavioral adaptation signals. **(a)** Time course of the mean information (averaged among significant cells) as a function of the decoding temporal sensitivity (q). Information values were computed over increasing post-feedback time windows (ending at the time indicated by the *x*-axis). *Left*: Discrimination between first reward and repetition task epochs. *Right*: Discrimination between error and repetition task epochs. **(b)** Time-averaged information <I>_t_ (Table 1) for different temporal sensitivities (q). The ordinate axis is the normalized mean rank of <I>_t_. After a Friedman test, post-hoc comparisons with Tukey’s honestly significant difference correction were used for the 95% confidence intervals. Temporal sensitivities q > 0 that were performing significantly better compared to q = 0 are indicated by a star. **(c)** Distribution of the difference of information between optimal temporal decoding (q_opt_ ≈ 10 s^-1^) and spike-count decoding (q = 0 s^-1^). Asterisks indicate the significance of signed-rank tests (the null hypothesis is the symmetric distribution around zero): *, *p* ≤ 0.05; **, *p* ≤ 0.01; ***, *p* ≤ 0.001. See also S1–S7 Figs and S1 Table.

Consistent with previous results focusing on spike count only [29], we found that most dACC neurons with selective task-epoch activity fired more during behavioral adaptation periods (i.e., post first reward and/or error feedbacks) compared to reward in repetition (Fig 3 and S4 fig). We tested whether temporal sensitivity would consistently tend to improve information transmission among all neurons (which showed a certain variability in the detail of their response, see Fig 3), compared to spike count. Importantly, for most neurons, timing-sensitive decoding of spike trains (q > 0) conveyed more information than spike count (q = 0; Fig 4). We characterized this effect by looking at the time course of information (averaged across neurons with significant coding power: permutation test, *p* < 0.05) for different q_s_ (Fig 4a). For each q, the information increased as post-feedback spiking accumulated with time. Temporal sensitivity influenced both the maximum amount of information and the speed at which it increased. Importantly, adapted temporal sensitivity provided a sizable gain (15%–40%) in mean information compared to spike count. Values of q within [5,10,15]s^-1^ led to a significant increase in time-averaged information (Table 1 and Fig 4b; Friedman ANOVA, global effect on all considered q values: *p* < 0.001). This effect was robust early after the feedback and for all subsequent times (Fig 4c). The advantage of temporal decoding over spike count decoding was robust in both monkeys individually (S1 fig). Very small temporal sensitivities, compatible with an imperfect integrator with a slow decay, led to significantly less information than optimal temporal decoding (S2a and S2b Fig).

**Table 1:** Definition of statistical measures.

**Time-averaged information** ≡ 〈*I*〉_*t*_
**Difference in mean spike count between task epochs $\equiv {\langle N_{adapt}\rangle }_{trials}-\;{\langle N_{repet}\rangle }_{trials}$**
**Normalized absolute difference in mean spike count between task epochs $\equiv \;\frac{\left|{\langle N_{adapt}\rangle }_{trials}\;-\;{\langle N_{repet}\rangle }_{trials}\right|}{{\langle N_{adapt}\rangle }_{trials}\;+\;{\langle N_{repet}\rangle }_{trials}}$**
**Optimal timing sensitivity $q_{opt}\equiv mean\left(\underset{q}{\text{argmax}}\left({\langle I\rangle }_{t}\right)\right)$**
**Optimal distinction degree between units $k_{opt}\equiv mean\left(\underset{k}{\text{argmax}}\left({\langle I\rangle }_{t}\right)\right)$**
**Temporal structure related gain of information $\equiv \frac{\underset{q}{\text{max}}\left({\langle I\rangle }_{t}\right)-{\langle I_{q=0}\rangle }_{t}}{{\langle I_{q=0}\rangle }_{t}}$**
**Fano factor estimate $F\equiv \frac{{\text{var}}_{estim}\left(C_{t}\right)}{\langle C_{t}\rangle }=\frac{\frac{\sum_{i=1}^{n_{trials}}{\left(C_{t}^{i}-\langle C_{t}\rangle \right)}^{2}}{\left(n_{trials}-1\right)}}{\langle C_{t}\rangle }$**, where *C*_*t*_ is the random variable counting the number of spikes fired by a neuron in a given analysis windows during one task-epoch, and $C_{t}^{i}$ is its realization in a given trial among the *n*_*trials*_ available trials.
**Gain in the pair relative to the best single unit $\equiv \frac{\underset{q,\;k}{\text{max}}\left({\langle I^{pair}\rangle }_{t}\right)-\underset{cells,\;q}{\text{max}}\left({\langle I^{single}\rangle }_{t}\right)}{\text{max}\left(\underset{q,\;k}{\text{max}}\left({\langle I^{pair}\rangle }_{t}\right)\;,\;\underset{cells,\;q}{\text{ max}}\left({\langle I^{single}\rangle }_{t}\right)\right)}$**
**Information imbalance between two units $\equiv \frac{\left|\underset{q}{\text{max}}\left({\langle I^{cell\;1}\rangle }_{t}\right)-\underset{q}{\text{max}}\left({\langle I^{cell\;2}\rangle }_{t}\right)\right|}{\text{max}\left(\underset{q}{\text{max}}\left({\langle I^{cell\;1}\rangle }_{t}\right)\;,\;\underset{q}{\text{max}}\left({\langle I^{cell\;2}\rangle }_{t}\right)\right)}$**
For pairs with *k*_*opt*_ = 0:** Information gain when not distinguishing between neurons $\equiv \frac{\underset{q}{\text{max}}\left({\langle I_{k=k_{opt}=0}^{pair}\rangle }_{t}\right)-\underset{q}{\text{max}}\left({\langle I_{k=k\text{max}=2}^{pair}\rangle }_{t}\right)}{\underset{q}{\text{max}}\left({\langle I_{k=k_{opt}=0}^{pair}\rangle }_{t}\right)}$**
**Between-neuron spike coincidence index** $\equiv \text{max}\left(P_{adapt}^{intra},\;P_{repet}^{intra}\right)-P^{inter}$, where *P* is the proportion of trials for which between-neuron spike-matching(s) did impact the Victor and Purpura distance $d_{q_{opt},\;k_{opt}}$, for the analysis window that maximizes *I* ^*pair*^.

The angle brackets denote averaging; t denotes time average over the ensemble of analysis windows beginning 1 ms after the feedback and ending from 100 ms to 1 s (by steps of 100 ms). Information values I were always normalized and bias corrected unless mentioned. We therefore simply refer to them as “information” throughout the text. “*adapt”* stands for behavioral adaptation task epochs (either errors or first reward); “*repet”* stands for repetition task epoch. N is the spike count in a window between 1 and 1,000 ms after feedback onset. $\underset{y}{\text{argmax}\left(f\left(y\right)\right)}$ is the point y_o_ of the argument y for which the function f attains its maximum value.

We found similar results when decoding first reward versus errors (S7 and S2c Figs). This implies that decoding of both the appropriate behavioral strategy and the degree of necessity to update the behavior (Fig 4) could benefit from temporal sensitivity.

The curve of the amount of information versus q was bell-shaped (Fig 4b). This suggests an optimal range of temporal sensitivity for decoding. If q increases further, the decoder emphasizes too much small uninformative spike time fluctuations relative to the appropriate timescale(s) of spike-timing reliability, thereby deteriorating the decoding (Fig 4 and S2 Fig). Note that, among significant neurons, the interquartile ranges of the median interspike interval (computed within [0.001, 1] s post-feedback separately for all trials) were 54–143 and 49–110 ms for first reward and error discrimination, respectively. Consequently, several spikes often occurred within the range of spike timing reproducibility accounted for when decoding with q_opt_ ≈ 10 s^-1^ (i.e., a range of 200 ms, Fig 2a **right**). We stress that this temporal decoder was therefore more spike-time sensitive than a mere 200 ms binning procedure, because for q = 10 s^-1^, the whole range of interspike intervals between 0 and 200 ms corresponds to different values of dissimilarity (Fig 2a, **right**). This range of interspike intervals can be interpreted as the range of presynaptic spike time jitters at which coincidences happen, i.e., when Excitatory Post Synaptic Potentials (EPSPs) decaying at a time scale τ ≈ 100 ms can sum.

#### Temporal coding supplements, rather than competes with, spike count coding

We investigated the relationship between the firing rate properties of the neurons and temporal coding. The absolute value of the difference in mean spike count between task epochs (Table 1) correlated positively with the maximum time-averaged information (Spearman correlation coefficient: c _first reward_ = 0.57, c _errors_ = 0.71, *p* < 0.001 for all). However, large spike-count differences in highly informative neurons did not imply the absence of information related to spike timing (S3 Fig). Also, the normalized difference in mean spike count and the gain of information related to timing sensitivity (Table 1) were negatively correlated (c _first reward_ = -0.52, c _errors_ = -0.6, *p* < 0.001 for all). Therefore, temporal sensitivity could uncover a relatively high amount of information in neurons with small differences in spike rate between task epochs (such as the neuron on the left of Fig 3b).

The dissimilarity measure was cumulatively affected by spikes, which made the classification performance more influenced by the task epoch with more spikes. Interestingly, the optimal temporal sensitivity was higher in neurons that fired more during behavioral adaptation compared to neurons firing more during repetition (S4 Fig). For neurons firing more during repetition, optimal temporal sensitivities were distributed around q = 5 s^-1^. In contrast, for neurons firing more during behavioral adaptation, which were the majority, the median optimal sensitivity was 10 s^-1^ and 7.5 s^-1^ for first reward and error discrimination, respectively (with a significant improvement compared to q = 5 s^-1^ for first reward) (S4 Fig). These results may reflect a higher temporal reliability of spiking during behavioral adaptation. Alternatively, this result may indicate that the feedback time during repetition epochs provided a less reliable time reference for neural activity.

#### Sensorimotor differences between task epochs are not likely to determine the advantage of temporal decoding

Sensory or motor differences between task epochs were unlikely to determine the advantage of temporal decoding. In fact, external events (e.g., feedback, stimuli) were identical during first reward and repetition epochs. The motor influence on neural activity was also unlikely to cause temporal decoding advantage (S6 Fig, analysis possible in monkey M). Indeed, if the temporal structure of dACC activity were motor related, all eye movements timed differently between task epochs would favor temporal coding. To test this hypothesis, we removed all trials with a saccade occurring before approximately 0.65–0.85 s post-feedback, and kept the remaining approximately 20% of the trials. This manipulation did not decrease the advantage of temporal decoding of first reward versus repetition (S6d and S6e Fig: removing putative motor feedback activity; S6f and S6g Fig: removing also putative premotor activity). Following target fixation, late first saccades after first reward (approximately 850 ms after first reward delivery) also predicted that monkeys would be quicker to respond in the following trial (S6c Fig). Therefore, dACC neural activity occurring either before or during these late saccades may not reflect motor planning but rather cognitive correlates (e.g., attentional modulation). These trials with late first saccades indeed appeared to contribute to the temporal advantage for decoding (see analysis windows ≥650 ms in S6 Fig). Hence, the different temporal patterns between first reward and repetition task epochs probably originated from different internal neuronal dynamics.

### Temporal Decoding of First Reward Versus Repetition Spike Trains Does Not Only Rely on Differences in Time-Varying Firing Rate between Task Epochs

We investigated the nature of dACC firing statistics determining the advantage of temporal decoding. Spike-timing reliability might mainly reflect differences in the temporal variations of firing rates between task epochs. Alternatively, beyond this time-dependent firing rate, temporal correlations between spikes within one trial may impact the spike time reproducibility. Indeed, cellular processes (such as spike-triggered hyperpolarizing currents) may affect future spiking probability depending on past spike times [4,36], especially when the synaptic current received by the neuron is not very variable. Similarly, recurrent neural network dynamics—within dACC or upstream—may create correlations in spike times [37,38]. Here, we tested whether or not, beyond their existence, spike-timing correlations sizably and consistently (over neurons) impacted information transmission.

#### Assuming a time-dependent firing rate implies a spike count variability incompatible with the data

**Fig 5:**
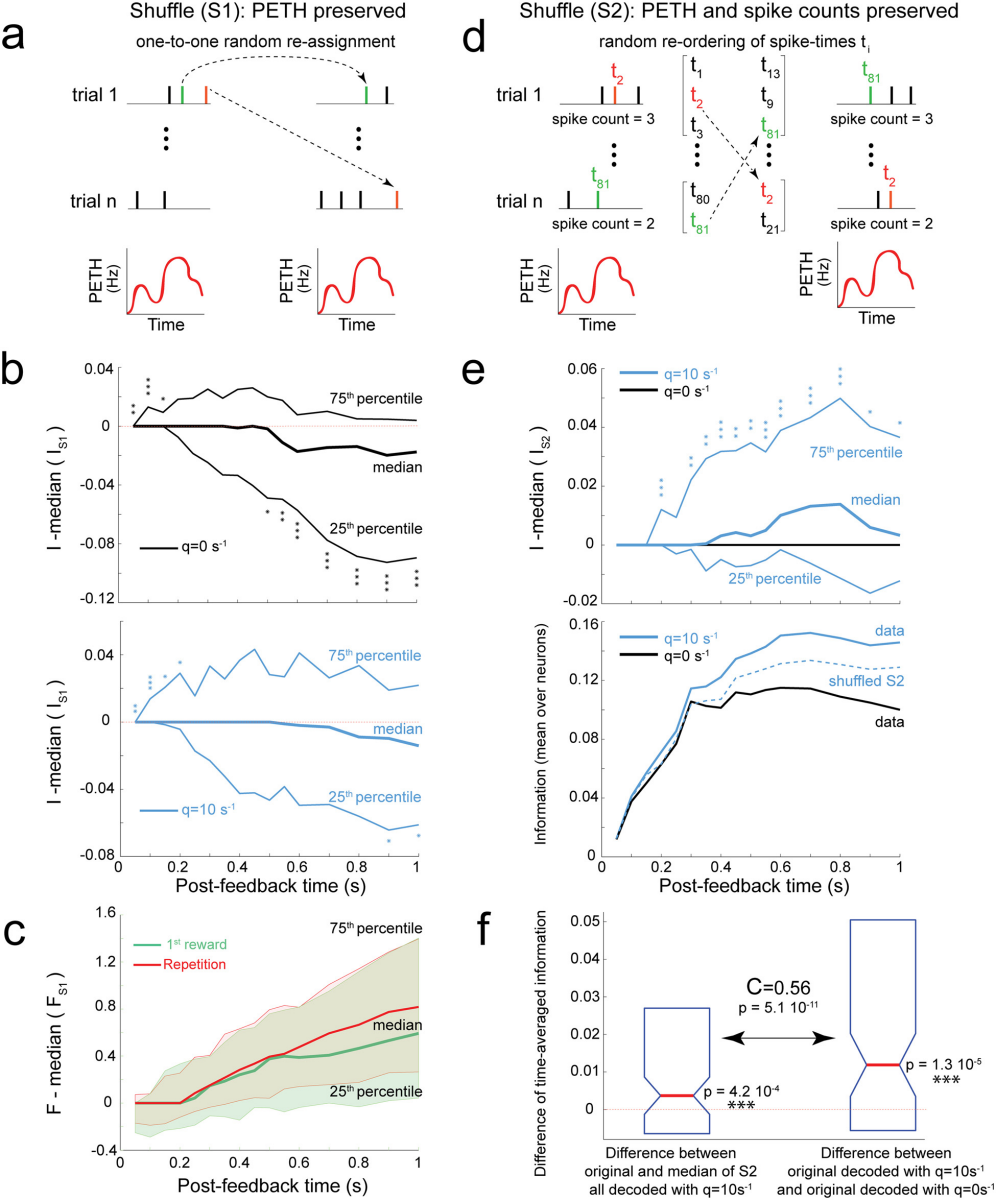
Temporal decoding does not only rely on differences in time-varying firing rate. This analysis was restricted to neurons significantly discriminating between first reward and repetition task epochs. **(a)** Shuffling of spikes between trials while preserving PETHs (i.e., time-dependent firing rates). This procedure was repeated 1,000 times within each task epoch and independently for each neuron. If information transmission in the data relies on PETHs, spike shuffling should not impact decoding. **(b)** Distribution of the difference between information I in original data and the median information in shuffled data (as in a). Asterisks indicate the significance of a signed-rank test: 1 to 3 asterisks, *p* ≤ 0.05, *p* ≤ 0.1, *p* ≤ 0.001, respectively. *Top*: spike-count decoding (q = 0s^-1^). *Bottom*: optimal temporal decoding at q_opt_ ≈ 10 s^-1^ (this q value maximized information averaged over neurons). **(c)** Difference of Fano factor estimate (F, Table 1) between original data and the median of 1,000 shuffled datasets for first reward (green) and repetition (red). **(d)** Shuffling of spikes between trials while preserving both PETHs and spike count variability. The shuffling is done 1,000 times, independently for all analysis windows, task-epochs, and neurons. All spikes emitted during different trials of a task epoch are grouped and their order shuffled. Each pseudo-trial (right) is created by taking from the shuffled spike pool (middle) the same number of spikes as in the corresponding original trial (left). If information transmission in the data were shaped by PETHs whose amplitude could change across trials, spike shuffling would not impact decoding. **(e)** *Top*: Distribution of the difference between information in original data and the median information in shuffled data (as in d). Note that for q = 0 s^-1^ the curves of median, 25th and 75th percentiles are overlapped. *Bottom*: mean information in the original data decoded with q_opt_ ≈ 10 s^-1^ and with q = 0 s^-1^, and in spike trains shuffled (as in d) decoded using q_opt_ ≈ 10 s^-1^. **(f)** Left boxplot: difference between time-averaged information <I>_t_ in original data and the median of <I>_t_ in shuffled data (as in d) at q_opt_ ≈ 10 s^-1^, with signed-rank p-value. Right boxplot: for comparison, the difference in time-averaged information between q_opt_ and q = 0 s^-1^ in original data. Box plots show 25th, 50th, and 75th percentiles. The two quantities (left and right boxplots) were correlated (with coefficient C). See also S8 Fig.

We randomly shuffled spike times within each task epoch while preserving the peri-event time histogram (PETH) for each neuron (Fig 5a). This transformation preserved the time-dependent firing rate, while destroying temporal correlations. It also created a “Poisson” spike count variability, i.e., purely determined by random samples from a unique time-dependent firing probability (Materials and Methods).

If information transmission were shaped by time-dependent firing rates, original and spike-shuffled data should convey similar information. In contrast, we found that both spike-count and timing-sensitive decoding at q_opt_ ≈ 10 s^-1^ were more reliable for short analysis windows, and less reliable for long analysis windows, in the original compared to shuffled data (Fig 5b). These results were consistent and robust in both monkeys (S8a Fig). Timing-sensitive and spike-count decoders were both impacted by changes in spike count variability. For short analysis windows, the improved reliability of spike count in the original data could be enhanced by spike-triggered hyperpolarizing currents, which counterbalance random deviations of neuronal excitability in single neurons [36,39]. For long analysis windows, spike count appeared more variable in the original data (as measured by the Fano factor; Fig 5c), causing a smaller decoding reliability.

This means that spike count variability in the original data cannot be explained by random samples taken from a single firing probability. More precisely, for post-feedback times longer than 500 ms, the spiking probability was actually stronger in some trials than in other trials. This suggests a hidden source of spike count variability across trials that is not constant during one task epoch and has a major influence on information transmission [37,40]. This large spike count variability may reflect the integrative properties of dACC. Indeed, beyond signaling the need for behavioral adaptation, dACC firing may also be influenced by factors such as attention [16] and target identity [41]. Interestingly, this large spike count variability appeared to hinder more spike count decoding (Fig 5b). Hence, this may have participated in shaping the larger difference of information between q_opt_ ≈ 10 s^-1^ and q = 0 s^-1^ decoders that occurred for long analysis windows (≥500 ms) compared to short windows (Fig 4a–4c).

#### Temporal correlations considerably impact information transmission

We tested whether PETH and spike-count variability of the data could shape information transmission. To do so, we shuffled spike times while preserving both PETHs and spike counts in all trials (Fig 5d). By this operation, spike-count information was conserved in the shuffled data. If temporal correlations had negligible impact on information transmission, then temporal decoding should also remain unchanged. In contrast, we found that for q_opt_ ≈ 10 s^-1^, information decreased in the shuffled data as compared to original ones (Fig 5e and 5f). These results were robust and consistent among monkeys (S8 Fig).

The temporal correlations of the original data increased information by about 10%–15%, on average, compared to shuffled data (Fig 5e, information at plateau). In addition, the increase of information with optimal temporal sensitivity q_opt_ (compared to spike count) was significantly correlated to the information increase with temporal correlations (Fig 5f). This further suggests that temporal correlations tended to support temporal coding. Finally, information loss after spike shuffling was larger for larger temporal sensitivities q (S8d Fig).

These results suggest that, beyond PETHs, temporal correlations led to spike timing reliability that favored task-epoch discrimination in most neurons. This could reflect either the effect of spike-triggered hyperpolarizing currents in single neurons, or network dynamics mediating the behavioral strategy signal and making future spike times dependent on spiking history [37,42,43].

### Temporal Patterns Often Differ between Neurons, Implying a Spatiotemporal Code

Multiple neurons were often simultaneously recorded (median = 2). Thus, we also decoded the activity of pairs of neurons (Monkey M, *n* = 122 pairs; Monkey P, *n* = 271) while varying both the temporal sensitivity q and the degree of distinction k (Fig 2b, Materials and Methods). For the computation of the dissimilarity measure, the parameter k represents the cost of transforming a spike from neuron 1 into a spike from neuron 2. Therefore, during classification of spike trains from pairs of units, the dissimilarity between spikes from different neurons increases with k.

The parameter k permits testing of whether the informative spikes are neuron specific or if they tend to be emitted synchronously by two neurons. In the former case, the amount of information would increase if the decoder were accounting for neural identity (k > 0), as compared to a decoder blind to neural identity and sensitive to interferences between neurons (k = 0). In the latter case, k = 0 could be optimal for decoding because it makes the discharge of either one of the neurons sufficient to have reliable joint spiking.

#### Paired decoding benefits from an optimal distinction of the spikes from the two neurons

**Fig 6:**
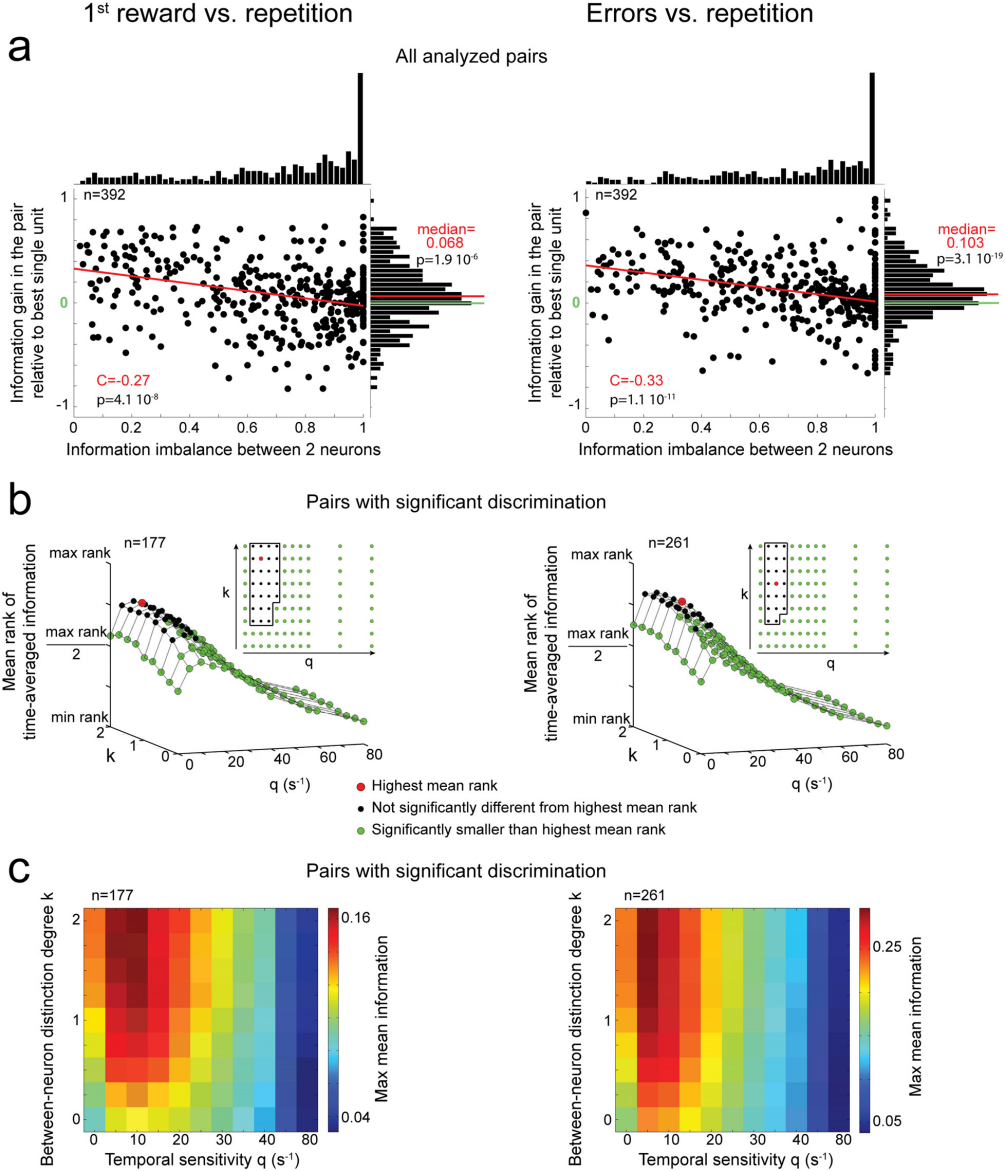
Efficient paired decoding often requires distinguishing between the activities of the two neurons. *Left*: Decoding first reward versus repetition task epochs. *Right*: Decoding error versus repetition task epochs. **(a)** Distribution of information gain when decoding a pair of units relative to decoding the isolated unit of the pair with the highest information, as a function of the information imbalance between the two units of the pair (Table 1). The red line indicates a linear regression fit. The distributions of information gains were significantly biased toward positive values, as indicated by a signed-rank test (all p_s_ < 10^−5^). **(b)** Mean rank comparison (with Friedman ANOVA) of the time-averaged information <I>_t_ as a function of (q,k). Data were pooled from both monkeys and were restricted to pairs with significant information. **(c)** Maximum mean (over neurons with significant information) information as a function of (q,k). Information was maximized over analysis windows ending in [0.05, 0.6] s, steps of 50 ms, and in [0.7, 1] s, steps of 100 ms. See also S9 and S10 Figs.

We first tested whether decoding with optimal (q, k) values advantageously combined the activity of any two analyzed neurons (regardless of their individual coding properties). This was not trivial because of the large imbalance in information between neurons (Fig 6a). Also, when some noise caused one neuron to fire more, it was not causing the other neuron to fire less (i.e., spike counts were not negatively correlated in our data). Thus, in general, summing the activity of two neurons would not cancel the effect of noise on spike counts. Indeed, the (wide) distribution of spike-count correlations between two neurons was slightly positively biased during first reward or repetition (signed-rank test on time-averaged correlation coefficients: *p* = 1.6 10^−3^ with median 0.043 for first reward; *p* = 1.4 10^−5^ with median 0.036 for repetition). During errors, the distribution of correlation coefficients was centered on zero.

In general, a simple sum of two independent or positively correlated neural activities with very different standard deviations is likely to decrease the signal-to-noise ratio, compared to the more reliable single activity. By contrast, on average we found positive gains of information when decoding a pair compared to just decoding the most informative neuron of the pair (Table 1, “gain in the pair relative to the best single unit”; Fig 6a, signed-rank test, all *p*_*s*_ < 0.001). As expected, information gains negatively correlated with the information imbalance between paired neurons (Fig 6a, Spearman correlation with permutation test, all *p*_*s*_ < 0.001; more pronounced for pairs with significant coding: S9 Fig).

We then investigated which (q, k) values yielded better dACC decoding. For any k values, the time-average information 〈*I*〉_*t*_ significantly increased with temporal sensitivity up to q_opt_ ≈ 10s^-1^ and decreased for larger q values (Fig 6b and 6c). Hence, for any k, spike-count decoding (q = 0s^-1^) led to a significantly lower 〈*I*〉_*t*_ than optimal temporal sensitive decoding (q_opt_ ≈ 10s^-1^). 〈*I*〉_*t*_ also increased with k and plateaued at about 1. Therefore, intermediate to high levels of distinction between spikes from paired neurons often improved the decoding of behavioral adaptation signals, suggesting that some reliable spikes were neuron specific. Differences in information average (over significant pairs) across (q, k) values were consistent over time and between monkeys (S10 Fig).

#### Jointly recorded neurons can share similar temporal firing patterns

Decoding with intermediate k values may imply temporal coincidence between spikes from two different neurons as opposed to between spikes from the same neuron. We found that spike coincidence between two neurons occurred, on average, in 34% (first reward) and 41% (errors) of all pairwise comparisons between spike trains (computed as for the last entry of Table 1).

In addition, we computed an index quantifying the spike coincidence between neurons within a task epoch (Table 1). This index negatively correlated with optimal k values, k_opt_, (c _first reward_ = -0.71, c _errors_ = -0.54, *p* < 0.001). k_opt_ values were pair specific rather than shared among most pairs as for q_opt_. For instance, k_opt_ values were smaller for pairs of units that fired preferentially in the same task epoch relative to pairs of units with opposite firing preferences (ranked-sum test, all p_s_ < 0.01; median k_opt_ values were 0.75 versus 1.25–1.5 for pairs with the same versus different firing preference). These results suggest that two neurons with similar firing preferences across task epochs were likely to have similar firing temporal patterns.

**Fig 7:**
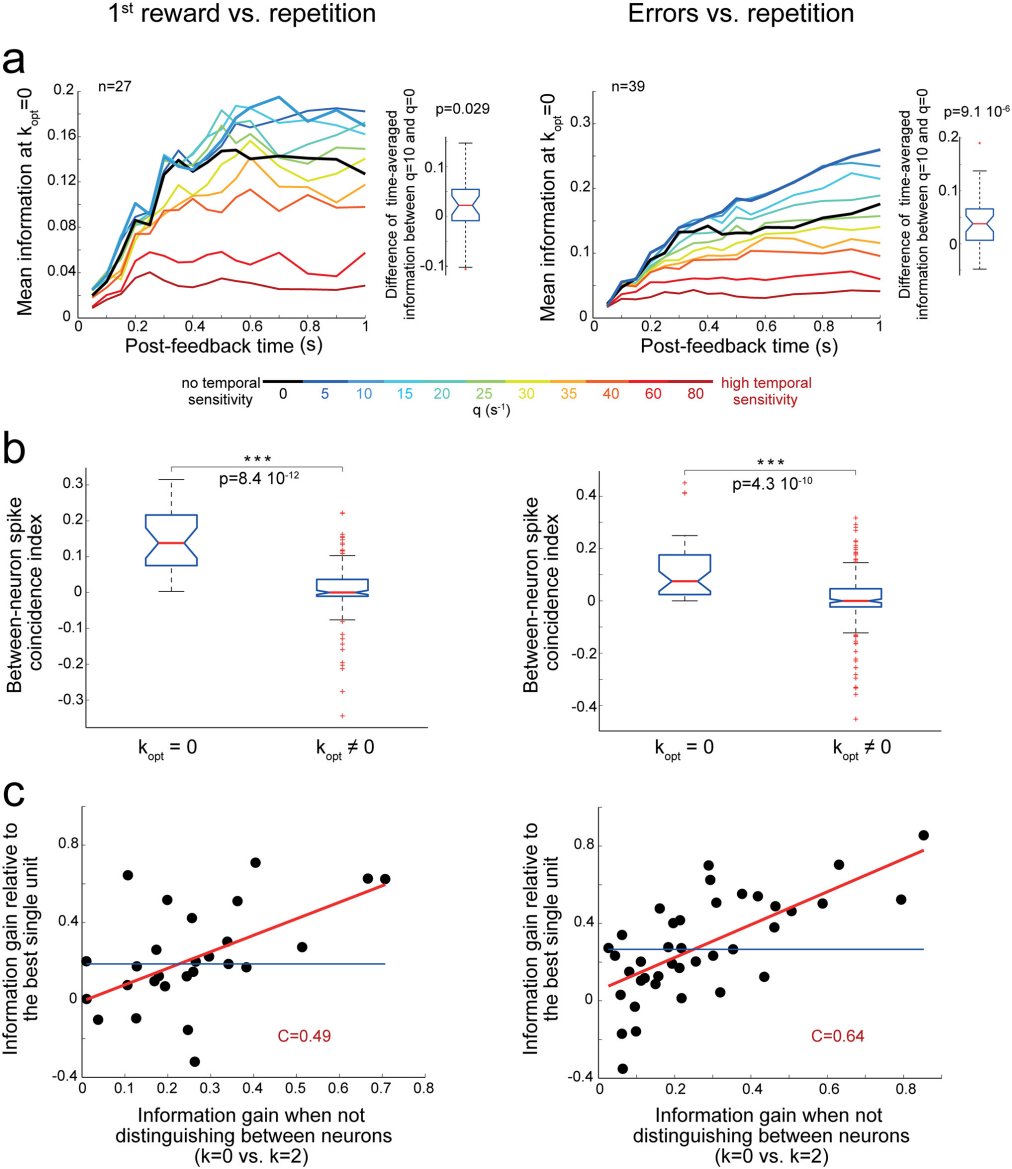
Coding properties of neuron pairs for which k_opt_ = 0. *Left*: Discrimination between the first reward and repetition task epochs. *Right*: Discrimination between error and repetition task epochs. **(a)** *Left*: Mean information among pairs with k_opt_ = 0 (significant encoding) as a function of the duration of the analysis window and of the temporal sensitivity (q). *Right*: Distribution of differences in time-averaged information <I>_t_ between q_opt_ = 10 and q = 0 s^-1^ (for k_opt_ = 0). The distribution has a significantly positive median (signed rank test). **(b)** The index of spike coincidence between neurons was higher for pairs with k_opt_ = 0 compared to other significant pairs (ranked-sum test, *p* < 10^−9^). Note that the median indexes were larger than 0 for pairs with k_opt_ = 0. This means that when comparing spike trains within one task epoch, coincidences between neurons occurred more often than when comparing spike trains between task epochs (Table 1). **(c)** The information gain relative to the most informative single unit was positively correlated with the information gain induced by the absence of neuron distinction. C: Spearman correlation coefficient, red line: linear fit, blue line: median of the distribution of information gains.

Some pairs of neurons had maximal 〈*I*〉_*t*_ when the decoder did not distinguish between the two single units (k_opt_ = 0; 15% of significantly informative pairs, corresponding to 7% and 10% of all analyzed pairs for first reward and error discrimination, respectively). These pairs transmitted more information with q_opt_ ≈ 10s^-1^ compared to spike count, q_opt_ ≈ 0s^-1^, (Fig 7a; signed-rank test on 〈*I*〉_*t*_: first reward discrimination, *p* = 0.029; error discrimination, *p* < 10^−5^). They had an index of spike coincidence between neurons larger than in other pairs (Fig 7b, ranked-sum test: all *p* < 10^−9^).

In these pairs, the information gains relative to the most discriminative unit of the pair were relatively high (Fig 7c). This suggested that these pairs were decoded efficiently. We tested whether these information gains were related to the gain of information when not distinguishing between neurons (i.e., k_max_ = 2 versus k_opt_ = 0; Table 1). We found a positive correlation (Fig 7c), suggesting that spike coincidence between neurons could mediate an efficient combination of their activities.

Hence, on the one hand, the information generally increased when the identity of the neurons was accounted for (intermediate-to-high k_opt_ values), which indicates that reliable spike times were variable and distributed across the neuronal population. On the other hand, some pairs of neurons with similar temporal firing patterns could be efficiently decoded through between-neuron temporal coincidences (Fig 1c).

### The Temporal Structure of Single Unit Spike Trains Predicts Behavioral Response Times

The presence of information in single-unit spike timing does not necessarily imply that the downstream networks do actually use it [27,28]. In particular, if dACC spike timing were not used, then different temporal patterns would be rather unlikely to reliably correlate with different behavioral outputs. Here we examined whether first reward single-unit activity could predict upcoming behavior. We focused on the behavioral response time, i.e., the time between the “go” signal and touch on target (Materials and Methods). The response time was measured during the trial following the first reward. Thus, several seconds separated the analyzed neural activity and the behavioral measure. Interestingly, the response times of both monkeys consistently increased on the touch following the first reward compared to the touch leading to first reward (S11c Fig). This was in agreement with a behavioral switch from exploration to repetition.

We separated trials into two groups: one group with response times higher than the median, and the other with response times below the median. The probability of switching to repetition was very high in both groups and statistically equivalent between them (S2 Table). We tested the hypothesis that longer response times may reflect a longer decision-making process, when monkeys might act more carefully to avoid mistakes.

#### Firing rate increase does not robustly relate to a behavioral response time change

In the context of a neural integrator decoder to maintain a memory of the necessity to adapt the behavioral strategy, one could expect that the spike count would be directly predictive of the behavioral response time. Indeed, in this scenario, the downstream decoder would receive an overall excitatory input from the population of dACC neurons whose activity distinguishes between first reward and repetition task epochs, as this population fires more on average during first rewards (S4 Fig). Hence, any decrease in the number of spikes received by the decoder would hinder reaching the decision-making threshold (see “adapt behavioral strategy threshold” in Fig 1c). Conversely, any increase in spike input would accelerate threshold crossing (Fig 1c).

**Fig 8:**
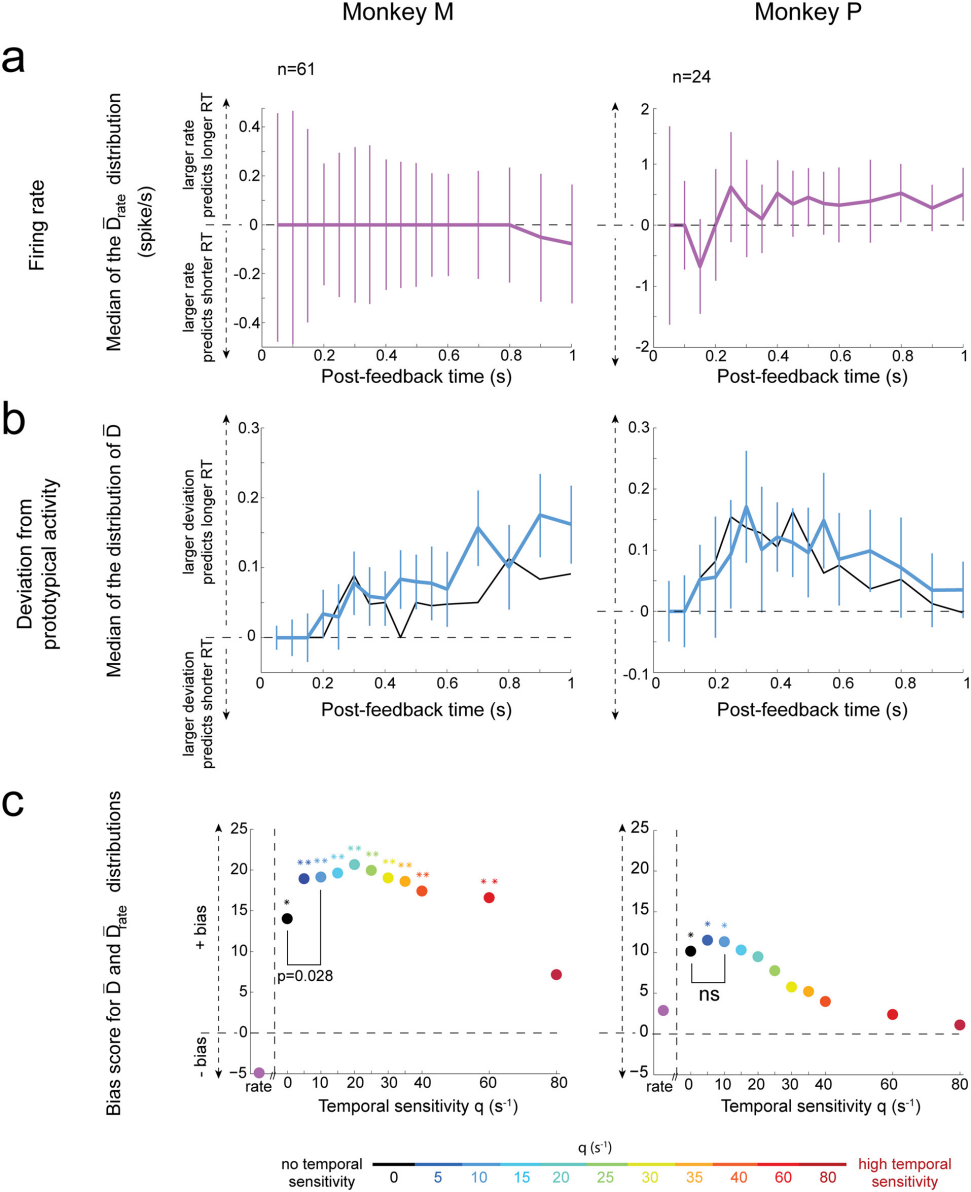
The temporal structure of single unit spike trains predicts behavioral response times. Left: Monkey M (all significantly informative neurons for first reward versus repetitions). Right: Monkey P (significant neurons with information ≥ median; neurons with very little information did not permit robust behavioral prediction in this monkey, see main text and S12 Fig). Analysis windows end at the time indicated by the *x*-axis. **(a)** Test for the neural integrator decoder receiving excitatory inputs from dACC feedback-related neurons. Time course of the median ${\bar{D}}_{rate}$ (difference in mean firing rate between trials with slow and fast response times). The value of ${\bar{D}}_{rate}$ is positive if trials with high rates tend to be followed by long response times. Bars represent the median confidence interval (+/-$\frac{\text{interquartile range}}{1.075\sqrt{n}}$, where *n* is the number of neurons). **(b)** Test for the spatiotemporal decoder. Time course of the median $\bar{D}$. The value of $\bar{D}$ is positive when spike trains emitted in first reward trials followed by slower response times deviate more from prototypical spike trains than those emitted in trials followed by fast response times. The two curves correspond to q = 0 (black) and q = 10 s^-1^ (blue). **(c)** Bias scores (across different analysis windows) for $\bar{D}$ and ${\bar{D}}_{rate}$. A large positive bias score indicates that the data is very positively skewed (relative to a distribution that is symmetrically distributed around 0). Asterisks indicate significance values for these biases (2-sided permutation test: *, *p* ≤ 0.05; **, *p* ≤ 0.01). For Monkey M, the lowest *p*-value was for q = 20 s^-1^ (*p =* 0.003); for Monkey P, the lowest *p*-value was for q = 5 s^-1^ (*p =* 0.029). Finally, the result of the comparison of $\bar{D}$, averaged over different analysis windows, between q_opt_ ≈ 10 s^-1^ and q = 0 s^-1^ is shown (signed-rank test). See also S2 and S11–S13 Figs.

Hence, given the two groups of trials (slow versus fast response times), we tested whether dACC neurons fired more in one of these two groups. We computed the difference in mean firing rate between spike trains that preceded trials with slow versus fast response times (${\bar{D}}_{rate}$, Materials and Methods). We found that the distribution of ${\bar{D}}_{rate}$ was not significantly skewed either positively or negatively, indicating that large firing rates in dACC neurons were not predictive of future monkey’s response times (Fig 8a and 8c; see also S12 Fig).

In addition, under the neural integrator decoding hypothesis, the dACC neurons firing more during first reward (approximately 70% of significant neurons) are expected to be the main drivers of firing rate increase in the decoder. To examine this, we restricted the ${\bar{D}}_{rate}$ analysis to neurons firing more during 1 s after the first reward (compared to repetitions). We found that this restriction did not lead to a more robust bias of the ${\bar{D}}_{rate}$ distribution (2-sided permutation test: monkey M, *n* = 42, bias score = -5.50, *p* = 0.28; monkey P, *n* = 18, bias score = -0.337, *p* = 0.93).

Finally, we also examined a scenario in which the downstream integrator decoder would receive excitatory inputs from neurons discharging more during first reward, and inhibitory inputs from neurons discharging more during repetition. We simply reversed the sign of ${\bar{D}}_{rate}$ for those neurons discharging more during repetition. However, we did not find a robust bias of the overall resulting distribution (2-sided permutation test: monkey M, *n* = 61, bias score = -4.14, *p* = 0.37; monkey P, *n* = 24, bias score = -3.0, *p* = 0.48; the same neurons as for Fig 8 are used).

#### Deviations from prototypical temporal firing patterns better predict response times

Under the hypothesis of a temporal decoder (Fig 1c), the success of information transmission relies on matching the discharge received during a particular trial with a prototypical activity pattern specific to a given task epoch. The robust classification of single-unit spike trains (Fig 4 and S4 Fig) indeed implies that during many first reward trials, the activity resembled a prototypical firing pattern specific to a feedback triggering behavioral adaptation. However, the classification was not perfect, which suggests that there were also trials during which the activity deviated from the prototypical temporal firing pattern. This could lead to inefficient information transmission, and then slower processing.

To test this, we developed a new method to estimate how much each single-trial spike train emitted by each neuron at first reward deviated from its “prototypical” discharge (i.e., its more common firing pattern during first reward; Materials and Methods and S4 Text). According to our method, for each neuron, a large positive deviation from prototype can occur when the spike count is either higher or lower than the neuron’s average rate, or when the spike times are jittered compared to the neuron’s usual temporal pattern. Hence, when computing the deviation based on q = 0, high values of deviation will in general be attributed to trials with both large and small spike count relative to average (as long as the spike count distribution is not overly skewed, with outliers lying on one side only).

Again, we considered the two groups of trials (i.e., associated with slow versus fast response times). For each neuron, we computed the difference in mean deviation from prototypical activity ($\bar{D}$) between these two groups. Notably, the distribution of $\bar{D}$ values was positively skewed (Fig 8b and 8c), indicating that a larger deviation from prototypical activity predicted a longer behavioral response time. This effect was consistent in both monkeys and between different subpopulations of neurons (S12 Fig). For monkey P, statistical robustness was reached when neurons with very little first reward versus repetition information 〈*I*〉_*t*_ were removed (Fig 8 and S12 Fig). Temporal sensitivity values leading to best dACC decoding (Fig 4) were also relevant for predicting behavioral response times. More precisely, q values of 5 and 10 s^-1^ led to a robust and significant bias of the distribution of $\bar{D}$ values in both monkeys (Fig 8c and S12 Fig). This result suggests that in both monkeys the temporal patterns of spikes could be relevant to downstream decoding areas ultimately adapting behavioral responses. We note that the difference of mean deviation from prototype based on spike count ($\bar{D}(q=0)$) was also significantly biased in both monkeys. Importantly, we confirmed that this effect was not likely to be merely caused by a difference in firing rate between trials with slow and fast response times with different signs in different neurons. Indeed, if the latter hypothesis were true, then the neurons with a large $\bar{D}(q=0)$ would also be expected to be those for which the absolute value of ${\bar{D}}_{rate}$ was large, leading to a strong correlation between $\bar{D}(q=0)$ and $abs\left({\bar{D}}_{rate}\right)$. Instead, we found that this correlation was small (Spearman correlation between time-averaged $\bar{D}(q=0)$ and $abs\left({\bar{D}}_{rate}\right)$, monkey M: c = 0.31, monkey P: c = 0.06). This suggests that, for any single unit showing large $\bar{D}(q=0)$ and small $abs\left({\bar{D}}_{rate}\right)$, the trials followed by long response times were likely to be associated with both increased (during some trials) and decreased (during other trials) spike count compared to prototype.

We then compared the deviations from prototype based on either spike count or temporal sensitivity q_opt_ ≈ 10s^-1^ (optimal to discriminate first reward versus repetitions). For monkey P, we found that spike count and temporal decoding performed equally well (signed-rank test on average $\bar{D}$ over analysis windows ending from 0.1 to 1 s by increments of 0.1 s). The two decoding strategies probably relied on different neurons as $\bar{D}$ values were considerably different between *q*_*opt*_ and *q = 0* (S4 Text, section 3). In monkey M, optimal temporal sensitivity significantly improved the relation between single-unit spike trains and upcoming response times compared to spike count (*p* = 0.028; Fig 8c and S2d Fig). Altogether, these results further argue in favor of the relevance of temporal spiking patterns for behavioral adaptation.

Note that a longer response time was associated with a higher probability of interruptions in the task (e.g., breaks in fixation) during the trial ending with this response (S2 Table). However, the correlation between response time and neural activity was not entirely caused by a difference between interrupted versus uninterrupted trials. Indeed, when we removed interrupted trials we still observed a significant positively skewed $\bar{D}$ distribution (S13 Fig).

Note also that our results could not be explained by a segregation of first reward responses into 4 equidistant clusters corresponding to the 4 targets. Indeed, under this hypothesis, all spike trains should have had similar values of neural deviation from prototypical activity, as the latter is averaged over all spike trains associated to all targets. Therefore, in this case, we should not observe our Fig 8b and 8c results showing significant differences of deviation from prototypical activity between different groups of spike trains. This suggests that dACC activity was not merely related to movement coding (see also S11 Fig). Rather, our results indicate that the deviation of dACC activity from prototypical temporal patterns could mediate a behavioral adaptation process modulating the delay of upcoming decisions and actions.

## Discussion

Post-feedback spike counts in dACC neurons were shown to depend on whether behavioral adaptation was required [29]. Given the absence of stimulus-driven temporal fluctuations in input and high noise in spike timing, a plausible hypothesis would be that only spike count is relevant to the transmission of the need to adapt behavior by dACC firing [2]. By contrast, we provide evidence for an efficient spatiotemporal spike coding of behavioral adaptation signals. Our analysis accounts for the temporal sensitivity of a biologically plausible neural decoder that would receive post-feedback dACC discharges. Adjusting the temporal sensitivity of the decoder (within the interval of one second after the feedback) can enhance the readout of single-unit spike trains relevant to behavioral adaptation. Beyond the existence of a temporal patterning of dACC activity, these results indicate that spike-timing reliability supplements spike-count reliability.

Interestingly, in frontal areas single-unit spike generation mechanisms or network dynamics, rather than external stimuli or motor feedback, are probably responsible for spike timing reliability and spike-count variability [37,40,43,44]. We found strong temporal correlations, stronger-than-Poisson spike count variability, and heterogeneous spike times across the dACC population. This feedback-type-specific dynamics is thus unlikely to arise through neuronal assemblies connected by balanced excitatory and inhibitory inputs with uniform wiring probability and with stationary weak-to-moderate strengths [37,40], as these features tend to create Poisson-like spike trains. Spike-triggered hyperpolarizing currents or short-term plasticity could also plausibly favor the presence of informative temporal correlations in dACC activity and could participate to shaping the lower-than-Poisson spike count variability occurring shortly after the feedback [36,39,44].

Note that the optimal range of decoding time scale that we found (τ ≈ 70–200 ms) is larger than those found when decoding responses to stimuli with relevant temporal patterning or contrast at onset time (e.g., auditory stimuli, τ ≈ 5 ms [45]; visual stimuli, τ ≈ 10–100 ms [13,35]). This is consistent with the idea of a hierarchy of increasing time scales from sensory to higher-order areas [46]. However, there are also exceptions to this rule, for instance in the gustatory modality (for which the timing of the stimulus is less relevant). Indeed, the optimal time scales were found to be close to the one we found in dACC (τ ≈ 50–500 ms [47]). Given that during a gustatory stimulation, the motor behavior of the animals and/or some sensorial input transients were probably participating in shaping the temporal code [47], it is quite remarkable that we found equivalent time scales in our data for which internal neuronal dynamics was probably the major contributor to spike timing reliability.

Also, the optimal spike coincidence timescale we observed loosely matches the period of local field potential (LFP) oscillations in the delta and theta range, on which frontal neurons can phase lock during cognitive tasks [16,19,48]. LFPs partially reflect the synaptic input of the local population [49], which could both shape and be influenced by the temporal spiking patterns of dACC.

The optimal temporal sensitivity range for decoding identified in this study remains an approximation. First, different methods (S1 and S4 Texts), or different analysis windows (Fig 4), might give slightly different optimal values. Yet, although it is not feasible to extensively test all possible decoders, our analysis accounts for biophysically reasonable assumptions on the downstream decoder. In this framework, we provide strong evidence for the plausibility of decoding through spike coincidences (up to a few hundred ms), compared to a neural integrator decoder. Second, spike trains were referenced to feedback time, but the internal reference of the brain could be different and more or less accurate [50] (e.g., coincidence detection during a population onset [24], or precise spike timing relations in a neuronal population [51]). Aligning to feedback times was very relevant for behavioral-adaptation task epochs where monkeys could not predict the outcome and were thus reacting to feedback. However, anticipation of rewards during repetition periods may have promoted internal references dissociated or jittered from actual juice delivery, decreasing the apparent temporal reliability (as suggested by the data, see S4 Fig).

The spike-time sensitive decoder can be understood as a downstream network that, through synaptic plasticity [52], becomes differentially selective to coincident spiking patterns that are specific to task epochs. The optimal temporal sensitivity range is compatible with the time constant of NMDA-mediated currents. Indeed, the efficiency of the spike coincidence mechanism decreased with interspike intervals up to approximately 200 ms, which relates to an exponential decay time-constant of 100 ms.

Within the decoding by coincidence framework, decoding relies on the convergence of excitatory neurons that transmit similar temporal patterns to a post-synaptic compartment (triggering summation of depolarizations). Yet, informative neurons with distinct and potentially antagonistic temporal patterns may improve information transfer, for instance, if they were decoded by different specialized post-synaptic neurons. We showed that paired decoding generally enhanced information transmission relative to the pair’s most discriminative unit. This suggests that highly informative activity can be advantageously combined with less informative inputs that do not act as contaminating noise. The information increase was achieved by varying the degree of distinction between the two units (parameter k). This mechanism may be implemented by different spatial organizations of synapses, which could modulate, through non-linear summation, the temporal precision of spike coincidence detection. Other mechanisms, such as different synaptic weights or synaptic timescales (i.e., two weak/shorter depolarizations that require more precise coincidence to efficiently sum), or targeted inhibition, may also induce a similar effect. In addition, we showed that in a smaller proportion of pairs the activity of both units did not need to be distinguished to achieve optimal discrimination. Thus, if these two units were excitatory, direct summation of their post-synaptic potentials would be advantageous. The partial spatial specificity of reliable spikes may be advantageous during realistic decision-making when quick choices should be made between many strategies. Indeed, the combination of spatial and temporal information can increase the number of possible specific activity patterns compared to simultaneous firing of all neurons.

Artifacts of spike sorting were unlikely to affect our conclusions (S2 Text). The removal of (rare) coincident spikes during sorting decreased the reliability of both spike count and temporal patterns. Noisy spikes erroneously assigned to a given neuron can make spike count more reliable whenever two mixed single units have the same firing preference (with respect to task epoch). On the other hand, temporal coding may only become more reliable if the two mixed units are temporally coherent. Our results showed that different dACC units did not often share coherent spike times, which suggests that possible erroneous spike assignments would not favor temporal decoding. Furthermore, pairs recorded on the same electrode did not always differ from pairs recorded on different electrodes (S3 Table). Indeed, while no differences occurred when discriminating first reward versus repetition task epochs, differences could exist for error versus repetition decoding. When present, such differences were more likely to reflect a topological organization of some inputs. Indeed, a by-product of a cross-talk between sorting templates would rather have a constant impact across all task epochs.

We further probed dACC function by testing how it could affect future behavior. We found a significant correlation between neural activity at feedback time and the monkeys’ response time during the following trial. This finding is functionally different from the correlation previously reported between pre-movement dACC activity (which often resembles an integration to threshold [53,54], in contrast to feedback-driven dACC responses) and immediate motor response [53–55]. This motor correlation could become apparent through the comparison between trials with high versus low firing rates (or, equivalently, spike-counts in a given window). In particular, Michelet et al. showed that the quicker the increase of firing rate to threshold, the quicker the movement [53]. This implied high versus low spike-count correlation when aligning spike trains with respect to movement. In contrast, we observed a correlation between dACC activity and behavior in terms of deviation from prototypical activity patterns, while we did not observe a robust link between large versus small number of spikes emitted during first-reward–triggered discharges and different behaviors. This result can be well understood when considering that dACC can signal a given behavioral strategy when its activity lies close to a given prototypical state. Hence, this interpretation can be consistent with a report of increased spike count variability (and hence, of absence of defined state of activity) in dACC during periods of behavioral uncertainty [56]. It can also be related to the sudden reorganization of dACC activity in a new “rule-encoding network state” when animals switch to a new rule [57]. Within this framework, first reward feedback triggers specific dACC activity patterns [58] that shape the response of downstream areas such that the appropriate decision (here, switching to repetition) is made. Deviation from these “prototypical patterns” would lead to a slower behavioral response. In addition, if the deviation of dACC discharges from their usual pattern were triggered by increased uncertainty or difficulty, slowing the behavioral response may prevent incorrect choices (as suggested by the similar error rates between trials with fast versus slow responses).

Interestingly, these results also suggest that the information transmitted to downstream areas cannot be mapped onto a mere intensity value (i.e., a single dimension), such as the magnitude of the required cognitive control, as in the case of the integrator model. Rather, the deviation from a prototypical pattern, which relates to behavioral modulation, appeared to occur in many different ways (through either an increase or a decrease of spike count, or through spike timing deviations within the heterogeneous temporal patterns of dACC neurons). This hints to the transmission of a high-dimensional representation by dACC, possibly linked to the embedding of the cognitive control signal into a specific context, or behavioral strategy [1,29,30]. One limitation of our study is that we only characterized the dimensionality of the representation transmitted by dACC through the large differentiation, at the population level, between measures based on firing rate and measures based on (absolute) deviation from prototype. A full evaluation of this dimensionality will need future studies to evaluate the space of neuronal variability and its relation to behavioral variability in each single neuron.

Importantly, beyond the deviations of dACC spike trains from prototypical spike count, our findings indicate that deviations from prototypical temporal patterns were predictive of the monkeys’ upcoming response time. This was consistent and significant in both monkeys. Furthermore, compared to the prediction based on spike count deviations, the prediction power of adapted temporal sensitivity was either equivalent (monkey P) or significantly stronger (for monkey M, which showed the most reliable relation between neural activity and behavior). This strongly suggests that the temporal patterning of single unit activity is not an epiphenomenon irrelevant to downstream network dynamics.

Interestingly, dACC differs from other decision-making related areas such as middle temporal (MT) or orbitofrontal cortex (OFC) regarding the nature of the relation between neuronal variability and future response time variability. Indeed, in MT and OFC, the firing rate of specific neuronal populations predicts behavioral modulation [59,60]. In addition, evidence suggests that neurons in MT are decoded through integration, a process that could be reflected in LIP (lateral intraparietal cortex) activity [61] and that appears to have one-dimensional dynamics [62].

Altogether, our results appear hard to reconcile with the hypothesis of a decoding of post-feedback dACC activity by a neural integrator. Other types of decoders could be compatible with both an increase in information through spatiotemporal coincidences and a correlation of deviation from prototypical temporal patterns to behavior. For instance, as we illustrate in Fig 1c, a recurrently connected neuronal population, which maintains memory through a high-activity state, can be modulated by the temporal structure of its input [32]. Alternatively, a downstream network maintaining a memory through repetitions of sequential activations of NMDA-connected neurons would also be sensitive to spatiotemporal patterns [63].

Our findings, therefore, call for a better understanding of how models of short-term memory and decision-making could reliably be modulated by a temporal input at the timescale of hundreds of ms.

## Materials and Methods

### Electrophysiological Recordings

Two male rhesus monkeys were implanted with a head-restraining device, and neuronal activity was recorded by one to four epoxy-coated tungsten electrodes (horizontal separation: 150 μm) placed in guide tubes and independently advanced in the dorsal bank of the rostral region of the cingulate sulcus. Recording sites were confirmed through anatomical MRI and histology [11,29]. Extracellular activity was sampled at 13 kHz and unitary discharges were identified using online spike sorting based on template matching (MSD, AlphaOmega). All experimental procedures were in agreement with European, national, and local directives on animal research.

### Analyzed Units

For monkey P, all recorded units were used. For monkey M, only units showing a significant response to at least one event (either error, or first reward, or repetition reward, fixation breaks) were used (TEST 1 in [29]). The mean and standard deviation of the baseline firing rate (taken from -600 to -200 ms before feedback onset) were computed. Units with a change of firing rate of magnitude higher than 5 standard deviation of the baseline within more than six 10 ms bins between +60 and +800 ms of at least one event were selected. Note that this test cannot favor temporal coding in any way.

### Problem Solving Task

Monkeys had to find, by trial-and-error, the rewarded target among 4 targets presented on a touch screen (Fig 1a). To begin a trial, the animal had to touch a central item (“lever”), which triggered the appearance of a fixation point. After 2 s of gaze fixation, the four targets appeared simultaneously. At fixation point offset, the animal had to select a target by making a saccade toward it, fixate it for 0.5 s, and touch it following a “go” signal (i.e., all targets bright). All targets dimed at the touch, and switched off after 0.6 s. Reward (fruit juice) was delivered if the correct target was selected, otherwise no reward occurred. Throughout this article, we define a “trial” as the period of time between the touch of the lever and 1 s after the reception of a feedback (either error, or first reward, or repetition reward). In addition, we call “task epoch” the time interval between 1 ms and 1 s after the reception of a given feedback. After feedback, a time break of 2 s was imposed before starting a new trial. Any break in gaze fixation or touch within a trial led to resuming the sequence at the lever touch. Note that we did not consider that this started a new trial.

In case of an incorrect choice, the animal could select another target in the following trial, and so on until the discovery of the rewarded target (i.e., exploration). The correct target remained the same in the following trials, allowing the animal to exploit the rewarded action (i.e., repetition). We define a “problem” as the block of trials that are associated with one rewarded target location and that terminate with a “signal to change”. This signal to change was a flashing signal indicated the end of repetition and the beginning of a new problem (the new rewarded target had a 90% probability to be different from the target rewarded in the preceding problem). In 90% of problems, the repetition period lasted three trials after the first reward, whereas in 10% of problems, 7–11 repetitions could occur. Repetition trials beyond the third one were excluded from analysis to avoid possible surprise effects. At the time of recordings, the task was well known: monkeys omitted to repeat the correct touch in one of the trials following the discovery of the rewarded target in only around 1% of problems. Then, both the incorrect touch and the following trials were discarded from analysis, but previous trials were kept. As previously reported [41], monkeys might be able to infer the rewarded target after three non-redundant errors, i.e., the third error would systematically trigger a switch to repetition. Therefore, only first and second erroneous touches as well as first rewards preceded by less than three errors were included in the analysis. For the repetition period, we selected all correct trials that followed a search with up to three preceding search errors.

### Decoding dACC Neuronal Activity through Spike-Train Metrics Analysis

We assessed to what extent single-trial spike trains encoded enough information to discriminate between the following types of post-feedback task epochs: (a) after first reward versus after second, third, and fourth rewards, i.e., “first reward discrimination;” (b) after no reward versus after second, third, and fourth rewards, i.e., “error discrimination.”

To compare different decoding schemes (i.e., spike-count and timing-sensitive decoders), the spike-train metrics framework [13,35] was considered. Neuronal decoding relied on the assessment of the Victor and Purpura distance d_q,k_(s,s’) between two spike trains (s,s’), both within and between task epochs. One spike train s contained the spikes of one (Fig 2a) or two (Fig 2b) neurons during one task-epoch of a given trial. The distance d_q,k_ (also named “dissimilarity” throughout the article) depended on two parameters, namely, the spike timing sensitivity q and, in the case of multiunit activity, the degree of distinction *k* between different neurons (Fig 2). Algorithmically, the distance d_q,k_ between two spike trains was computed as the minimal cost to transform the first spike train into the second one, using three possible operations:

1. adding or removing a spike, for a cost (D_max_/2) = 1;
2. changing the timing of a spike by an amount dt, for a cost (q dt) ≤ D_max_, where D_max_ = 2 is the maximum cost corresponding to removing a spike and re-inserting it at the right time;
3. changing the identity of the neuron which fired the spike, for a cost *k*.

Each spike train s was classified as belonging to the task epoch E that contained the most similar neuronal responses to s. The similarity between s and all discharges produced during E was quantified by computing a global dissimilarity to the ensemble of spike trains in task epoch E. The results presented in the main body were obtained by taking this global dissimilarity as the median of the pairwise distances between s and any other spike train of E (i.e., *median*(*d*_*q*_(*s*, *s'*))_s'∊E, s' ≠ s_). Comparable results were found by biasing the global dissimilarity measure towards small spike train distances, i.e., by “ignoring” more the very dissimilar spike trains during classification (“nearest-neighbor” classification, S5 Fig).

The accuracy of the classification process was measured as the mutual information between the actual probabilities of dACC spike trains to belong to each task epoch and the probabilities predicted by the clustering method. More precisely, if we define a confusion matrix N, whose entries N(i,j) are the number of spike trains belonging to task epoch i and classified in task epoch j, the raw information was $\frac{1}{\sum_{I,J}N_{I,J}}\sum_{i,j}N_{i,j}\;ln\left(\frac{N_{i,j}\;\sum_{I,J}N_{I,J}}{\left(\sum_{k}N_{i,k}\right)\left(\sum_{l}N_{l,j}\right)}\right)$. This measure accounts for the differences in number of trials between task epochs, it tends toward zero for chance-like predictions (for large data samples), and its maximum (for perfect prediction) depends on the relative proportion of trials between task epochs. As the number of trials was neuron dependent, each information value was normalized with respect to its maximum value. Full details on the use of spike-train metrics are in S1 Text.

To test whether classification was above chance, trials were randomly permuted between task epochs, and two groups were recreated (with the same number of trials as the original task epoch clusters). The information content associated to the shuffled groups was then computed. The process was repeated 1,000 times, leading for each q or [q,k], to 1,000 values of information under the null hypothesis that the discrimination between groups is due to random similarities between any two spike trains.

The information analysis was done on increasing time windows, starting 1 ms after the onset of the feedback (to avoid pump-driven artifacts). The first window lasted until 50 ms post-feedback, and was incrementally increased to 600 ms by 50 ms steps, and then up to 1 s by 100 ms steps. The higher resolution for smaller windows allowed the time course of the fast initial transient to be evaluated. We computed the maximum (over q or [q,k]) number N_w_ of consecutive windows for which the information was strictly larger than the 95th percentile of the 1,000 sets of permuted data. The same process was repeated for each set of permuted data, relative to the remaining 999 permuted sets. A neuron (or a pair) was considered as significant if N_w_ was strictly larger in the actual data than in 95% of permuted data. This process did not favor a given q or k, and could select neurons/pairs of neurons with different information time courses. Also, it allowed us to exclude neurons with very unreliable activity, which would act as “noise” during the subsequent analyses.

The sample information bias was empirically estimated (for each q or [q,k] and each analysis window) as the mean information in the 1,000 permuted datasets [64], and was subtracted from the information in the original data (slightly negative corrected information were clipped at 0).

Note that, throughout the paper, the term “information” refers to a bias-corrected and normalized-to-maximum value.

After bias correction, for each q or [q,k] and for each significant neuron, the temporal evolution of information values was summarized by taking the mean information over 10 analysis windows of increasing lengths (ending from 100 ms to 1 s post-feedback onset, by steps of 100 ms, favoring neither early nor late information). We refer to this quantity as “time-averaged information” in the article, <I>_t_. Computing the time-averaged information is equivalent to averaging over delays before a decision is made by the animal.

Finally, a non-parametric Friedman ANOVA was used to compare the time-averaged bias-corrected normalized information as a function of different q or [q,k], with Tukey's honestly significant difference criterion correction for multiple comparisons.

Note that our procedure is more powerful than simply counting the number of neurons that would reach a certain significance level separately for each q or [q,k]. Indeed, the latter method cannot be sensitive to differences in classification ability between two parameter sets that reach a certain significance level, and it is restricted to the use of large enough *p*-values (which can be evaluated with a reasonable number of permutations).

### Shuffling of Spike Times

We used spike-time shuffling to investigate to what extent random samples from a time-varying trial-averaged rate density (as in “Poisson” neurons with time-varying rate) could underlie the advantage of the temporal structure for decoding [35]. For each cell and each task epoch separately, we grouped all spikes emitted in the interval [0.001, 1] s post-feedback and randomly assigned each of them to a trial (repeated 1,000 times, Fig 5a). This procedure is equivalent to drawing the number of spikes in each trial from a Binomial distribution with parameters *n* = *N*_*spikes*_ and $p=\frac{1}{N_{trials}}$, which—following the common approximation—is close to a Poisson variable. Indeed, *p* was rather small (the trial number was usually big: 25th quantiles were 14.25 and 51.25 for first reward and repetition respectively); and the total number of spikes *n* was big (25th quantiles were 53.75 and 175 for first reward and repetition, respectively). Under the Poisson approximation, spike counts restricted to sub-analysis windows are also Poisson (Raikov's theorem). This allowed us to build the spike-shuffled data for smaller analysis windows by simple pruning of the 1,000 shuffled data of the largest window.

We used a second shuffling procedure to test to what extent information transmission could be determined by time-varying firing rates and spike-count variability as in the original data (Fig 5d). In contrast to the previous shuffling method, this procedure considered that time-varying firing rate was modulated by a multiplicative factor. This factor constrained the spike-count variability to fit the original data and it was specific to each trial and time independent. This shuffling procedure not only conserved the PETH but also the number of spikes present in each trial. To do so, for each cell, each task epoch, each analysis window, and all 1,000 shufflings independently, we randomly permuted all available spikes before reassigning to each trial the exact same number of spikes as in original data (without replacement).

Because both shuffling methods produced spike-shuffled data with the same number of trials as in the original data, the finite-sample information bias should be similar in both cases and should cancel when looking at the information difference, which was the relevant quantity. The bias was therefore not re-evaluated for this analysis.

### Behavioral Response Time Analysis

The response time was defined as the time between the “go” signal (for the hand movement) following the first reward, and the subsequent target touch. For this analysis, only neurons with significant first reward classification and with at least five available trials were used (or subgroups of this ensemble, see S12 Fig).

For a given neuron, the dissimilarity between a recorded spike train s and the prototypical spike train s_prototype_ emitted at first reward was estimated as the median of all pairwise distances d*_q_(s,s’), where s’ corresponds to all the recorded first reward spike trains that are different from s. We defined a new metrics d*_q_ by dividing the Victor and Purpura distance d_q_ by either the number of coincident spike pairs between s and s’, or 1 if no spikes were coincident. Note that we define two spikes to be coincident when their dissimilarity was smaller than D_max_ = 2 (see Fig 2a). For q > 0, the distance d*_q_ quantified the mean jitter of coincident spikes, plus a cost for unmatched spikes. For q = 0, d*_q_ estimated the normalized absolute spike-count difference between the two spike trains. Importantly, d*_q_ did not scale with the number of emitted spikes: a trial with too little spikes, as well as a trial with many spikes at inaccurate times, will have a large normalized distance to the prototype. We further discuss the rationale for designing d*_q_ in S4 Text.

Let $\tilde{r}$ denote the median value of observed response times, *T*_+_ be the set of first reward trials followed by a response time larger than $\tilde{r}$, and *T*_-_ the set of trials followed by a response time lower than $\tilde{r}$. For each spike train s, we calculated the dissimilarity between s and prototypical first reward activity (i.e. $median{\left(d_{q}^{*}(s,s\prime )\right)}_{s\prime \in 1^{st}\;reward,s\prime \neq s}$, similar to the spike train classification analysis). We then defined ${\bar{D}}_{T+}$(${\bar{D}}_{T-}$) as the mean over all *s*∊*T*_+_(*T*_-_) of the dissimilarity between s and prototypical first reward activity. We finally computed the overall difference of deviation from the prototypical discharge at first reward as $\bar{D}={\bar{D}}_{T+}-{\bar{D}}_{T-}$. $\bar{D}$ was computed for multiple time window lengths: from 100 ms to 1 s post-feedback time, by increments of 100 ms. Finally, a bias score $b=\underset{\begin{array}{c} positive\;bias\\ windows \end{array}}{\sum }-{\text{log}}_{10}\left(p_{signed-rank}\left(\bar{D}\right)\right)+\underset{\begin{array}{c} negative\;bias\\ windows \end{array}}{\sum }{\text{log}}_{10}\left(p_{signed-rank}\left(\bar{D}\right)\right)$ was computed, where the set of “positive bias windows” contained those analysis windows for which the sum of ranks for positive values was larger than the sum of ranks for negative values. Similarly, the “negative bias windows” were those with a sum of ranks for negative values larger than the sum of ranks for positive values. A positive (negative) bias in a given window would cause a corresponding increase (decrease) in b.

To assess the significance of the bias score b, 1,000 surrogate datasets, in which the difference between high and low response time groups was eliminated, were compared to the real data. For each surrogate, and independently for each neuron, the sign of all $\bar{D}$ values (for all analysis windows) had a 0.5 probability to be changed. The *p*-value was computed as the proportion of surrogate datasets leading to higher or equal |*b*|as the real data.

The temporal sensitivity q leading to best first reward discrimination in the population and q = 0 were compared (signed-rank test). To do so, we computed the mean values of $\bar{D}$ over analysis windows ending from 100 ms to 1 s post-feedback time, by increments of 100 ms. Similar results were found when assessing the optimal q value by using either the original Victor and Purpura distance d_q_ (main text), or the normalized distance d*_q_ (S12 Fig and S4 Text).

A similar analysis was done to test whether firing rates could also relate to response time. To do so, $\bar{D}$ was replaced by the difference in mean firing rate between high and low response time trials, ${\bar{D}}_{rate}$.

### Statistical Tests

Table 1 summarizes an additional set of employed statistical measures. The latter were often non-normal; therefore, non-parametric tests were considered (*p* ≤ 0.05 was considered as statistically significant): (1) correlations were assessed with Spearman coefficient with a permutation test (or big sample approximation); (2) distributions were compared with the 2-sided Kolmogorov-Smirnov test; (3) central tendencies were compared between 2 unpaired (resp. paired) distributions with the 2-sided ranked-sum (signed-rank) test; (4) deviation of distributions from 0-centered-symmetry was also tested with the 2-sided signed-rank test.

When testing pairs of units, one limitation was that some pairs happened to share a neuron, and hence were correlated (in particular if non-shared neurons were discharging significantly less than the shared one). This was problematic for analyzing the optimal temporal sensitivity, which is not a parameter accounting for the interaction between neurons, and which can be impacted more by the neuron which fires the most. We therefore verified that the significance of the advantage of the temporal sensitivity during paired decoding could be reached without overlapping pairs (positivity of $\underset{k}{\text{max}}\left({\langle I\left(q=10s^{-1}\right)\rangle }_{t}\right)-\underset{k}{\text{max}}\left({\langle I\left(q=0s^{-1}\right)\rangle }_{t}\right)$, signed-rank test, *p* ≤ 0.05 in 1,000/1,000 random down-samplings to non-overlapping pairs). Note that, in contrast, interaction parameters such as the information gain or k_opt_ are truly pair specific, implying that it was reasonable to keep overlapping pairs for the analysis.

Note that although most statistical tests presented in the main results were carried out by pooling data from both monkeys, consistent trends were observed for both individuals.

## Supporting Information

S1 Fig Related to Fig 4. Robustness of spike-timing information in both monkeys. The improvement of decoding trough spike-timing sensitivity was robust in both monkeys. The left part of the figure describes the result of the discrimination between first reward and repetition, and the right part describes errors versus repetition discrimination. **(a,b)** Time course of the mean information over neurons, for different temporal sensitivities of the decoder (q) as indicated on the color scale, for monkey M and P respectively. **(c)** Difference of time-averaged information <I>_t_ (see Materials and Methods and main text Table 1) between temporal decoding (q_opt_ ≈ 10 s^-1^) and spike-count decoding (q = 0 s^-1^). The *p*-value of a signed-rank test indicates that in both monkeys individually, temporal sensitivity induced a robust increase of information (all p_s_ < 0.018).
(PDF)

S2 FigRelated to Figs 4 and 8. Using a small temporal sensitivity (compatible with decoding by an imperfect integrator) leads to identical conclusions to using q = 0/s (perfect integration) in single units.We test: (i) q = 0.5s^-1^, approximately equivalent to an exponential leak time-scale τ = (1/q) = 2s (main text Figs 1 and 2a and S1 Text). This is the minimal time-scale for a downstream leaky neuronal integrator which has to hold in memory the behavioral adaptation signals (and/or the behavioral strategy signals) for up to 3–6 s as required during the task (in case of fixation break). (ii) q = 1s^-1^, approximately equivalent to an exponentially decaying time-scale τ = 1s, as a more stringent test. **(a,b,c)** Classifying spike trains: first reward versus repetition **(a)**, errors versus repetition **(b)**, errors versus first reward **(c)**. We used neurons reaching significant classification with any q-value (including q = 0.5 and 1s^-1^, permutation test, Methods), leading to only one more significant neuron compared to main text (for errors versus repetition classification, monkey P). Left: time course of the mean information over neurons. Right: results of post-hoc comparisons of the time-averaged information <I>_t_ after a Friedman anova, using the Tukey's honestly significant criterion correction. Q-values with significantly smaller performance than q_opt_ are marked by a star. In all considered cases, both q = 0.5s^-1^ and q = 1s^-1^ were leading to significantly smaller <I>_t_ than q_opt_. In both monkeys individually, q = 0.5s^-1^ and q = 1s^-1^ had (at least qualitatively) lower average rank than q = 10s^-1^ and q = 5s^-1^. The Friedman test was restricted to q ≤ 40s^-1^, focusing on q-values for which classification was not too noisy. Finally, note that the slight differences in the rankings of q-values between the mean-information time course and Friedman anova are due to the fact that the mean is more sensitive to outliers with large values, while the average rank is determined by the consistency (over neurons) of the within-neuron rankings of <I>_t_ between different q-values. **(d)** Comparing ${\langle \bar{D}\rangle }_{t}$: the time-averaged index of behavioral prediction through deviation from prototypical first reward spike train, between q_opt_ ≈ 10 s^-1^ and several lower temporal sensitivities. The average was taken over analysis windows ending between 0.1 s and 1 s with steps of 0.1 s. The data shown is the difference between ${\langle \bar{D}\left(q_{opt}\right)\rangle }_{t}$ and ${\langle \bar{D}\left(q<q_{opt}\right)\rangle }_{t}$. Note that for monkey M, q = 0s^-1^, q = 0.5s^-1^, and q = 1s^-1^ lead to significantly smaller ${\langle \bar{D}\rangle }_{t}$ compared to q_opt_, while a statistical equivalence was seen in monkey P (signed-rank test).(PDF)

S3 Fig Related to Fig 4. Advantage of spike-timing-sensitive decoding over spike-count decoding for very informative neurons. Spike-timing-sensitive decoding was also beneficial for very informative single neurons. We computed the maximum time-averaged information I_max_ for significant units (over q). Then, we used a k-means algorithm (with two groups) to separate populations with high versus low I_max_. Results in this figure are for the high I_max_ neurons. **(a,b)** show the time course of the mean information (over neurons) for first reward (left) and errors (right) discrimination, as a function of timing sensitivity q, separately for the two monkeys. The inset in **(b, right)** shows the difference of time-averaged information <I>_t_ between q = 5s^-1^ (found optimal for monkey P over all significant units, for errors discrimination) and q = 0s^-1^. The p-value of a signed-rank test is indicated. **(c)** boxplots of the corresponding distributions of difference in <I>_t_ between q = 10 and q = 0 s^-1^. *P*-values of signed rank tests are indicated. Note that the notches indicate a confidence interval on the median, which may extend further away than the 25th or 75th quantiles, resulting in an inversion of the boxplot.
(PDF)

S4 Fig Related to Fig 4. The optimal decoding temporal sensitivity appeared higher for neurons firing more during behavioral adaptation. We took advantage of the fact that the Victor and Purpura metrics scales with the number of spikes (see S1 Text and S4 Text) to compare the apparent firing reliability between task-epochs. For a given neuron, the q_opt_ computed with this metric is expected to mostly reflect the spike-timing reliability of the task-epoch with more spikes, which spike trains are harder to classify. Indeed, within spike trains of this task-epoch, a small dissimilarity d can only be reached (and therefore correct classification can only happen) if the decoder detects a very small dissimilarity per spike and therefore a sufficiently small summed dissimilarity over all spikes. Hence, we compared groups of neurons firing preferentially in different task-epochs. *Left*: first reward versus repetition discrimination; *right*: errors versus repetition discrimination. **(a)** Difference of mean spike count in a [0.001,1]s post-feedback window between behavioral adaptation and repetition epochs. **(b, Left)** Boxplots of the distributions of q_opt_ values for cells discharging preferentially during behavioral adaptation versus repetition (the notches indicate an approximate confidence interval on the median, which may extend beyond the quartiles). *P*-values of ranked sum tests comparing medians are shown. The higher q_opts_ for neurons firing more during behavioral adaptation could reflect a real higher reliability of firing, or the fact that the temporal reference used for the analysis was more reliably locked to the monkey's internal reference during behavioral adaptation epochs. Indeed, monkeys could anticipate the outcome before the feedback was explicitly given during repetition, potentially leading to a trial-specific advance of dACC firing compared to feedback time. **(b, Right)** Boxplots of the distributions of time-averaged information <I>_t_ for q = 0s^-1^. *P*-values of ranked sum tests are shown. The absence of significant difference suggests that the difference in q_opt_ (left) reflects a difference in spike-timing reliability rather than a difference in spike-count reliability between the groups. **(c)** Detailed analysis about the optimal temporal sensitivity q_opt_ for decoding cognitive-control signals during feedbacks of the task which should trigger behavioral adaptation (first reward or errors). We focus on neurons discharging more during first reward for first reward versus repetition discrimination and on neurons discharging more during errors for errors versus repetition discrimination. In addition, for errors versus repetition discrimination, we focus on cognitive control coding and exclude putative 'physical reward' coding by only selecting neurons that were significant for both errors versus repetition and first reward versus repetition (*n* = 32 from monkey M, *n* = 27 from monkey P). **(c, Left)** Difference of time-averaged information <I>_t_ between q = 10s^-1^ and q = 5s^-1^, and between q = 5s^-1^ and q = 1s^-1^; the *p*-value of a signed-rank test for the distribution of the difference values around 0 is shown. For first reward versus repetition, information increased significantly in both cases. For errors versus repetition discrimination, information increases significantly between q = 5s^-1^ and q = 1s^-1^ and a statistical equivalence was seen between q = 10s^-1^ and q = 5s^-1^. Our results show that a biologically plausible range of temporal sensitivities (equivalent to decaying time-constants of 100 ms for first reward, and 100/200 ms for errors) improves information transmission. **(c, Right)** Distribution of optimal temporal sensitivities (here, including data at q = 0.5s^-1^ and q = 1s^-1^) showing that for both discriminations and for both monkeys independently, the median q_opt_ was 10s^-1^. For errors versus repetition, the pooled distribution over monkeys (concerning a subset of neurons compared to **(b)**) is also shown.
(PDF)

S5 Fig Related to Fig 4. Information gain through temporal sensitivity using a classification biased toward closer neighbors instead of the unbiased classification used in main text. Information gain through temporal sensitivity was also observed when the classification of spike trains was biased toward smaller dissimilarities rather than determined by the median dissimilarity to spike trains of a task-epoch (see S1 Text, section: classification). Results in this figure are for the neurons with significant discrimination ability (permutation test, see main text Materials and Methods); note that the number of significant units is smaller than with the classification method of main text. **(a,b)** show the time course of the mean information (over neurons) for first reward (*left*) and errors (*right*) discrimination, as a function of timing sensitivity q, separately for the two monkeys. **(c)** Results of the post-hoc comparisons (with Tukey's honestly significant criterion correction for multiple comparisons) of a Friedman ANOVA comparing the time-averaged information <I>_t_ between temporal sensitivities. Note that the slight differences in the rankings of q-values between **(a,b)** and **(c)** are due to the fact that the mean over neurons is more sensitive to outliers with high values, while the average rank is determined by the consistency (over the population of single units) of the within-neuron rankings of <I>_t_ between different q-values.
(PDF)

S6 Fig Related to Fig 4 and S3 Text. Decoding trials without eye-movements (monkey M). We observed temporal coding when using first reward versus repetition trials without detected eye-movements (for analysis windows <650ms). See S3 Text for methods. A small dot indicates *p* < 0.1, one star: *p* < 0.05, two stars: *p* < 0.01 for signed-rank tests. Error bars are standard error of the mean/median as in main text. **(a,b,c)** Behavior for 28 sessions (during which we recorded significant first reward versus repetition). **(d,e,f,g,h,i,j)** Decoding; **(d,e,i)** are related to the putative influence of motor feedback activity and **(f,g,j)** to the putative influence of premotor activity. 38 neurons were available for analysis windows <425 ms at least; for longer windows some neurons were excluded because no trials free of saccades were available. **(a)** Cumulative distribution function of first eye-movement latency following the fixation period. 95% confidence interval using Greenwood's formula. **(b)** as **(a)** but restricted to post-reward first eye-movement latency. **(c)** Distributions of median response times at the trial following first reward depending on the first eye-movement latency after the fixation period leading to first reward. **(d)** *Left*: Neuron-averaged information, while only trials without eye-movements detected yet were included. *P*-values compare between q = 10s^-1^ and q = 0s^-1^. *Right*: Neuron-averaged information for random downsamples (from all data) to the trial numbers of **(d Left)**. The downsampling aims at excluding a possible effect of trial number when comparing data without (left) and with (right) saccades. For each neuron, the mean information among 1,000 downsamples was taken (taking the median gives similar results). *P*-values compare between q = 10s^-1^ and q = 0s^-1^. Note that the smoother aspect of the curves compared to the left graph likely results from the presence of an additional downsamplings-averaging in the right graph. Note also that, until plateau is reached (≈600 ms post-feedback), there were no robust differences in spike-count based information between eye-movement free and resampled data (signed-rank test on time-averaged information between 0 and 600 ms, or 300 and 600 ms, all *p*-values> 0.16). **(e)** Median difference of information increase thanks to temporal structure: [I(q = 10s^-1^)—I(q = 0s^-1^)], between eye-movement-removed (d Left) and randomly downsampled data (d Right). Negative values indicate a smaller timing-sensitivity-related improvement in decoding for eye-movement-free data. **(f)** Conventions as in **(d)**. *Left*: Only trials for which first eye-movement occurred later than ([analysis window end] + 300 ms) were included. Due to limitations in trial number, for analyses windows longer than 700 ms, all trials with first eye-movement latency ≥1 s post-reward were included. *Right*: data randomly downsampled to the trial number of (f Left). **(g)** Difference of information increase thanks to temporal structure: [I(q = 10^−1^)—I(q = 0s^-1^)], between eye-movement-removed (f Left) and randomly downsampled data (f Right). **(h)** Neuron-averaged information among all 38 available neurons, all trials included. **(i)** Difference of information increase thanks to temporal structure: [I(q = 10s^-1^)—I(q = 0s^-1^)], between eye-movement-removed as in (d Left), and total data. **(j)** Difference of information increase thanks to temporal structure: [I(q = 10s^-1^)-I(q = 0s^-1^)], between eye-movement-removed as in (f Left), and total data.
(PDF)

S7 Fig Related to Fig 4. Decoding the identity of the adapted behavioral strategy (exploration or switch). The data suggest an advantage of spike-timing sensitivity for decoding the identity of the adapted behavioral strategy (exploration or switch). **(a,b,c)** Single unit decoding between errors and first reward spike trains, for all neurons with significant errors versus first reward classification. **(a,b)** Time course of the mean information for different temporal sensitivities as indicated in the colorbar, for monkey M **(a)** and monkey P **(b)**. **(c)** Mean rank (+/-95% confidence interval) of post hoc comparisons (using Tukey's honestly significant criterion correction for multiple comparisons) of a Friedman ANOVA comparing the time-averaged information <I>_t_. The average was taken over analysis windows ending between 0.1 s and 1 s with steps of 0.1 s. Data from both monkeys were pooled. **(d,e,f)** Decoding performance for errors versus first reward classification, restricted to neurons that were significant for both errors versus first reward classification and first reward versus repetition classification. The discharge of these neurons cannot therefore be merely related to the reward quantity received by the monkey, instead they appear correlated with the nature of the adapted behavioral strategy. **(d)** Time course of mean information for different temporal sensitivities as indicated in the colorbar (data from both monkeys pooled). **(e)** Mean rank (+/-95% confidence interval) of post hoc comparisons (using Tukey's honestly significant criterion correction for multiple comparison) of a Friedman ANOVA comparing the time-averaged information <I>_t_. Note that the slight differences in the rankings of q-values between the mean information and the Friedman anova graphs are due to the fact that the mean (up) is more sensitive to outliers with large values, while the average rank (down) is determined by the consistency (over neurons) of the within-neuron rankings of <I>_t_ between different q-values. These outliers are, for instance, visible in monkey P in **(f)**. Note that some of these outliers might be due to noise (e.g., the lower outlier in monkey P in (f) had the smallest number of trials, and fewer trials were available for monkey P, see S1 Table). **(f)** Boxplots showing the distribution of the difference <I(q = 10s^-1^)>_t_−<I(q = 0s^-1^)>_t_ for the two monkeys separately. Note that q = 10s^-1^ ~ q_opt_. A signed rank test was significant in both monkeys individually, showing evidence for spike-timing-related information improvement in both monkeys.
(PDF)

S8 Fig Related to Fig 5. Robustness of the link between spiking statistics and information transmission. **(a)** The changes in information induced by performing shuffle 1 (preserving the time-dependent rate) were consistent over monkeys and were following the time course of the fano factors (see main text Fig 5). For long analyses windows, original data were less reliable than their spike-shuffled counterparts, while this effect was inverted for short analysis windows. The curves are the mean +/- standard error (ste, among all significant neurons for first reward versus repetition classification) of the difference between the information in the original data and the median information of the corresponding shuffled datasets. We show q = 0 (spike-count decoding, black) and q = 10s^-1^ ≈ q_opt_ (blue). **(b)** Same conventions as in **(a)**. The change in information induced by shuffling spikes according to shuffle 2 (preserving both time-dependent rate and spike count variability, see main text Fig 5) were consistent over monkeys. Original data had higher information than their spike-shuffled counterparts. **(c)** The distribution of difference of time-averaged information (<I(q = 10s^-1^ ≈ q_opt_)>_t_) between original data and the median for the corresponding datasets created by shuffle 2 was significantly positively biased for both monkeys (left) and for both the neurons firing more during first reward and the neurons firing more during repetition (signed-rank tests, all p_s_ < 0.036). Note that q_opt_ is unambiguously 10s^-1^ for neurons firing more during first reward (for these neurons q = 5s^-1^ and q = 15s^-1^ perform very similarly for original data decoding, see also S4c Fig). The distributions were not different between monkeys or between firing preference (ranked-sum tests, all p_s_ > 0.38). **(d)** We show the means (over neurons) of (i) the information in original data, and of (ii) the median information of the corresponding shuffled datasets. For all q values, we observed higher information for the original data as compared to their shuffle 2 counterparts. The size of the effect increased for higher q values. Spike correlations were probably stronger at smaller time-scales. **(e)** The proportion of neurons for which shuffle 2 led to a significant decrease in <I(q = 10s^-1^)>_t_ (more than 95% of shuffled datasets with smaller <I(q = 10s^-1^)>_t_ than original, left), was higher than the proportion of neurons with a significant increase (more than 95% of shuffled datasets with larger <I(q = 10s^-1^)>_t_ than original, right). Proportions were compared using the Fisher's Exact Probability Test with mid-p correction (*p* = 4.0 10^−5^). Both of these proportions were larger than chance (5%): binomial test, all p_s_ < 10^−3^.
(PDF)

S9 Fig Related to Fig 6. Positively biased information gains correlating with the information imbalance between the isolated single units for pairs of neurons with significant information. Paired spatial decoding led to increases in the amount of information despite imbalances in the discriminative power of single units. In this figure, only pairs with significant classification (permutation test) were included. **(a)** Discrimination between first reward and repetition task-epochs. The central plot shows the correlation between the information gain (obtained when decoding a neuron pair versus the pair’s most informative single unit, see main text Table 1) and the degree of information imbalance between the two units of a pair. A permutation test was used to determine the significance of the correlation (*p* < 0.001). The histograms at the top and right show the two marginal distributions. A signed-rank test was used to measure the significance of the bias towards an increase in the amount of information (i.e. positive gains, *p* < 0.001). **(b)** Same as **(a)** but for the discrimination between error and repetition task-epochs.
(PDF)

S10 Fig Related to Fig 6. Consistence of the modulation of information in neuron pairs by the temporal sensitivity (q) and the between-unit distinction degree (k) in the two monkeys. **(a)** Time course of the mean information among neuron pairs with significant discrimination. Different sheets with different green shadings are different analysis window durations, as indicated on the color scale on the right. For both monkeys and consistently over analysis windows, information increased with adapted temporal sensitivity compared to spike count decoding (q = 0s^-1^), and on average the information was larger for intermediate-to-large values of the discrimination between neurons (k). In this figure, only pairs with significant classification (permutation test) were included, as in the bottom part of main text Fig 6. **(b)** *Left*: maximum (over time-windows) mean (over pairs) information for first reward and error discrimination, as a function of timing sensitivity q and between-unit discrimination degree k. Information was maximized over analysis windows ending in [0.05,0.6]s, steps of 50 ms, and in [0.7,1]s, steps of 100ms. *Right*: comparison of (q, k) for the time-averaged information <I>_t_ of pairs of neurons. The plots display the results of post hoc comparisons (using Tukey's honestly significant criterion correction after a Friedman ANOVA) between <I>_t_ (computed with analysis windows ending in [0.1,1]s, steps 100 ms). The red dot marks the (q,k)_opt_ value leading to the higher rank; black dots mark (q,k) values that are not significantly different from (q,k)_opt_, and green dots mark (q,k) values that have significantly smaller ranks than (q,k)_opt_.
(PDF)

S11 Fig Related to Fig 8. Modulation of behavioral response times following first reward trials. The analysis was restricted to the trials that were used for Fig 8 in the main text. **(a)** Modulation of the release time following first reward by the identity of the rewarding target and by the number of errors made preceding the first reward. The release time was defined as the time between the post-first-reward go signal for target touch (by the hand) and the release of the central lever button. Groups were compared with a non-parametric Kruskal-Wallis test (see *p*-value at the top-left). Post-hoc comparisons were conducted using Tukey's honestly significant criterion correction. Note that for all rewarding targets, the release movement occurred at the same place: on the central lever button. The release time modulation is therefore not likely to reflect motor constraints. **(b)** Modulation of the response time following first reward by the identity of the target and by the number of errors, conventions as in a). The response time was defined as the time between the post-first-reward go signal for target touch and the following target touch. The modulation of the response time was strikingly similar to the modulation of the release time (which, as argued above, is very unlikely to reflect motor constraints). In addition, note that while the two monkeys were in the same apparatus, they modulated their response time differently for the different targets. Finally, the target modulation of response time could interact with the modulation by the number of preceding errors. Altogether, the results argue against a purely motor cause for response time modulation, and rather point toward a spatial bias of cognitive processes. **(c)** Boxplots for the difference of response time between the trial following first reward (the first repetition, or, in rare cases, a mistake) and the trial that ended with the first reward, i.e., last exploration. The *p*-value of a signed rank test for a bias of the distribution toward either positive or negative values is indicated. The green line indicates a 0 difference. For clarity, outliers are omitted. The response time increased on the trial following first reward when the preceding exploration period was longer than one attempt.
(PDF)

S12 FigRelated to Fig 8. Consistency of the relation between neural activity and behavior in different subgroups of neurons.Longer response times were observed in trials preceded by larger deviations from prototypical spike train (i.e., $\bar{D}$ values were positive), consistently for different subgroups of neurons with significant first reward versus repetition classification. After this classification, we ranked neurons according to I_max_ = max_q_ (<I>_t_), where the information was computed using the original Victor and Purpura metric. We formed different subgroups more and more restricted to high information neurons, as indicated. The smallest group was formed by applying a k-means algorithm (two clusters) and taking only the high-information cluster. **(a, b)** is for Monkey M, **(c, d)** for monkey P. **(a,c)** Bias score for $\bar{D}$ and ${\bar{D}}_{rate}$ as a function of the set of considered neurons. Neurons with fewer than five available trials were discarded. The *p*-value (2-sided permutation test) is indicated for each data point by the following symbols: small triangle for *p* ≤ 0.1; one star for *p* ≤ 0.05; two stars for *p* ≤ 0.01; three stars for *p* ≤ 0.001. Note that the values of ${\bar{D}}_{rate}$ in this figure are computed as in Fig 8 of the main text (assuming positive weighting of all neurons). We also did the rate analysis while assuming positive weights for neurons firing more during first reward and negative weights for the neurons firing more during repetition (the sign of ${\bar{D}}_{rate}$ was reversed for these latter neurons). The absolute value of the rate bias score reached by using this methodology was never higher than the best bias score reached by using a measure of deviation from prototypical spike train. Furthermore, this rate bias score reached *p* < 0.1 only once, for the smallest group of neurons of monkey M (bias score -11.8, *p* = 0.037). In addition, using this methodology also led to rate bias scores that were inconsistent between monkeys, as for monkey P these rate bias scores were always positive (and nonsignificant). **(b,d)** Comparison of $\bar{D}$ values between q_opt_ and q = 0. Here, q_opt_ was the temporal sensitivity that maximized discrimination between first reward and repetition using the normalized distance d* in each neuronal group (see S4 Text). Note that similar results were found when using q_opt_ = 10s^-1^ instead, i.e., the temporal sensitivity that maximized first reward discrimination when using the original Victor and Purpura distance as in main text. $\bar{D}$ was time-averaged (over analysis windows ending in [0.1,1]s, steps of 100 ms), separately for q_opt_ and q = 0. The resulting time-averages were compared with a signed rank test (*p*-value indicated). The boxplots represent the distribution of the difference of time-averaged $\bar{D}$ between q_opt_ and q = 0.(PDF)

S13 FigRelated to Fig 8. The relation between neural activity and behavior was still present when excluding trials with interruptions.Behavioral response time analysis while excluding post-first-reward trials that were interrupted before the monkey touches the target. These interruptions can be due to breaks of fixation or breaks in screen touch requirements, after which monkeys were forced to resume the sequence of actions (see Materials and Methods). Groups of neurons were formed as for S12 Fig **(a, b)** is for Monkey M, **(c, d)** for monkey P. **(a,c)** Bias score for $\bar{D}$ and ${\bar{D}}_{rate}$ as a function of the set of considered neurons. Neurons with less than 5 available trials were discarded. The *p*-value (2-sided permutation test) is indicated for each data point by the following symbols: small triangle for *p* ≤ 0.1; one star for *p* ≤ 0.05; two stars for *p* ≤ 0.01; three stars for *p* ≤ 0.001. Note that the values of ${\bar{D}}_{rate}$ in this figure are computed as in Fig 8 of the main text (assuming positive weighting of all neurons). We also did the rate analysis while assuming positive weights for neurons firing more during first reward and negative weights for the neurons firing more during repetition (the sign of ${\bar{D}}_{rate}$ was reversed for these latter neurons). This test never reached *p* < 0.05. **(b,d)** Comparison of $\bar{D}$ values between q_opt_ and q = 0. Here, q_opt_ was the temporal sensitivity that maximized discrimination between first reward and repetition using the normalized distance d* in each neuronal group (see S4 Text). Note that similar results were found when using q_opt_ = 10s^-1^ instead, i.e., the temporal sensitivity that maximized first reward discrimination when using the original Victor and Purpura distance as in main text. $\bar{D}$ was time-averaged (over analysis windows ending in [0.1,1]s, steps of 100 ms), separately for q_opt_ and q = 0. The resulting time-averages were compared with a signed rank test (*p*-value indicated). The boxplots represent the distribution of the difference of time-averaged $\bar{D}$ between q_opt_ and q = 0.(PDF)

S1 Table Related to Fig 4. Number of trials available in different task-epochs for the analyzed single neurons. Median (and 25th and 75th percentile) number of trials for single-units that were selected as significant. For the paired analysis, trial numbers were similar, with exceptions when the two waveforms were jointly reliable only during a subpart of the recording (leading to slightly fewer trials).
(PDF)

S2 Table Related to Fig 8. Probabilities of trial interruption or of mistake in the high and low response time groups. Difference of probability of mistakes or of mean number of trial interruptions after first reward, between problems with response time higher than median and problems with response time lower than median (response time measured between the first post-first-reward go signal and the post-first-reward touch). These interruptions can be due to break of fixation or break in screen touch requirements, after which monkeys were forced to resume the trial (see Materials and Methods). The medians (and means for differences in mistake probability) of these differences are shown together with a signed rank test measuring how significantly the median deviates from 0. Note that the overall percentage of mistakes was very small (0.81% and 1.0% in monkey M and monkey P, respectively, of considered trials).
(PDF)

S3 Table Related to Discussion and S2 Text. Comparison between pairs recorded on different electrodes versus the same electrode. Comparison of the distribution of k_opt_ values, and of the proportion of pairs with k_opt_ = 0, between pairs recorded on different electrodes versus the same electrode. There was no significant difference for first reward discrimination, contrary to a consistent bias towards lower k values in the same electrode group expected if waveforms from different neurons were not well separated between different templates. The difference observed exclusively during errors classification most likely results from a spatial organization of the inputs responsible for the firing of dACC neurons during the error task-epoch. This can lead to more similar neural responses for closeby neurons as compared to more distant neurons. See also S2 Text.
(PDF)

S1 Text Procedure for spike train metrics analysis method. (PDF)

S2 Text Procedure to test the negligibility of the influence spike sorting artifacts on results. (PDF)

S3 Text Procedure for saccades analysis. (PDF)

S4 Text Novel procedure for quantifying the deviation from prototypical spike train. (PDF)

## Abbreviations:

dACC: dorsal Anterior Cingulate Cortex
EPSPs: Excitatory Post Synaptic Potentials
LFP: local field potential
LIP: lateral intraparietal
MT: middle temporal
OFC: orbitofrontal cortex
PETH: peri-event time histogram
