# Supplementary material for: Spatiotemporal Spike Coding of Behavioral Adaptation in the Dorsal Anterior Cingulate Cortex
Source: PLoS Biol. 2015 Aug 12;13(8):e1002222. doi: 10.1371/journal.pbio.1002222 (PMC4534466; doi:10.1371/journal.pbio.1002222)
Supplement: S1 Fig — The improvement of decoding trough spike-timing sensitivity was robust in both monkeys. The left part of the figure describes the result of the discrimination between first reward and repetition, and the right part describes errors versus repetition discrimination. (a,b) Time course of the mean information over neurons, for different temporal sensitivities of the decoder (q) as indicated on the color scale, for monkey M and P respectively. (c) Difference of time-averaged information t (see Materials and Methods and main text Table 1) between temporal decoding (qopt ≈ 10 s-1) and spike-count decoding (q = 0 s-1). The p-value of a signed-rank test indicates that in both monkeys individually, temporal sensitivity induced a robust increase of information (all ps < 0.018). (PDF) [file pbio.1002222.s001.pdf]

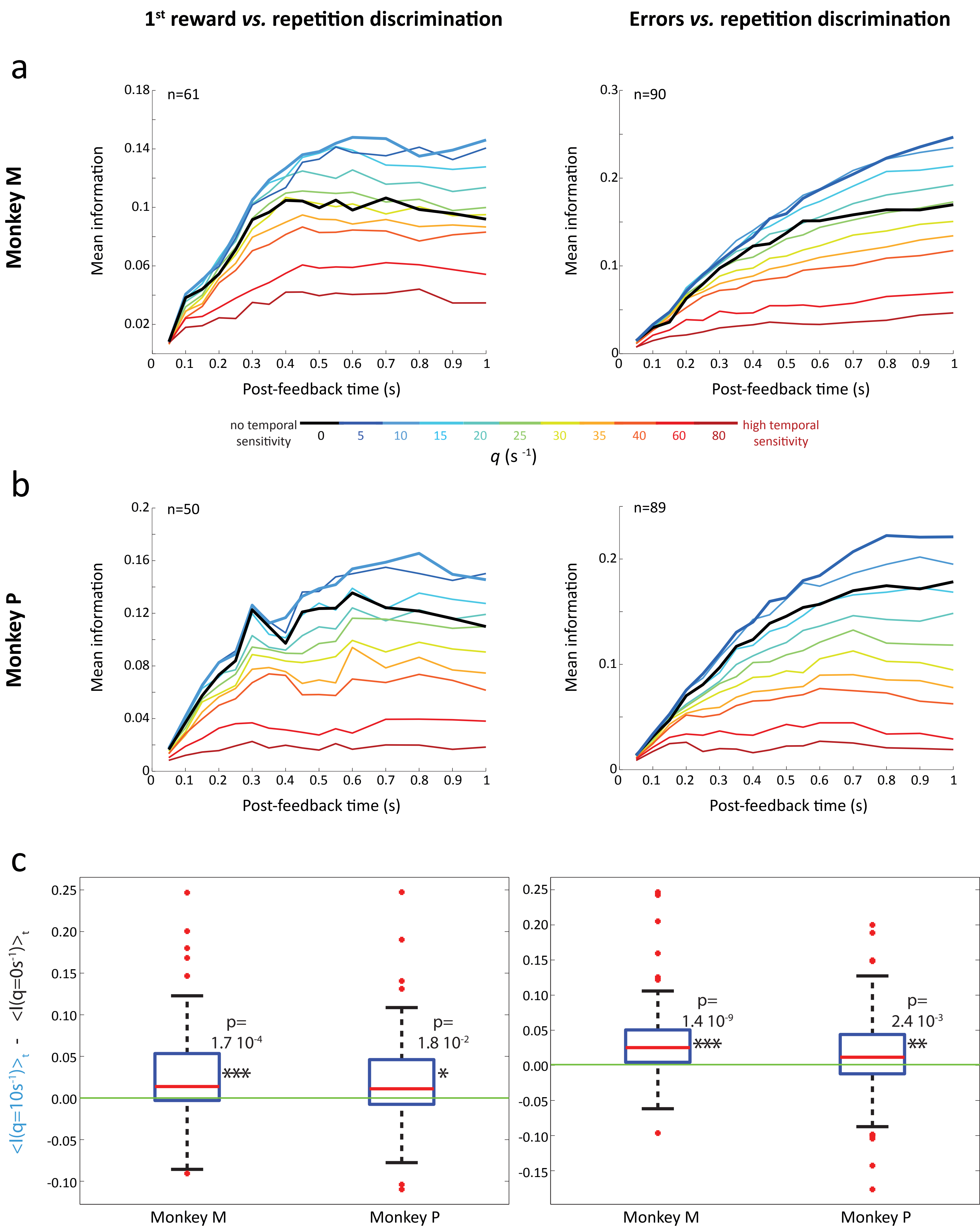

1st reward vs. repetition discrimination
Errors vs. repetition discrimination
a
Monkey M
n=61
n=90
Mean information
Post-feedback time (s)
no temporal sensitivity
high temporal sensitivity
0 5 10 15 20 25 30 35 40 60 80
q (s -1)
b
Monkey P
n=50
n=89
c
<I(q=10s-1)>t - <I(q=0s-1)>t
p= 1.7 10-4 ***
p= 1.8 10-2 *
p= 1.4 10-9 ***
p= 2.4 10-3 **
Monkey M
Monkey P
