# Supplementary figures and images for: Spatiotemporal Spike Coding of Behavioral Adaptation in the Dorsal Anterior Cingulate Cortex
Source: PLoS Biol. 2015 Aug 12;13(8):e1002222. doi: 10.1371/journal.pbio.1002222 (PMC4534466; doi:10.1371/journal.pbio.1002222)

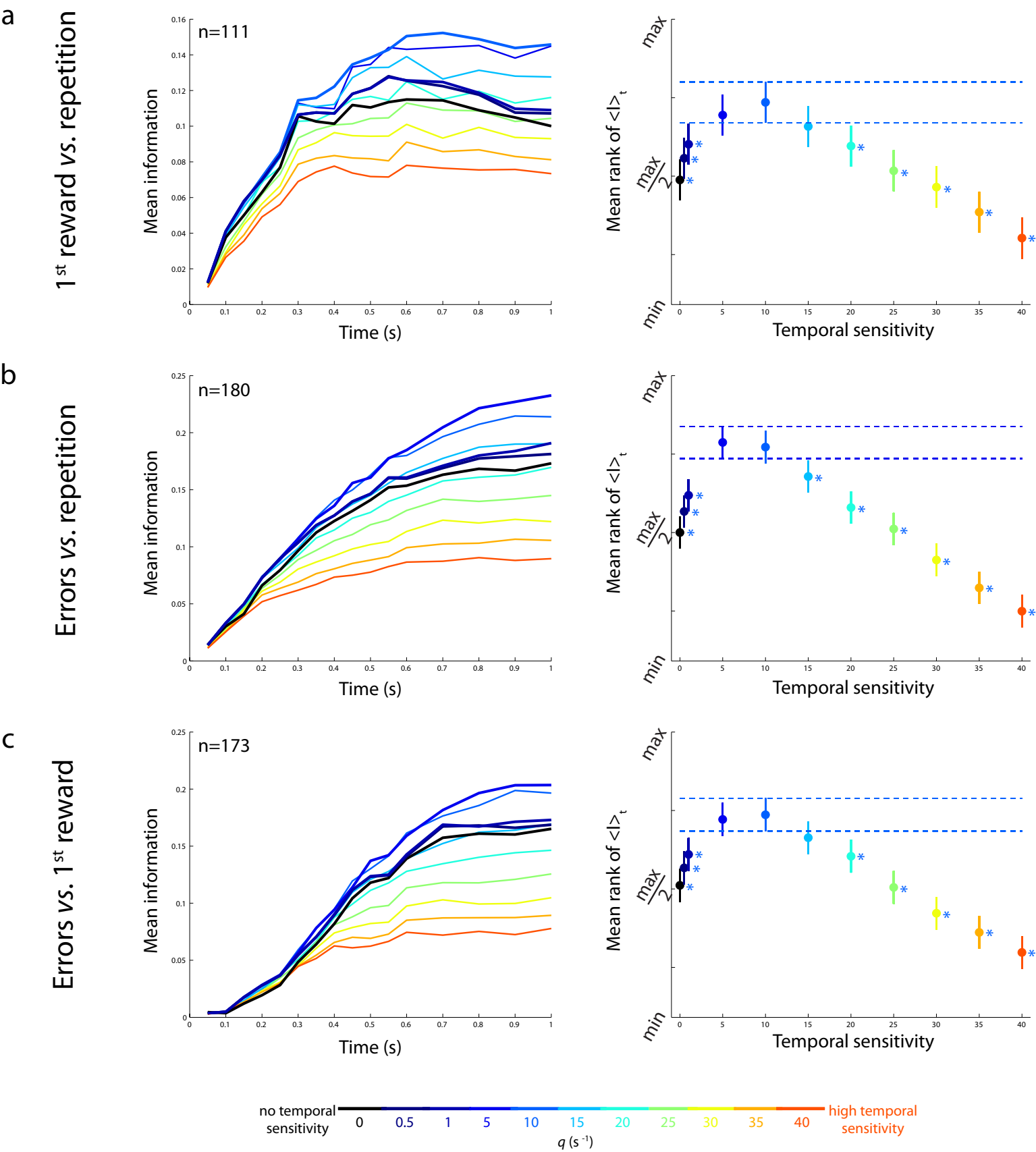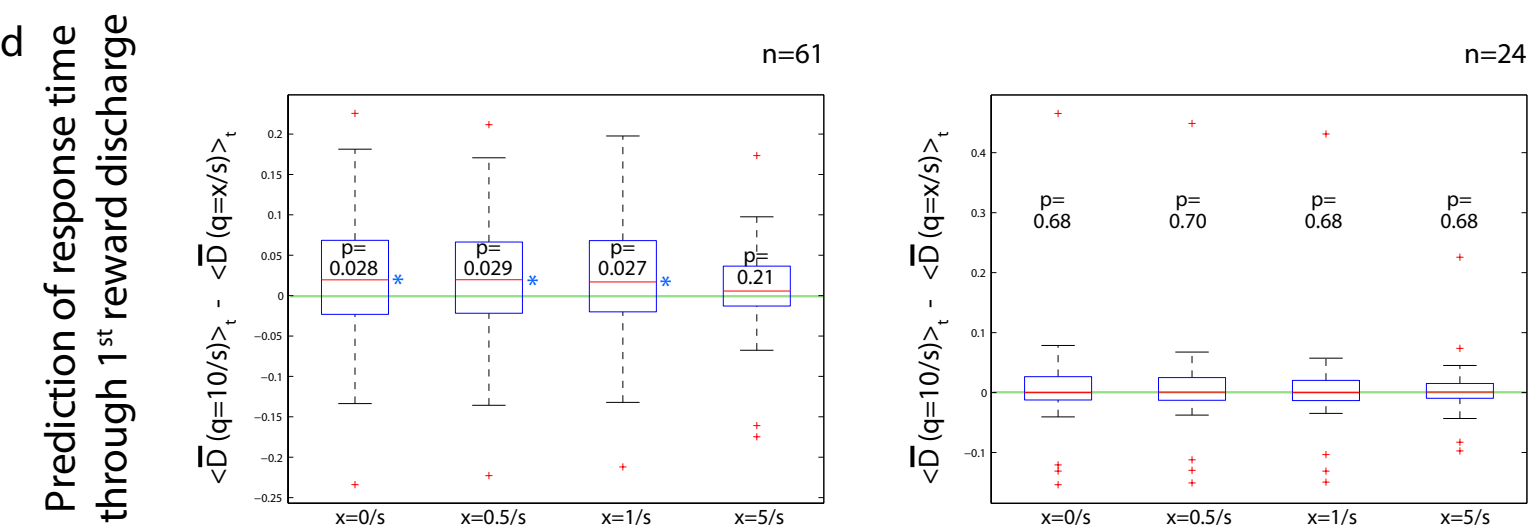

Supplement: S2 Fig — We test: (i) q = 0.5s-1, approximately equivalent to an exponential leak time-scale τ = (1/q) = 2s (main text Figs 1 and 2a and S1 Text). This is the minimal time-scale for a downstream leaky neuronal integrator which has to hold in memory the behavioral adaptation signals (and/or the behavioral strategy signals) for up to 3–6 s as required during the task (in case of fixation break). (ii) q = 1s-1, approximately equivalent to an exponentially decaying time-scale τ = 1s, as a more stringent test. (a,b,c) Classifying spike trains: first reward versus repetition (a), errors versus repetition (b), errors versus first reward (c). We used neurons reaching significant classification with any q-value (including q = 0.5 and 1s-1, permutation test, Methods), leading to only one more significant neuron compared to main text (for errors versus repetition classification, monkey P). Left: time course of the mean information over neurons. Right: results of post-hoc comparisons of the time-averaged information t after a Friedman anova, using the Tukey's honestly significant criterion correction. Q-values with significantly smaller performance than qopt are marked by a star. In all considered cases, both q = 0.5s-1 and q = 1s-1 were leading to significantly smaller t than qopt. In both monkeys individually, q = 0.5s-1 and q = 1s-1 had (at least qualitatively) lower average rank than q = 10s-1 and q = 5s-1. The Friedman test was restricted to q ≤ 40s-1, focusing on q-values for which classification was not too noisy. Finally, note that the slight differences in the rankings of q-values between the mean-information time course and Friedman anova are due to the fact that the mean is more sensitive to outliers with large values, while the average rank is determined by the consistency (over neurons) of the within-neuron rankings of t between different q-values. (d) Comparing 〈D¯〉t: the time-averaged index of behavioral prediction through deviation from prototypical first reward [file pbio.1002222.s002.pdf]

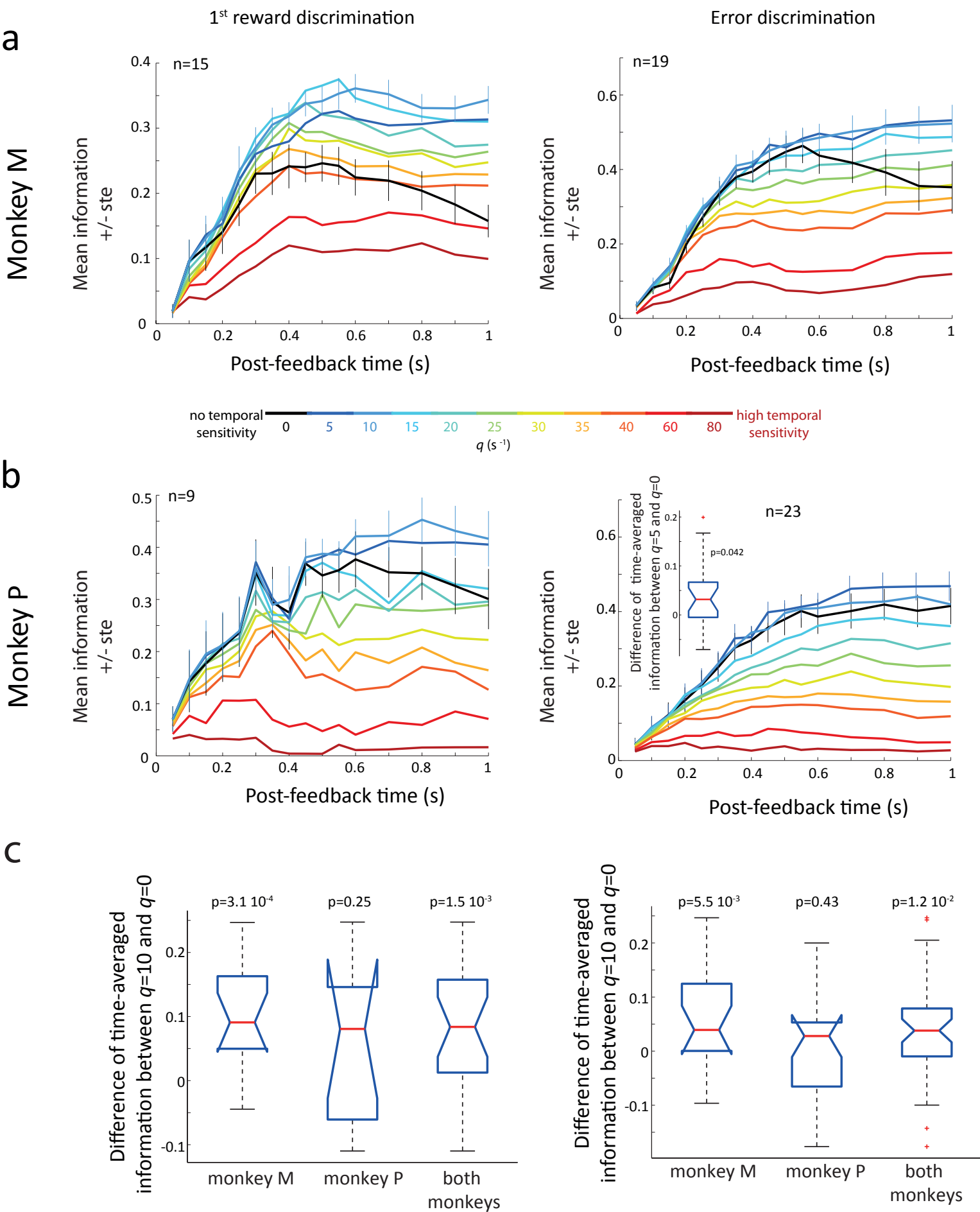

Supplement: S3 Fig — Spike-timing-sensitive decoding was also beneficial for very informative single neurons. We computed the maximum time-averaged information Imax for significant units (over q). Then, we used a k-means algorithm (with two groups) to separate populations with high versus low Imax. Results in this figure are for the high Imax neurons. (a,b) show the time course of the mean information (over neurons) for first reward (left) and errors (right) discrimination, as a function of timing sensitivity q, separately for the two monkeys. The inset in (b, right) shows the difference of time-averaged information t between q = 5s-1 (found optimal for monkey P over all significant units, for errors discrimination) and q = 0s-1. The p-value of a signed-rank test is indicated. (c) boxplots of the corresponding distributions of difference in t between q = 10 and q = 0 s-1. P-values of signed rank tests are indicated. Note that the notches indicate a confidence interval on the median, which may extend further away than the 25th or 75th quantiles, resulting in an inversion of the boxplot. (PDF) [file pbio.1002222.s003.pdf]

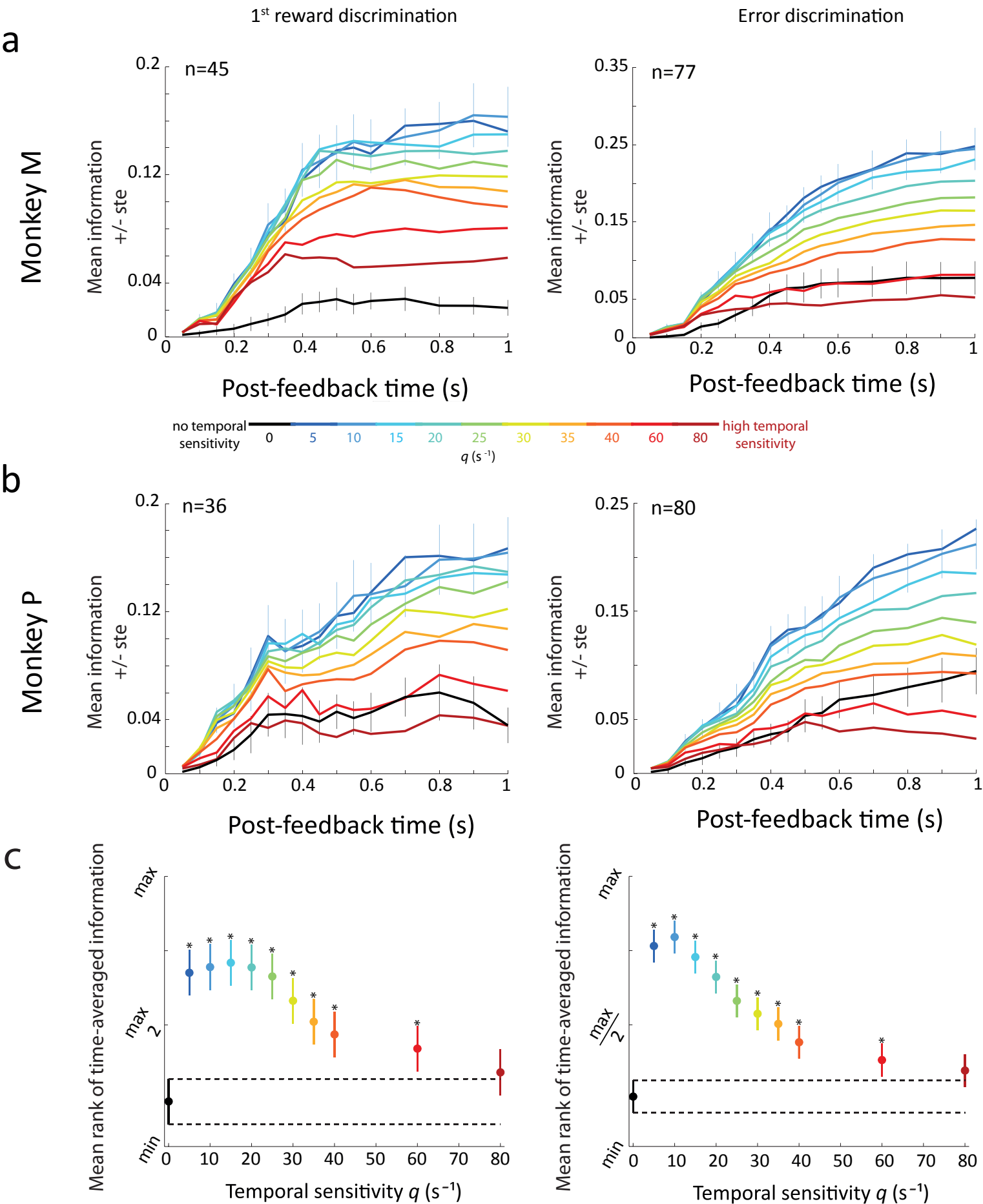

Supplement: S5 Fig — Information gain through temporal sensitivity was also observed when the classification of spike trains was biased toward smaller dissimilarities rather than determined by the median dissimilarity to spike trains of a task-epoch (see S1 Text, section: classification). Results in this figure are for the neurons with significant discrimination ability (permutation test, see main text Materials and Methods); note that the number of significant units is smaller than with the classification method of main text. (a,b) show the time course of the mean information (over neurons) for first reward (left) and errors (right) discrimination, as a function of timing sensitivity q, separately for the two monkeys. (c) Results of the post-hoc comparisons (with Tukey's honestly significant criterion correction for multiple comparisons) of a Friedman ANOVA comparing the time-averaged information t between temporal sensitivities. Note that the slight differences in the rankings of q-values between (a,b) and (c) are due to the fact that the mean over neurons is more sensitive to outliers with high values, while the average rank is determined by the consistency (over the population of single units) of the within-neuron rankings of t between different q-values. (PDF) [file pbio.1002222.s005.pdf]

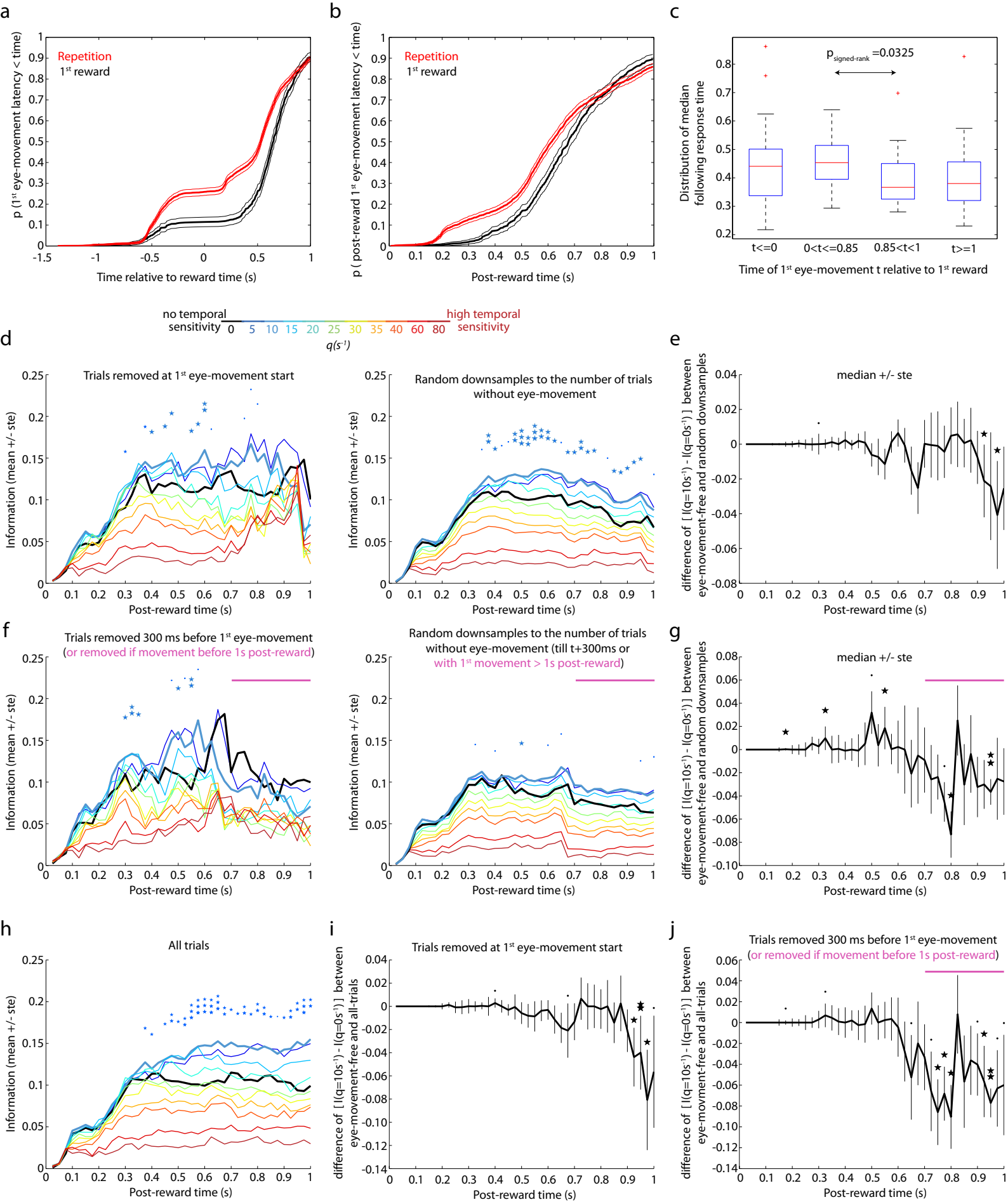

Supplement: S6 Fig — We observed temporal coding when using first reward versus repetition trials without detected eye-movements (for analysis windows <650ms). See S3 Text for methods. A small dot indicates p < 0.1, one star: p < 0.05, two stars: p < 0.01 for signed-rank tests. Error bars are standard error of the mean/median as in main text. (a,b,c) Behavior for 28 sessions (during which we recorded significant first reward versus repetition). (d,e,f,g,h,i,j) Decoding; (d,e,i) are related to the putative influence of motor feedback activity and (f,g,j) to the putative influence of premotor activity. 38 neurons were available for analysis windows <425 ms at least; for longer windows some neurons were excluded because no trials free of saccades were available. (a) Cumulative distribution function of first eye-movement latency following the fixation period. 95% confidence interval using Greenwood's formula. (b) as (a) but restricted to post-reward first eye-movement latency. (c) Distributions of median response times at the trial following first reward depending on the first eye-movement latency after the fixation period leading to first reward. (d) Left: Neuron-averaged information, while only trials without eye-movements detected yet were included. P-values compare between q = 10s-1 and q = 0s-1. Right: Neuron-averaged information for random downsamples (from all data) to the trial numbers of (d Left). The downsampling aims at excluding a possible effect of trial number when comparing data without (left) and with (right) saccades. For each neuron, the mean information among 1,000 downsamples was taken (taking the median gives similar results). P-values compare between q = 10s-1 and q = 0s-1. Note that the smoother aspect of the curves compared to the left graph likely results from the presence of an additional downsamplings-averaging in the right graph. Note also that, until plateau is reached (≈600 ms post-feedback), there were no robust differences in spike-count based information bet [file pbio.1002222.s006.pdf]

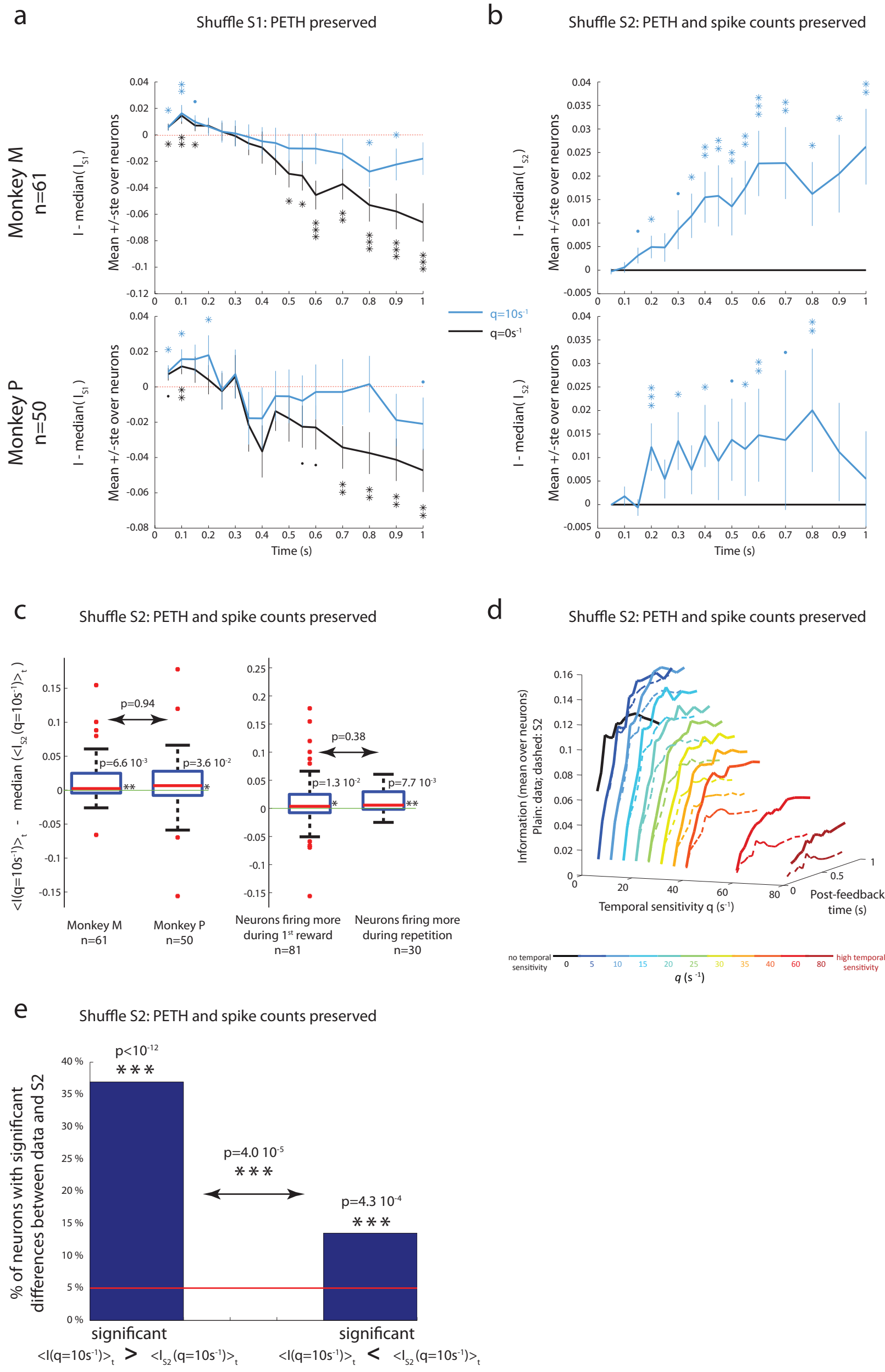

Supplement: S8 Fig — (a) The changes in information induced by performing shuffle 1 (preserving the time-dependent rate) were consistent over monkeys and were following the time course of the fano factors (see main text Fig 5). For long analyses windows, original data were less reliable than their spike-shuffled counterparts, while this effect was inverted for short analysis windows. The curves are the mean +/- standard error (ste, among all significant neurons for first reward versus repetition classification) of the difference between the information in the original data and the median information of the corresponding shuffled datasets. We show q = 0 (spike-count decoding, black) and q = 10s-1 ≈ qopt (blue). (b) Same conventions as in (a). The change in information induced by shuffling spikes according to shuffle 2 (preserving both time-dependent rate and spike count variability, see main text Fig 5) were consistent over monkeys. Original data had higher information than their spike-shuffled counterparts. (c) The distribution of difference of time-averaged information (<I(q = 10s-1 ≈ qopt)>t) between original data and the median for the corresponding datasets created by shuffle 2 was significantly positively biased for both monkeys (left) and for both the neurons firing more during first reward and the neurons firing more during repetition (signed-rank tests, all ps < 0.036). Note that qopt is unambiguously 10s-1 for neurons firing more during first reward (for these neurons q = 5s-1 and q = 15s-1 perform very similarly for original data decoding, see also S4c Fig). The distributions were not different between monkeys or between firing preference (ranked-sum tests, all ps > 0.38). (d) We show the means (over neurons) of (i) the information in original data, and of (ii) the median information of the corresponding shuffled datasets. For all q values, we observed higher information for the original data as compared to their shuffle 2 counterparts. The size of the effect increased for hig [file pbio.1002222.s008.pdf]

a

1<sup>st</sup> reward vs. repetition discrimination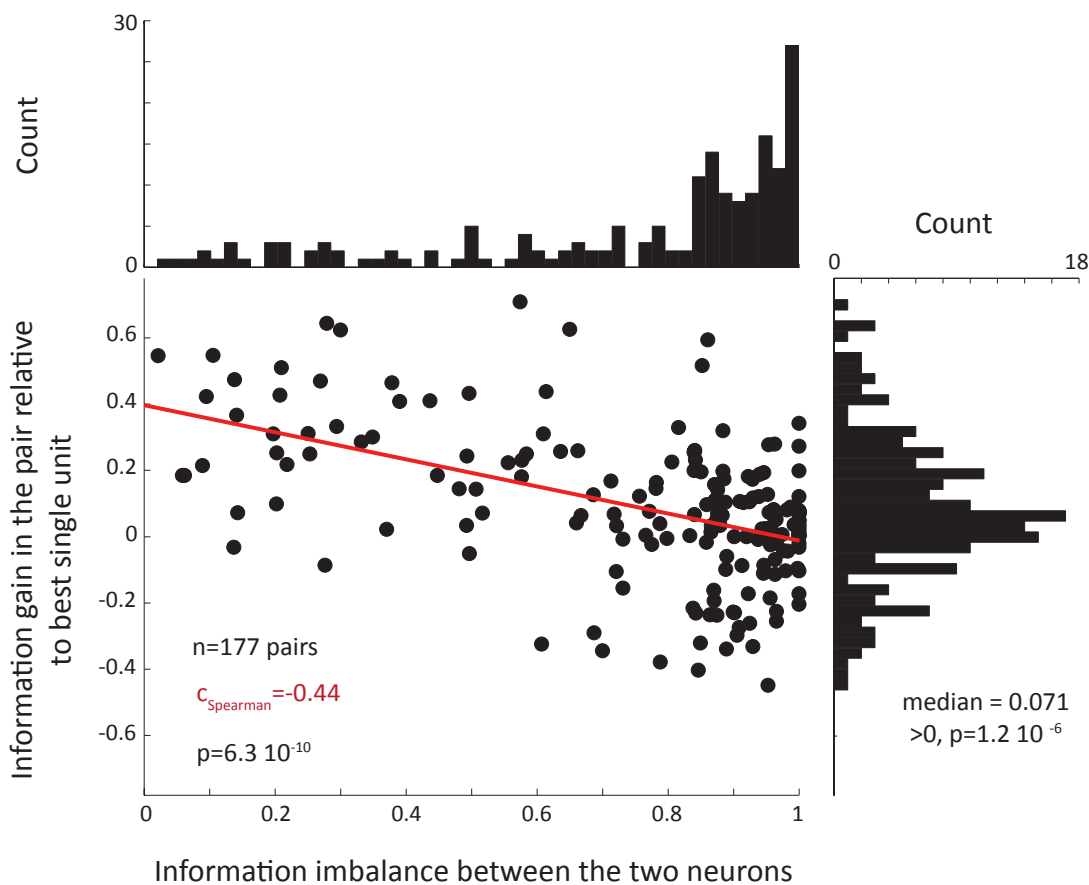

b

## Error vs. repetition discrimination

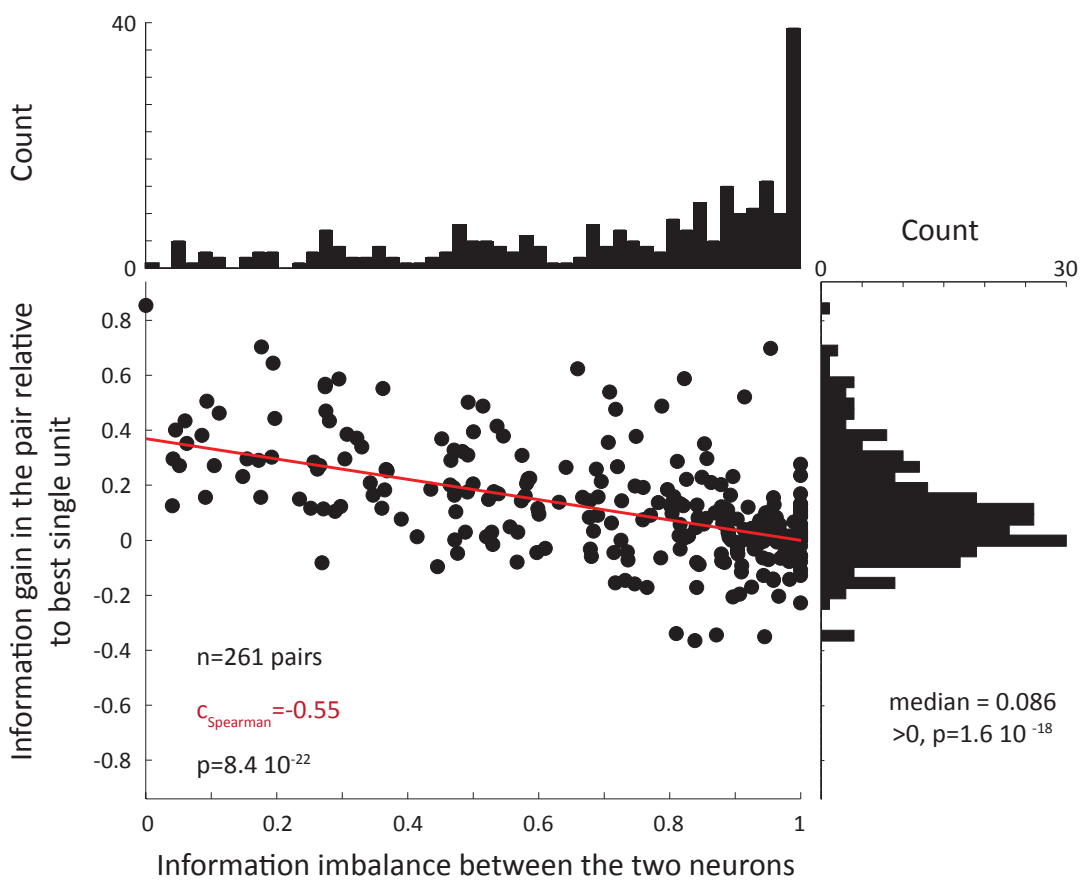

Supplement: S9 Fig — Paired spatial decoding led to increases in the amount of information despite imbalances in the discriminative power of single units. In this figure, only pairs with significant classification (permutation test) were included. (a) Discrimination between first reward and repetition task-epochs. The central plot shows the correlation between the information gain (obtained when decoding a neuron pair versus the pair’s most informative single unit, see main text Table 1) and the degree of information imbalance between the two units of a pair. A permutation test was used to determine the significance of the correlation (p < 0.001). The histograms at the top and right show the two marginal distributions. A signed-rank test was used to measure the significance of the bias towards an increase in the amount of information (i.e. positive gains, p < 0.001). (b) Same as (a) but for the discrimination between error and repetition task-epochs. (PDF) [file pbio.1002222.s009.pdf]

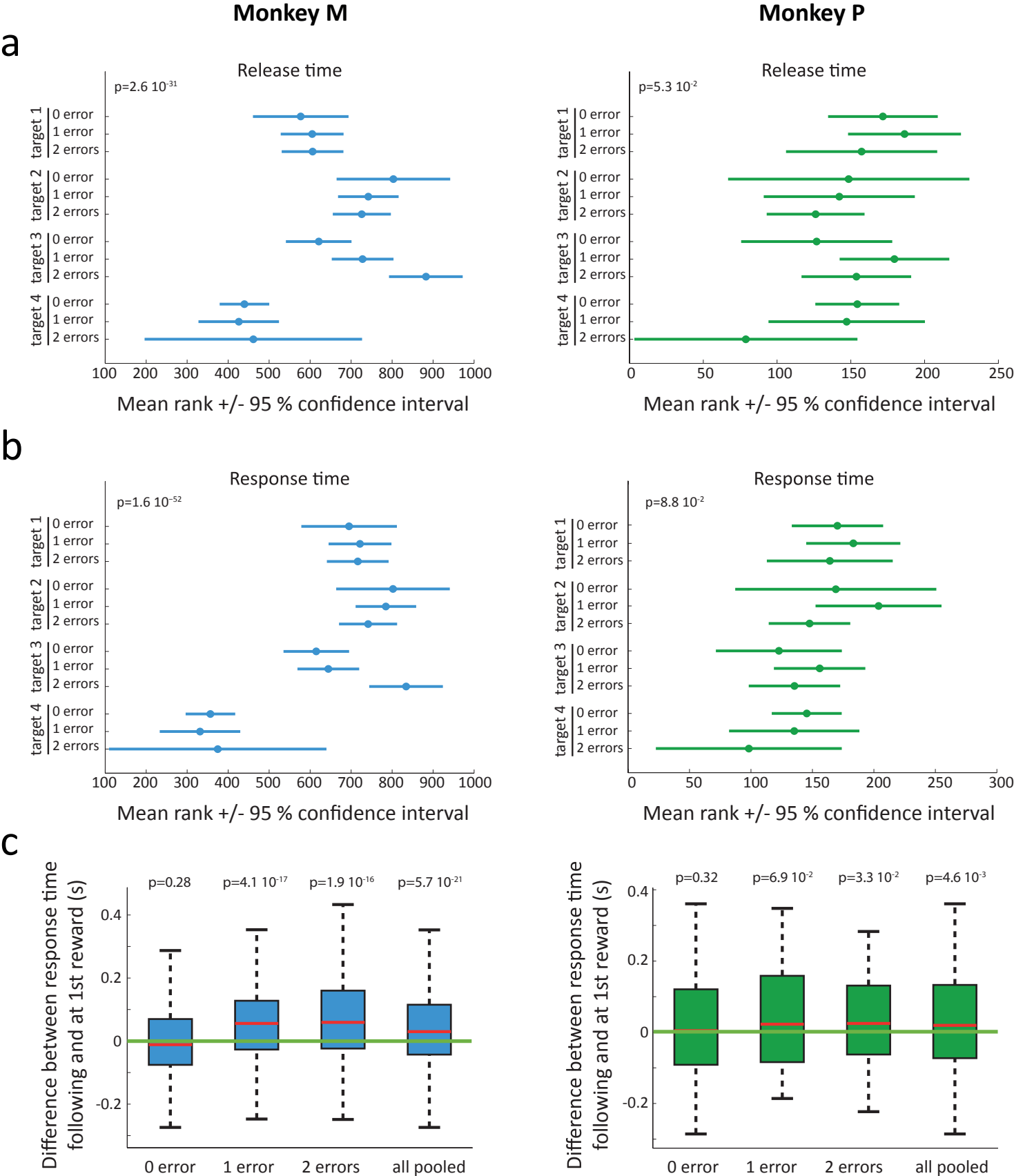

Supplement: S11 Fig — The analysis was restricted to the trials that were used for Fig 8 in the main text. (a) Modulation of the release time following first reward by the identity of the rewarding target and by the number of errors made preceding the first reward. The release time was defined as the time between the post-first-reward go signal for target touch (by the hand) and the release of the central lever button. Groups were compared with a non-parametric Kruskal-Wallis test (see p-value at the top-left). Post-hoc comparisons were conducted using Tukey's honestly significant criterion correction. Note that for all rewarding targets, the release movement occurred at the same place: on the central lever button. The release time modulation is therefore not likely to reflect motor constraints. (b) Modulation of the response time following first reward by the identity of the target and by the number of errors, conventions as in a). The response time was defined as the time between the post-first-reward go signal for target touch and the following target touch. The modulation of the response time was strikingly similar to the modulation of the release time (which, as argued above, is very unlikely to reflect motor constraints). In addition, note that while the two monkeys were in the same apparatus, they modulated their response time differently for the different targets. Finally, the target modulation of response time could interact with the modulation by the number of preceding errors. Altogether, the results argue against a purely motor cause for response time modulation, and rather point toward a spatial bias of cognitive processes. (c) Boxplots for the difference of response time between the trial following first reward (the first repetition, or, in rare cases, a mistake) and the trial that ended with the first reward, i.e., last exploration. The p-value of a signed rank test for a bias of the distribution toward either positive or negative values is indicated. The green line indica [file pbio.1002222.s011.pdf]

## Monkey M

a

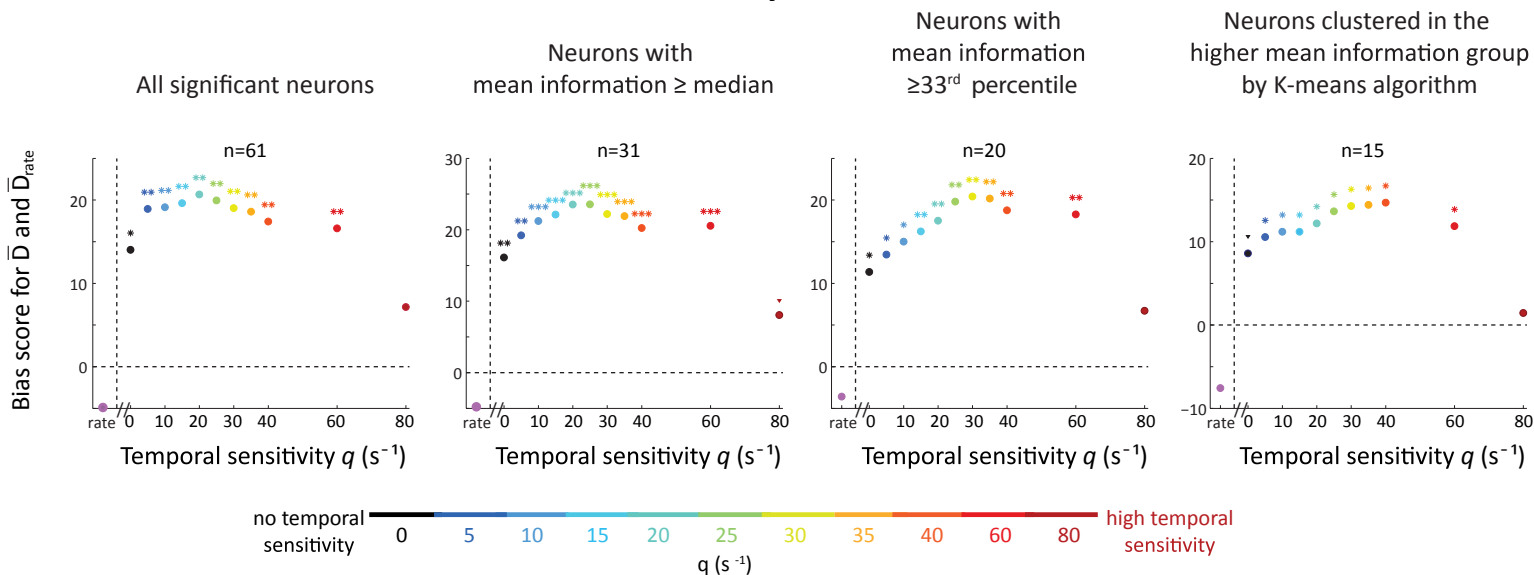

b

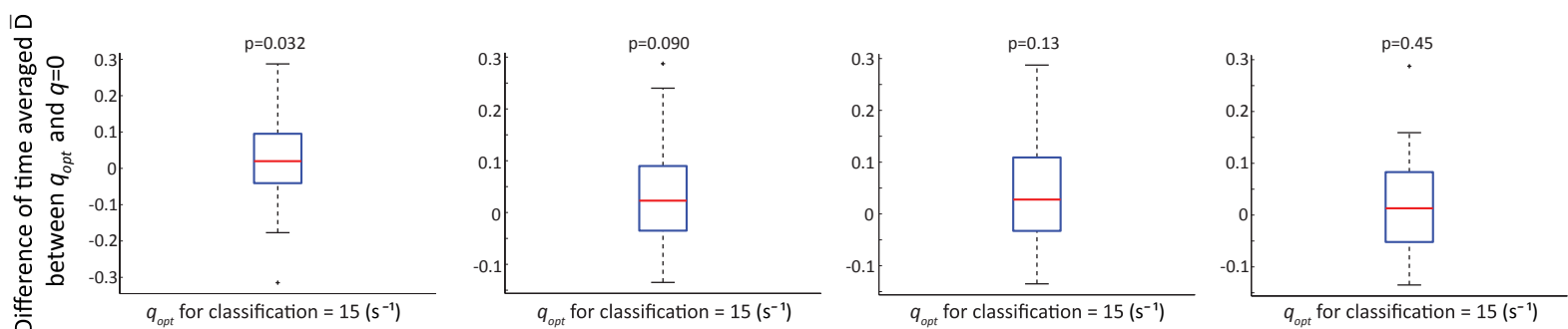

## Monkey P

c

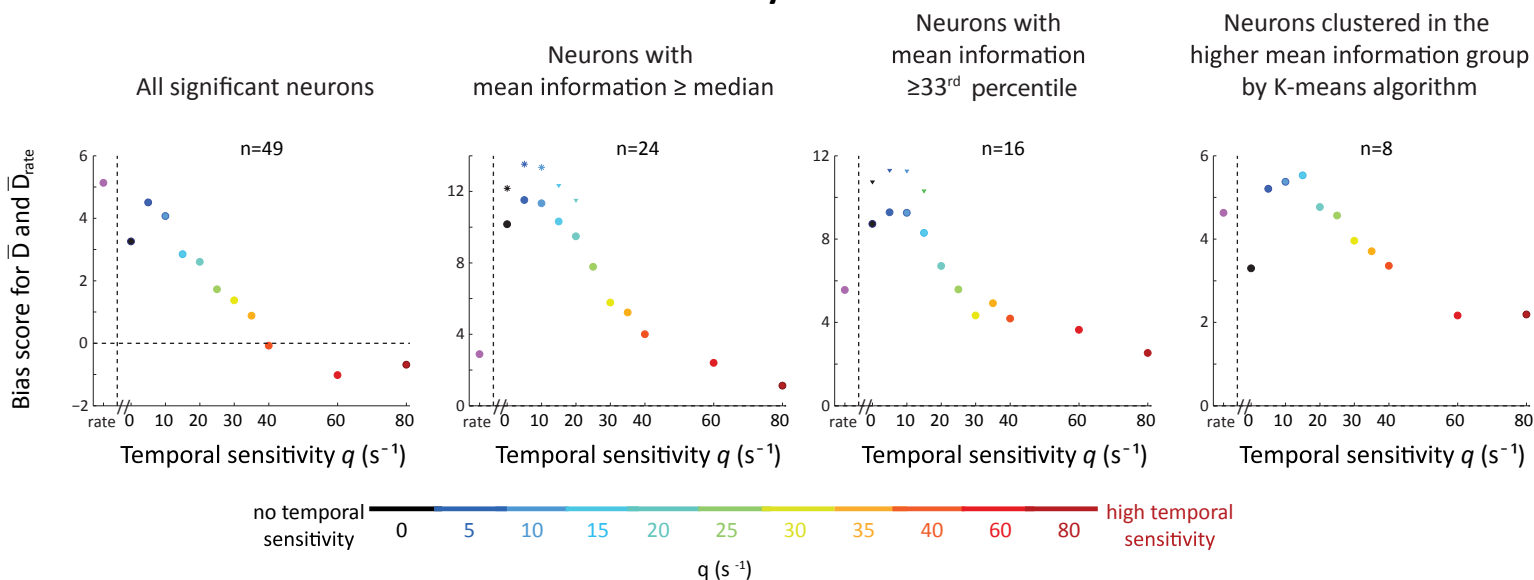

d

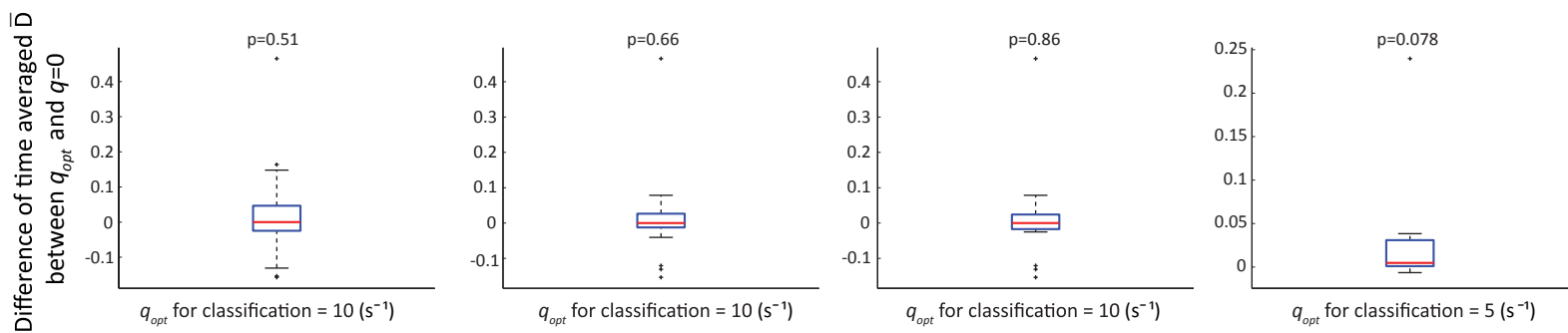

Supplement: S12 Fig — Longer response times were observed in trials preceded by larger deviations from prototypical spike train (i.e., D¯ values were positive), consistently for different subgroups of neurons with significant first reward versus repetition classification. After this classification, we ranked neurons according to Imax = maxq (t), where the information was computed using the original Victor and Purpura metric. We formed different subgroups more and more restricted to high information neurons, as indicated. The smallest group was formed by applying a k-means algorithm (two clusters) and taking only the high-information cluster. (a, b) is for Monkey M, (c, d) for monkey P. (a,c) Bias score for D¯ and D¯rate as a function of the set of considered neurons. Neurons with fewer than five available trials were discarded. The p-value (2-sided permutation test) is indicated for each data point by the following symbols: small triangle for p ≤ 0.1; one star for p ≤ 0.05; two stars for p ≤ 0.01; three stars for p ≤ 0.001. Note that the values of D¯rate in this figure are computed as in Fig 8 of the main text (assuming positive weighting of all neurons). We also did the rate analysis while assuming positive weights for neurons firing more during first reward and negative weights for the neurons firing more during repetition (the sign of D¯rate was reversed for these latter neurons). The absolute value of the rate bias score reached by using this methodology was never higher than the best bias score reached by using a measure of deviation from prototypical spike train. Furthermore, this rate bias score reached p < 0.1 only once, for the smallest group of neurons of monkey M (bias score -11.8, p = 0.037). In addition, using this methodology also led to rate bias scores that were inconsistent between monkeys, as for monkey P these rate bias scores were always positive (and nonsignificant). (b,d) Comparison of D¯ values between qopt and q = 0. Here, qopt was the temporal sensitivity that [file pbio.1002222.s012.pdf]

# Monkey M

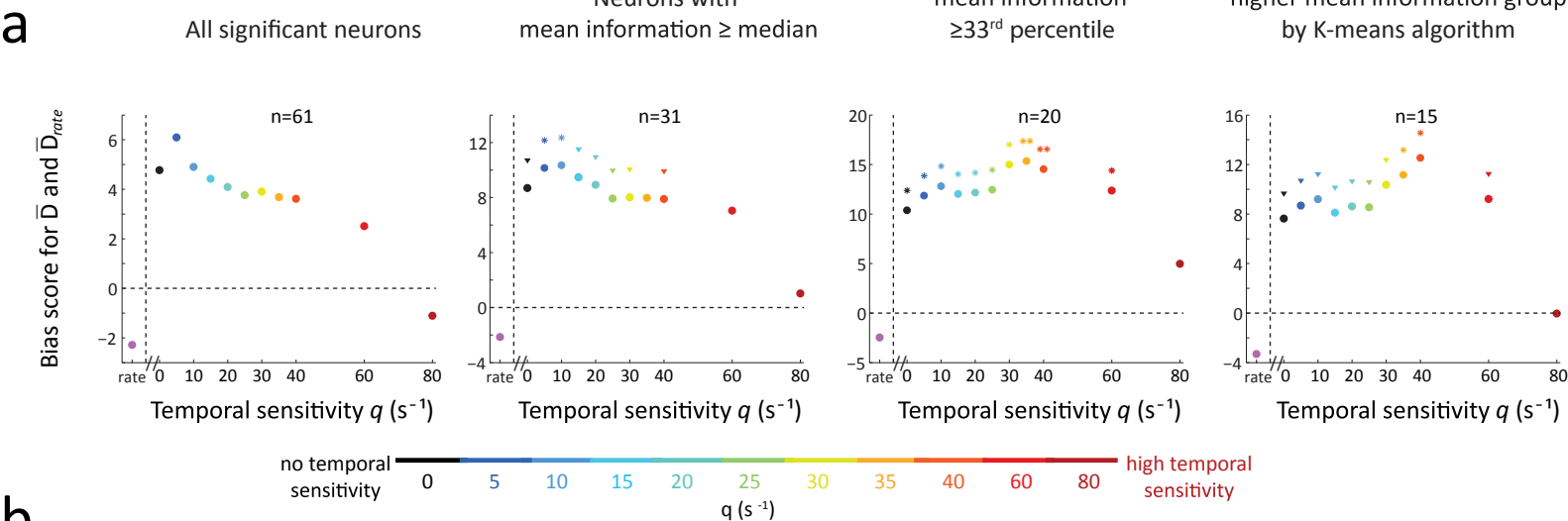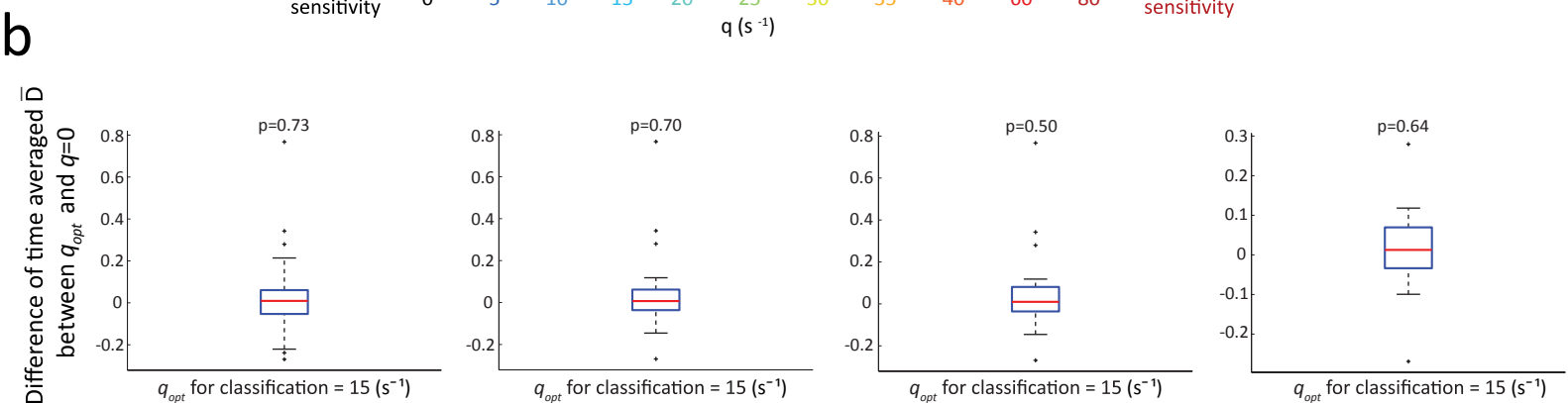

# Monkey P

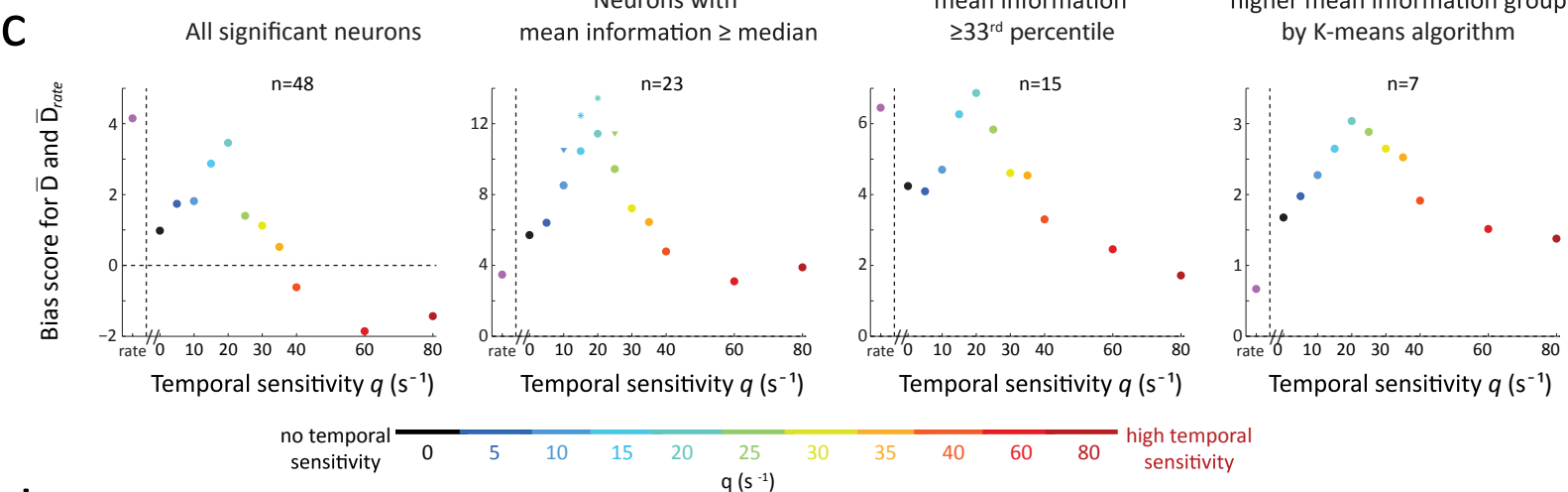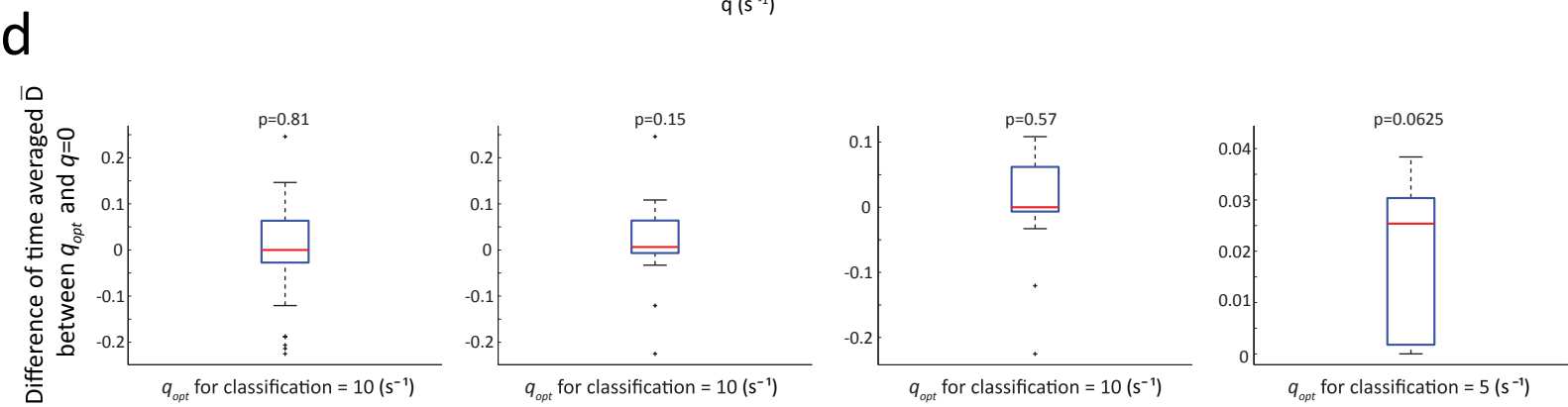

Supplement: S13 Fig — Behavioral response time analysis while excluding post-first-reward trials that were interrupted before the monkey touches the target. These interruptions can be due to breaks of fixation or breaks in screen touch requirements, after which monkeys were forced to resume the sequence of actions (see Materials and Methods). Groups of neurons were formed as for S12 Fig (a, b) is for Monkey M, (c, d) for monkey P. (a,c) Bias score for D¯ and D¯rate as a function of the set of considered neurons. Neurons with less than 5 available trials were discarded. The p-value (2-sided permutation test) is indicated for each data point by the following symbols: small triangle for p ≤ 0.1; one star for p ≤ 0.05; two stars for p ≤ 0.01; three stars for p ≤ 0.001. Note that the values of D¯rate in this figure are computed as in Fig 8 of the main text (assuming positive weighting of all neurons). We also did the rate analysis while assuming positive weights for neurons firing more during first reward and negative weights for the neurons firing more during repetition (the sign of D¯rate was reversed for these latter neurons). This test never reached p < 0.05. (b,d) Comparison of D¯ values between qopt and q = 0. Here, qopt was the temporal sensitivity that maximized discrimination between first reward and repetition using the normalized distance d* in each neuronal group (see S4 Text). Note that similar results were found when using qopt = 10s-1 instead, i.e., the temporal sensitivity that maximized first reward discrimination when using the original Victor and Purpura distance as in main text. D¯ was time-averaged (over analysis windows ending in [0.1,1]s, steps of 100 ms), separately for qopt and q = 0. The resulting time-averages were compared with a signed rank test (p-value indicated). The boxplots represent the distribution of the difference of time-averaged D¯ between qopt and q = 0. (PDF) [file pbio.1002222.s013.pdf]
