# Supplementary material for: Spatiotemporal Spike Coding of Behavioral Adaptation in the Dorsal Anterior Cingulate Cortex
Source: PLoS Biol. 2015 Aug 12;13(8):e1002222. doi: 10.1371/journal.pbio.1002222 (PMC4534466; doi:10.1371/journal.pbio.1002222)
Supplement: S4 Fig — We took advantage of the fact that the Victor and Purpura metrics scales with the number of spikes (see S1 Text and S4 Text) to compare the apparent firing reliability between task-epochs. For a given neuron, the qopt computed with this metric is expected to mostly reflect the spike-timing reliability of the task-epoch with more spikes, which spike trains are harder to classify. Indeed, within spike trains of this task-epoch, a small dissimilarity d can only be reached (and therefore correct classification can only happen) if the decoder detects a very small dissimilarity per spike and therefore a sufficiently small summed dissimilarity over all spikes. Hence, we compared groups of neurons firing preferentially in different task-epochs. Left: first reward versus repetition discrimination; right: errors versus repetition discrimination. (a) Difference of mean spike count in a [0.001,1]s post-feedback window between behavioral adaptation and repetition epochs. (b, Left) Boxplots of the distributions of qopt values for cells discharging preferentially during behavioral adaptation versus repetition (the notches indicate an approximate confidence interval on the median, which may extend beyond the quartiles). P-values of ranked sum tests comparing medians are shown. The higher qopts for neurons firing more during behavioral adaptation could reflect a real higher reliability of firing, or the fact that the temporal reference used for the analysis was more reliably locked to the monkey's internal reference during behavioral adaptation epochs. Indeed, monkeys could anticipate the outcome before the feedback was explicitly given during repetition, potentially leading to a trial-specific advance of dACC firing compared to feedback time. (b, Right) Boxplots of the distributions of time-averaged information t for q = 0s-1. P-values of ranked sum tests are shown. The absence of significant difference suggests that the difference in qopt (left) reflects a difference in spike-t [file pbio.1002222.s004.pdf]

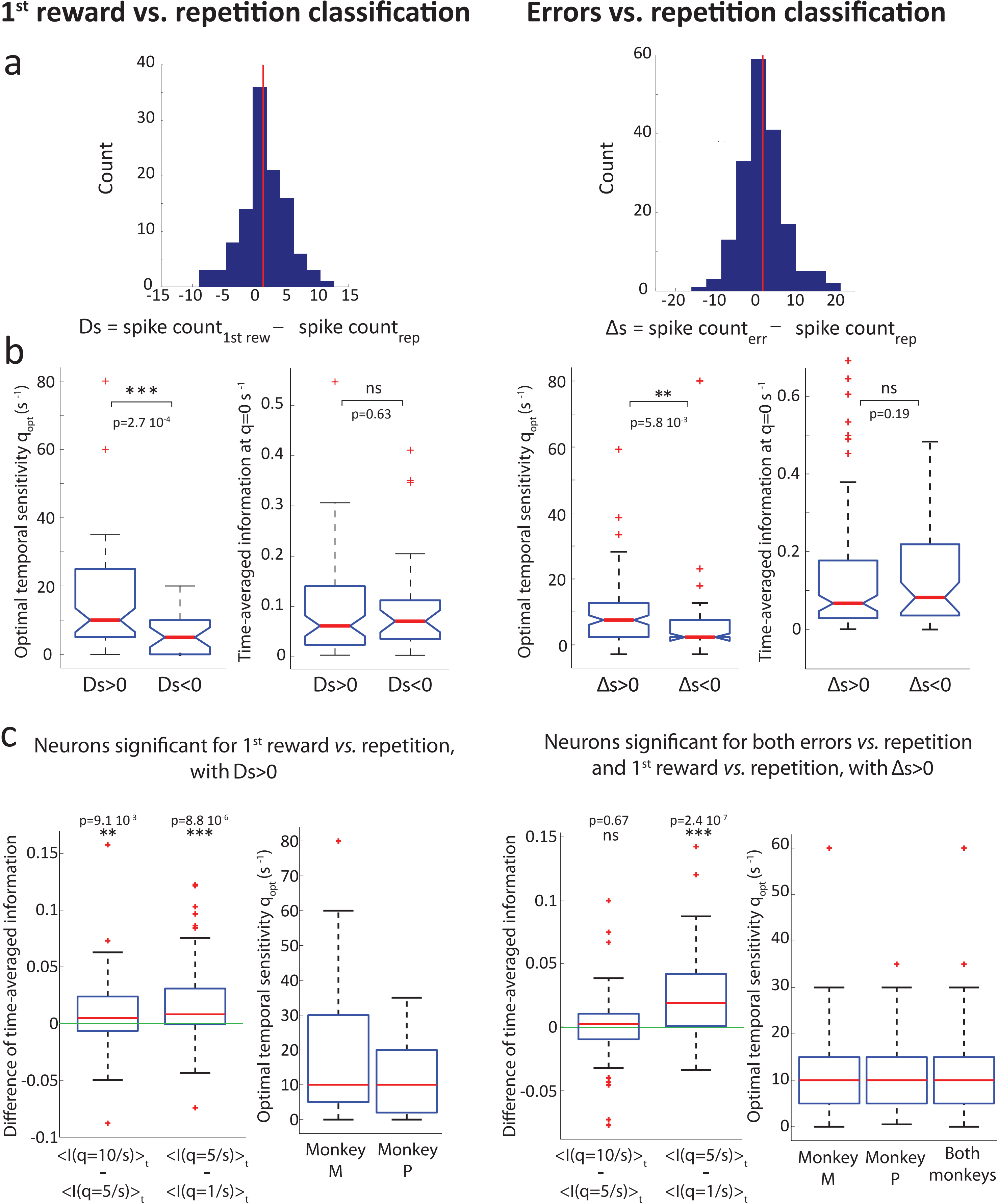
1st reward vs. repetition classification
Errors vs. repetition classification
a
Count
Ds = spike count1st rew − spike countrep
Δs = spike counterr − spike countrep
b
Optimal temporal sensitivity qopt (s-1)
Time-averaged information at q=0 s-1
***
p=2.7 10-4
ns
p=0.63
**
p=5.8 10-3
ns
p=0.19
Ds>0
Ds<0
Δs>0
Δs<0
c
Neurons significant for 1st reward vs. repetition, with Ds>0
Neurons significant for both errors vs. repetition and 1st reward vs. repetition, with Δs>0
Difference of time-averaged information
p=9.1 10-3
**
p=8.8 10-6
***
p=0.67
ns
p=2.4 10-7
***
<I(q=10/s)>t - <I(q=5/s)>t
<I(q=5/s)>t - <I(q=1/s)>t
Monkey M
Monkey P
Both monkeys
