# Supplementary material for: Spatiotemporal Spike Coding of Behavioral Adaptation in the Dorsal Anterior Cingulate Cortex
Source: PLoS Biol. 2015 Aug 12;13(8):e1002222. doi: 10.1371/journal.pbio.1002222 (PMC4534466; doi:10.1371/journal.pbio.1002222)
Supplement: S7 Fig — The data suggest an advantage of spike-timing sensitivity for decoding the identity of the adapted behavioral strategy (exploration or switch). (a,b,c) Single unit decoding between errors and first reward spike trains, for all neurons with significant errors versus first reward classification. (a,b) Time course of the mean information for different temporal sensitivities as indicated in the colorbar, for monkey M (a) and monkey P (b). (c) Mean rank (+/-95% confidence interval) of post hoc comparisons (using Tukey's honestly significant criterion correction for multiple comparisons) of a Friedman ANOVA comparing the time-averaged information t. The average was taken over analysis windows ending between 0.1 s and 1 s with steps of 0.1 s. Data from both monkeys were pooled. (d,e,f) Decoding performance for errors versus first reward classification, restricted to neurons that were significant for both errors versus first reward classification and first reward versus repetition classification. The discharge of these neurons cannot therefore be merely related to the reward quantity received by the monkey, instead they appear correlated with the nature of the adapted behavioral strategy. (d) Time course of mean information for different temporal sensitivities as indicated in the colorbar (data from both monkeys pooled). (e) Mean rank (+/-95% confidence interval) of post hoc comparisons (using Tukey's honestly significant criterion correction for multiple comparison) of a Friedman ANOVA comparing the time-averaged information t. Note that the slight differences in the rankings of q-values between the mean information and the Friedman anova graphs are due to the fact that the mean (up) is more sensitive to outliers with large values, while the average rank (down) is determined by the consistency (over neurons) of the within-neuron rankings of t between different q-values. These outliers are, for instance, visible in monkey P in (f). Note that some of these outliers [file pbio.1002222.s007.pdf]

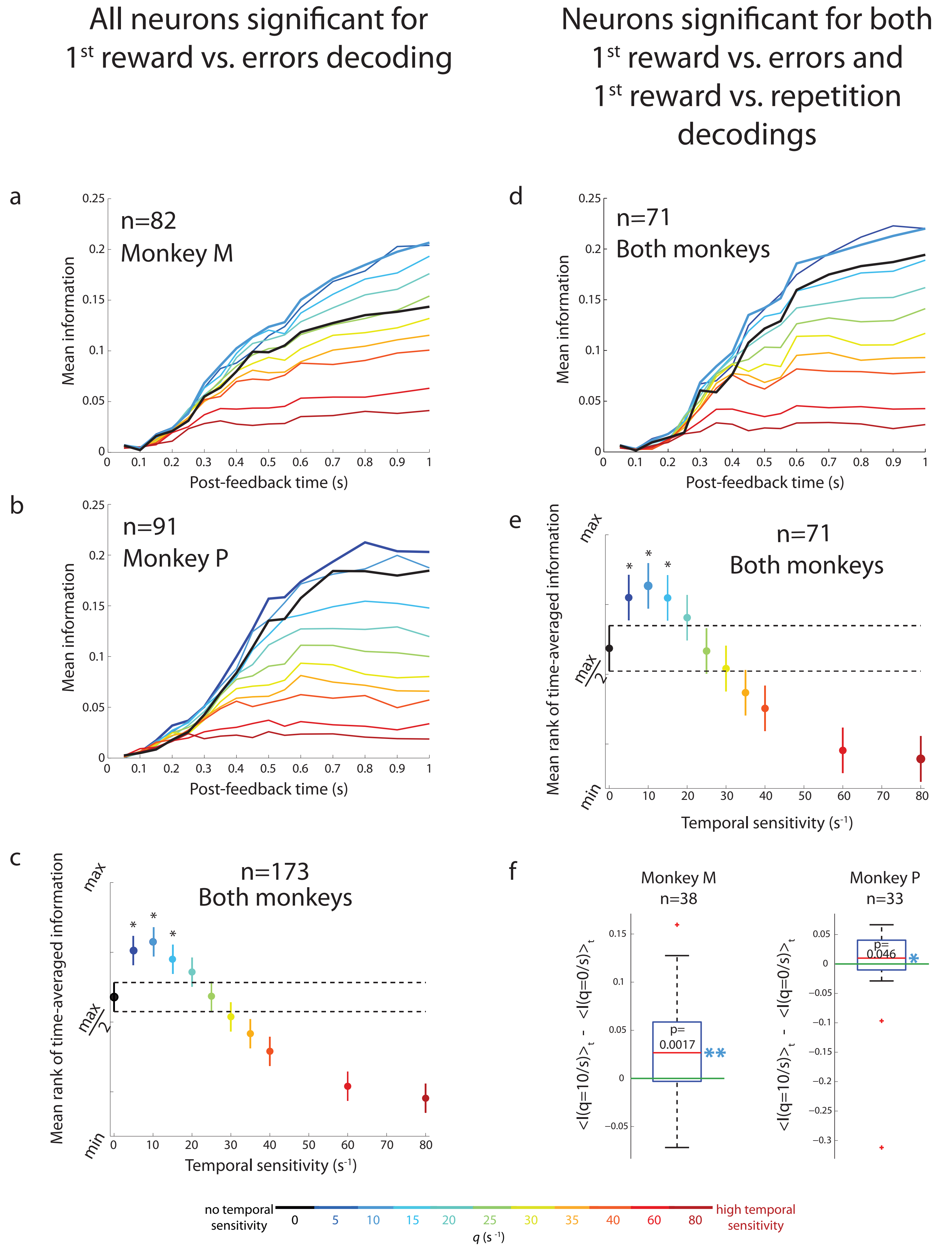

All neurons significant for
1st reward vs. errors decoding
Neurons significant for both
1st reward vs. errors and
1st reward vs. repetition
decodings
a
n=82
Monkey M
Mean information
Post-feedback time (s)
b
n=91
Monkey P
Mean information
Post-feedback time (s)
c
n=173
Both monkeys
Mean rank of time-averaged information
max
max/2
min
Temporal sensitivity (s-1)
d
n=71
Both monkeys
Mean information
Post-feedback time (s)
e
n=71
Both monkeys
Mean rank of time-averaged information
Temporal sensitivity (s-1)
f
Monkey M
n=38
p=
0.0017
<I(q=10/s)>t - <I(q=0/s)>t
Monkey P
n=33
p=
0.046
no temporal
sensitivity
0
5
10
15
20
25
30
35
40
60
80
q (s-1)
high temporal
sensitivity
