# Supplementary material for: Spatiotemporal Spike Coding of Behavioral Adaptation in the Dorsal Anterior Cingulate Cortex
Source: PLoS Biol. 2015 Aug 12;13(8):e1002222. doi: 10.1371/journal.pbio.1002222 (PMC4534466; doi:10.1371/journal.pbio.1002222)
Supplement: S10 Fig — (a) Time course of the mean information among neuron pairs with significant discrimination. Different sheets with different green shadings are different analysis window durations, as indicated on the color scale on the right. For both monkeys and consistently over analysis windows, information increased with adapted temporal sensitivity compared to spike count decoding (q = 0s-1), and on average the information was larger for intermediate-to-large values of the discrimination between neurons (k). In this figure, only pairs with significant classification (permutation test) were included, as in the bottom part of main text Fig 6. (b) Left: maximum (over time-windows) mean (over pairs) information for first reward and error discrimination, as a function of timing sensitivity q and between-unit discrimination degree k. Information was maximized over analysis windows ending in [0.05,0.6]s, steps of 50 ms, and in [0.7,1]s, steps of 100ms. Right: comparison of (q, k) for the time-averaged information t of pairs of neurons. The plots display the results of post hoc comparisons (using Tukey's honestly significant criterion correction after a Friedman ANOVA) between t (computed with analysis windows ending in [0.1,1]s, steps 100 ms). The red dot marks the (q,k)opt value leading to the higher rank; black dots mark (q,k) values that are not significantly different from (q,k)opt, and green dots mark (q,k) values that have significantly smaller ranks than (q,k)opt. (PDF) [file pbio.1002222.s010.pdf]

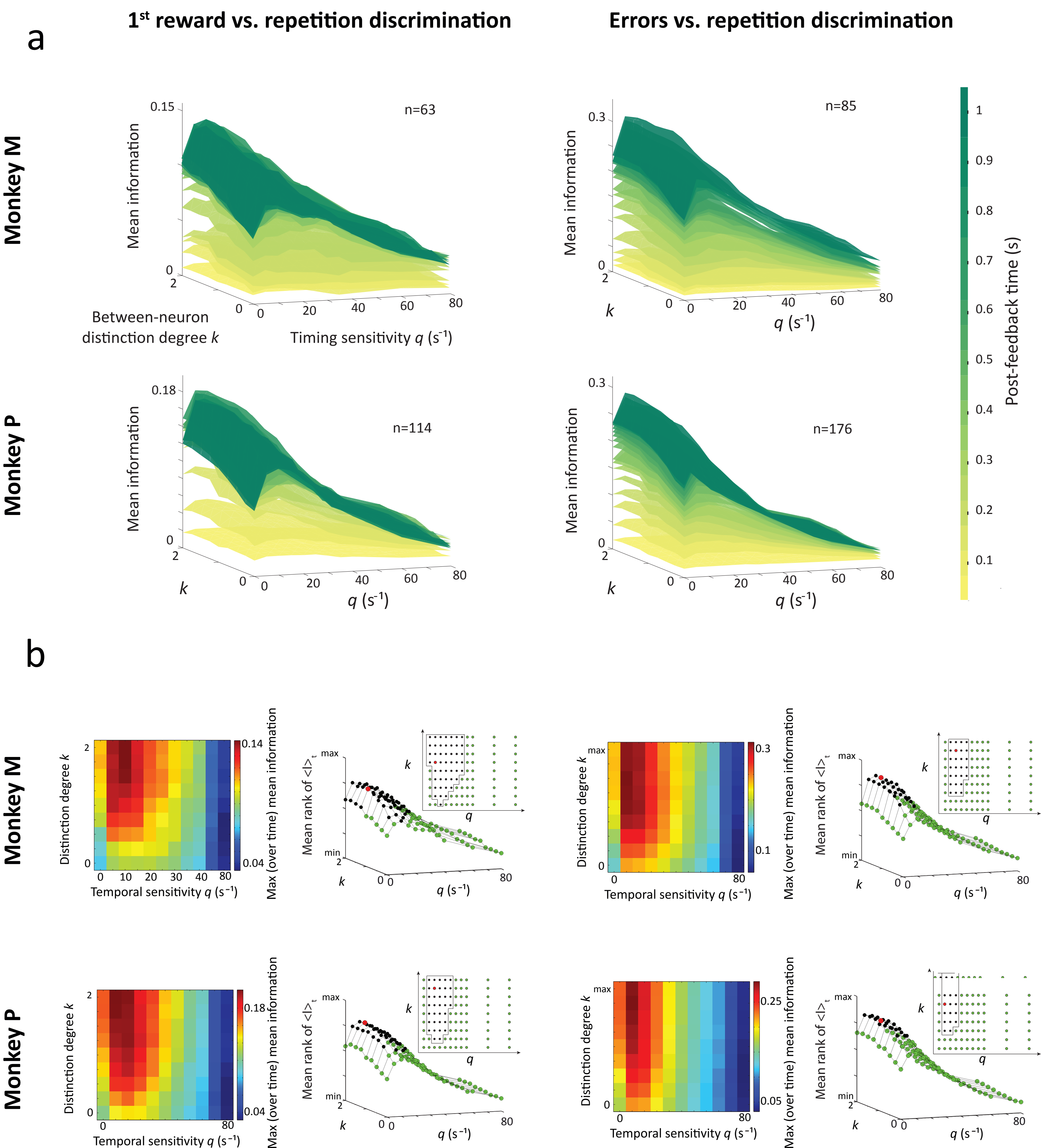
a
1st reward vs. repetition discrimination
Errors vs. repetition discrimination
Monkey M
Monkey P
Mean information
n=63
n=85
n=114
n=176
Between-neuron distinction degree k
Timing sensitivity q (s-1)
q (s-1)
k
Post-feedback time (s)
b
Distinction degree k
Temporal sensitivity q (s-1)
Max (over time) mean information
Mean rank of <I>t
max
min
