# Supplementary material for: Spatiotemporal Spike Coding of Behavioral Adaptation in the Dorsal Anterior Cingulate Cortex
Source: PLoS Biol. 2015 Aug 12;13(8):e1002222. doi: 10.1371/journal.pbio.1002222 (PMC4534466; doi:10.1371/journal.pbio.1002222)
Supplement: S1 Table — Median (and 25th and 75th percentile) number of trials for single-units that were selected as significant. For the paired analysis, trial numbers were similar, with exceptions when the two waveforms were jointly reliable only during a subpart of the recording (leading to slightly fewer trials). (PDF) [file pbio.1002222.s014.pdf]

		Behavioral adaptation	Repetition
Monkey M	1st reward discrimination	30 (16-40)	97 (62-130.25)
	Error discrimination	38 (27-47)	88.5 (68-120)
Monkey P	1st reward discrimination	17 (14-21)	60.5 (50-69)
	Error discrimination	27 (21-32)	59 (47.5-71)
