# Supplementary material for: Spatiotemporal Spike Coding of Behavioral Adaptation in the Dorsal Anterior Cingulate Cortex
Source: PLoS Biol. 2015 Aug 12;13(8):e1002222. doi: 10.1371/journal.pbio.1002222 (PMC4534466; doi:10.1371/journal.pbio.1002222)
Supplement: S2 Table — Difference of probability of mistakes or of mean number of trial interruptions after first reward, between problems with response time higher than median and problems with response time lower than median (response time measured between the first post-first-reward go signal and the post-first-reward touch). These interruptions can be due to break of fixation or break in screen touch requirements, after which monkeys were forced to resume the trial (see Materials and Methods). The medians (and means for differences in mistake probability) of these differences are shown together with a signed rank test measuring how significantly the median deviates from 0. Note that the overall percentage of mistakes was very small (0.81% and 1.0% in monkey M and monkey P, respectively, of considered trials). (PDF) [file pbio.1002222.s015.pdf]

	**Monkey M**	**Monkey P**	**Both monkeys**
Difference of mean number of aborted trials	Median=0.19<br>$p_{signrank}$=5.3 10^{-4}	Median=0.095<br>$p_{signrank}$=0.15	Median=0.16<br>$p_{signrank}$=3.2 10^{-4}
Difference of probability of errors	Median=0, mean=-9.2 10^{-3}<br>$p_{signrank}$=0.31	Median=0, mean=9.7 10^{-4}<br>$p_{signrank}$=1	Median=0, mean=-5.9 10^{-3}<br>$p_{signrank}$=0.36
