# Supplementary material for: Spatiotemporal Spike Coding of Behavioral Adaptation in the Dorsal Anterior Cingulate Cortex
Source: PLoS Biol. 2015 Aug 12;13(8):e1002222. doi: 10.1371/journal.pbio.1002222 (PMC4534466; doi:10.1371/journal.pbio.1002222)
Supplement: S3 Table — Comparison of the distribution of kopt values, and of the proportion of pairs with kopt = 0, between pairs recorded on different electrodes versus the same electrode. There was no significant difference for first reward discrimination, contrary to a consistent bias towards lower k values in the same electrode group expected if waveforms from different neurons were not well separated between different templates. The difference observed exclusively during errors classification most likely results from a spatial organization of the inputs responsible for the firing of dACC neurons during the error task-epoch. This can lead to more similar neural responses for closeby neurons as compared to more distant neurons. See also S2 Text. (PDF) [file pbio.1002222.s016.pdf]

	**1st reward discrimination**			**Errors discrimination**		
	**Monkey M**	**Monkey P**	**Both monkeys**	**Monkey M**	**Monkey P**	**Both monkeys**
**Distribution of k_{opt}**	Diff. electrodes<br><br>mean=1.19<br>median=1.5	Diff. electrodes<br><br>mean=1.13<br>median=1.25	Diff. electrodes<br><br>mean=1.15<br>median=1.25	Diff. electrodes<br><br>mean=1.48<br>median=1.75	Diff. electrodes<br><br>mean=1.17<br>median=1.25	Diff. electrodes<br><br>mean=1.26<br>median=1.5
	Same electrode<br><br>mean=1.21<br>median=1.375	Same electrode<br><br>mean=1.16<br>median=1.25	Same electrode<br><br>mean=1.18<br>median=1.25	Same electrode<br><br>mean=1.08<br>median=1.125	Same electrode<br><br>mean=0.87<br>median=1	Same electrode<br><br>mean=0.96<br>median=1
	$p_{ranksum}$=0.95	$p_{ranksum}$=0.81	$p_{ranksum}$=0.83	$p_{ranksum}$=0.020	$p_{ranksum}$=0.012	$p_{ranksum}=2.0\ 10^{-3}$
**Proportion of pairs for which k_{opt}=0**	Diff. electrodes<br>4/43=0.093	Diff. electrodes<br>15/82=0.18	Diff. electrodes<br>19/125=0.15	Diff. electrodes<br>3/51=0.059	Diff. electrodes<br>16/128=0.125	Diff. electrodes<br>19/179=0.11
	Same electrode<br>4/20=0.20	Same electrode<br>4/32=0.125	Same electrode<br>8/52=0.15	Same electrode<br>7/34=0.21	Same electrode<br>13/48=0.27	Same electrode<br>20/82=0.24
	p_{fisher}=0.17	p_{fisher}=0.49	p_{fisher}=0.91	p_{fisher}=0.063	p_{fisher}=0.031	p_{fisher}=0.0037
