# Supplementary material for: Spatiotemporal Spike Coding of Behavioral Adaptation in the Dorsal Anterior Cingulate Cortex
Source: PLoS Biol. 2015 Aug 12;13(8):e1002222. doi: 10.1371/journal.pbio.1002222 (PMC4534466; doi:10.1371/journal.pbio.1002222)
Supplement: S1 Text — (PDF) [file pbio.1002222.s017.pdf]

Neuronal circuits can either detect coincident depolarizations due to spatiotemporally structured inputs, or loosely integrate all incoming inputs during a given task epoch up to threshold (see main text **Fig. 1c**, [1, 2]). Our analysis of dACC activity sought an unambiguous post-synaptic signaling of the task epoch during which dACC spike trains were emitted. This decoding approach is functionally relevant because different task epochs must result in different behavioral strategy adaptation to optimize performance.

When functioning in a coincidence detection mode, a post-synaptic neural decoder might discharge specifically to a given task epoch, if its input spike trains would have a spatiotemporal structure more different between task epochs than within this epoch. Alternatively, a downstream neural integrator might become selective for task epochs by receiving inputs from neurons that fire more in one task epoch (see main text **Fig. 1,2**).

The efficiency of a decoding strategy can be assessed by quantifying how dissimilar are spike trains within and between categories, in terms of either (spatio)temporal structure or spike count. Within the theoretical framework named spike train metrics, the distance or dissimilarity between two spike trains is measured as a function of both the importance of spike timing [3] and the spatial distinction between the activity from different input neurons [4].

1 Single-unit spike train metrics

The distance $d(s, s')$ between two spike trains $s,\ s'$ is defined as the minimal cost to transform s into s' [3]. This transformation consists in using one of the three following steps sequentially:

- adding a spike, for a cost of 1;
- deleting a spike, for a cost of 1;
- changing the time of a spike by an amount dt, for a cost $q \cdot dt$, where q is a free parameter that determines the importance of spike timing (also named timing sensitivity throughout the paper).

When $q = 0\ s^{-1}$, there is no cost for changing the timing. Consequently, the distance $d(s, s')$ corresponds to the absolute spike count difference between the two spike trains. As q increases, changing the timing of spikes becomes more and more costly. Thus, a small distance $d(s, s')$ implies that s and s' have spikes that match in time, i.e. the temporal structure must be conserved. Two spikes from s and s' may be moved to be matched if they are separated by at most $2/q$ second. Otherwise, it is less costly to delete the first spike and reintroduce a new matching spike, for a total cost of 2. Therefore, $2/q$ gives the maximal between-trial interspike interval for which timing is accounted for.

2 Multi-unit spike train metrics

A multi-unit spike train is defined as the pattern of discharges from different neurons observed in a given trial, each spike being labeled by the identity of the neuron that emitted it. To compute the distance $d(s, s')$ between two multi-unit spike trains $s,\ s'$, two parameters must be considered: the timing sensitivity q, and the degree of distinction k between spikes from different neurons. For example, if two neurons emit spike trains with statistically

identical temporal structures and fire with uncorrelated noise, then pooling their responses can be better for decoding. Conversely, if two neurons emit opposed signals (for instance an increase *vs.* a decrease of spiking in a given task epoch), then it is important to distinguish between them to maximize information. The distance $d(s, s')$ between two multi-unit spike trains is defined as the minimum cost to transform s into s', by using the steps previously described, with the additional possibility to change the identity of the neuron that fired a given spike, for a cost k. If $k = 0$, the identity of neurons does not matter at all. If $k \geq k_{max} = 2$, the responses are never matched between neurons, because removing a spike from a given neuron and replacing it by a spike from another neuron at the correct time is less costly. In general, two spikes from two different neurons may be matched if they are separated by less than $\frac{(2-k)}{q}$ second —so only very coincident spikes are matched for intermediate k values.

3 Classification

A leave-one-out process was used to classify a given spike train s into the task epoch E producing the most similar responses to s. The distance between s and the activity produced during E was defined as the median of the pairwise distances between s and any (other) spike train $s' \in E$. Therefore, one spike train s was predicted to belong to the task epoch E that minimized $median\,(d_{q,k}(s, s'))_{s' \in E,\, s' \neq s}$.

Note that we also ran a decoding analysis of dACC activity by using a small-distance biased classification algorithm orginally proposed by [3] ($z = -2$ in their eq. 5, i.e. the distance between s and the activity produced during E is $\left(\langle (d_q(s, s'))^{-2} \rangle_{s' \in E,\, s' \neq s}\right)^{\frac{1}{-2}}$). We did not show this method in the main body because *(i)* it hinders classification based on spike count decoding, and *(ii)* it leads to an overall decrease of the number of significant units and of the information (all analyzed single units, signed-rank test on $max_q(< I >_t)$, all $ps < 10^{-5}$). These effects are likely to be related to the frequent occurrence of zero pairwise distances in our dACC data set (due, for instance, to two empty spike trains or, for $q = 0$ s^{-1}, to two spike trains with the same spike count). Although the occurrence of zero pairwise distances was more frequent within task epochs, given the high variability of our data (main text **Fig. 5c**), it was also possible between task epochs. With the small-distance biased classification, the presence of at least one zero pairwise distance in both epochs triggered a chance-based clustering of spike trains, irrespective of the 0-distance frequency in the two task epochs. Despite the lower classification power of this method, it leads to identical conclusions regarding dACC coding properties as the method presented in main text (all analyses were checked with both methods; results for the single-units classification are shown in **S5 Fig.**). In general, for our very variable data (main text **Fig. 5c**), it is likely that any classification relying on outliers would be less efficient than a classification relying on a robust central value as the median.

A confusion matrix was built, in which the entry N_{ij} on line i and column j was the number of spike trains coming from task epoch i and predicted to belong to task epoch j. If a trial was equally distant to several epochs, the fraction $\frac{1}{N_{closest\ epochs}}$ was added to all these epochs. The information I_{raw} in the confusion matrix was:

$$I_{raw} = \frac{1}{N} \sum_{i,j} N_{ij} \cdot ln\Big(\frac{N_{ij} \cdot N}{\sum_k N_{ik} \cdot \sum_l N_{lj}}\Big) \quad (1)$$

with $N = \sum_{i,j} N_{ij}$. This corresponds to the mutual information between the actual classification of trials and the classification that one would get if the prediction were perfect. Hence, I_{raw} is always maximal for perfect

prediction, though the absolute maximum value depends on the balance of number of trials between the two task epochs. Therefore, we also computed a normalized information I_{norm} by dividing I_{raw} by its maximal (perfect prediction) value:

$$I_{norm} = \frac{I_{raw}}{-\frac{1}{N}\sum_i \left(\sum_j N_{ij}\right) \cdot ln\left(\frac{\sum_j N_{ij}}{N}\right)} \quad (2)$$

Note that the information has the advantage to intrinsically account for the distribution of the number of data points in different categories to be classified, which is not the case of some other measures of classification performance, such as percentage of correct [5]. This was important in our case because there were much less 1st reward or errors trials compared to repetition trials (**S1 Table**).

The information estimate is, in general, biased when only finite data is available. However, because the spike train metrics method makes the assumption that spike trains within one task-epoch appear more similar to one another than spike trains taken from two different task-epochs, it is globally less likely to generate the huge finite sample positive bias observed with the 'raw' binning method [6]. Because classical analytical formulae for bias estimation cannot be applied to the case of the confusion matrix [3], the bias was estimated empricially as the mean information computed in 1000 data sets created by randomly permuting the trials between task-epochs. This bias estimate was subtracted from the information estimate in the original data. In rare cases when slightly negative values were reached after bias-sustraction, the final information value was set to 0. Note that we verified that the q_{opt} found for the 1st reward vs. repetition classification was identical with or without bias correction, even though this classification had the smallest number of trials and could therefore be more sensitive to finite-sample effects. More generally, we assessed the possible remaining presence of a bias by computing for each neuron (or pair of neuron) the minimum trial number over task-epochs $N_{trial\ min}$. We then compared different statistics related to information (e.g. increase in information thanks to temporal sensitivity, gain in information during paired decoding, ...) between neurons (resp. pairs) with $N_{trial\ min}$ that was higher *vs.* lower than the median. While several factors may cause a difference between the group of high and low trial number (such as behavioral differences between sessions of different durations, ...), a finite-sample bias would be expected to have a very specific impact on the statisitical measurements. Indeed, a given effect may result from a bias if, consistenly in the two monkeys, the effect would decrease in the high trial number group and if this effect would be smallest in monkey M (which had the highest trial number, see **S1 Table**). This pattern was never observed, arguing that our results are very unlikely to reflect a finite-sample bias.

4 Interpretation of the classifier as a downstream decoding network and non-triviality of the timing-related information improvement

The classification algorithm described in the previous section can be related to the performance of different downstream neuronal circuits (**main text Fig. 1**). Indeed, the channels and membrane properties of single neurons can be approximately described by decaying filters (on the order of ms to hundreds of ms) of input spike trains [7]. In addition, the neuronal network's architecture can create decays on much longer timescales, or even quasi-perfect integration, which may implement short-term memory [8, 9].

When the downstream neuronal network acts as an integrator, it effectively 'sees' input spike trains through their

spike-count, and it would perform a classification tantamount to the metrics with $q = 0\ s^{-1}$.

For $q > 0s^{-1}$, the metrics is better interpreted through the equivalent similarity between spike trains. For any pair of spikes separated by an interval $\delta \geq 0$ and associated with a Victor and Purpura cost (or dissimilarity) $d(\delta)$, we can define the similarity $S = D_{max} - d(\delta)$. $D_{max} = 2$ is both the maximum dissimilarity between two spikes and the sum of the costs of removing a spike and of reinserting a new spike at the right time (see main text **Fig. 2a right**). Hence, for $\delta \leq \frac{2}{q}$, $S(\delta) = 2 - q\ \delta$, and else $S = 0$. This similarity can be related to the maximal depolarization reached through the summation of two excitatory post-synaptic potential (EPSPs) that would be caused by the two compared spikes. Indeed, if we take the (realistic) choice of an exponential synaptic trace $A\exp(-\frac{t}{\tau})$, we can notice that the maximal depolarization reached after summation of the two filtered synaptic traces is $A + A\exp(-\frac{\delta}{\tau})$. We can finally define an 'excess depolarization' E above a baseline (here, the depolarization reached with a single spike): $E(\delta) = A\exp(-\frac{\delta}{\tau})$. The functions $S(\delta)$ and $E(\delta)$ have similar shapes and may be matched; in particular, we can equate:

- the maximal amplitudes of S and E: $A = D_{max} = 2$
- the integrals of S and E: $\int f(\delta)d\delta = A\tau = \int S(\delta)d\delta = \frac{2}{q}$

In other words, the (synaptic) decaying time-scale τ can be matched to $\frac{1}{q}$ ([10]; see also [11, 12] for related ideas). For paired spikes, the more similar the two spikes are according to the Victor and Pupura distance, the more excited would a dowstream decoder (reacting with a time-scale $\approx \frac{1}{q}$) be through summation of the depolarizations induced by the two spike trains. Finally, when additional spikes are present in one spike train, each spurious spike induces an increase in the total dissimilarity equal to half the maximal dissimilarity that a spike pair can reach. Similarly, an isolated spike induces a spurious depolarization of maximal amplitude $\approx A$ once, while a maximally dissimilar spike pair reaches this depolarization twice (once for each spike of the pair).

The metrics therefore accounts for plausible constraints of the downstream circuits in terms of signal processing, assuming the presence of one main decaying timescale for input filtering. Analysis techniques explicitly using exponential filtering for spike train classification were indeed found to behave almost identically to the Victor and Purpura distance [11, 12, 13]. This is why the performance of the classification procedure is tantamount to the performance of these different decoding downstream circuits (rather than to the maximum amount of information that a perfect decoder, without any constraint, could reach).

Importantly, the presence of (task-epoch-specific) temporal structure does not necessarily cause an improvement of the decoding performance with a value $q_{opt} > 0$ compared to $q = 0$. Indeed, temporal modulations may co-vary with spike-count differences, implying a redundancy between the spike-count based and spike-timing-based information. Further, the temporal information accessible to a biologically plausible decoder might reveal less robust than a time-integrated spike count. This is particularly likely to happen in cases when the spike rate is consistently higher in one task-epoch compared to the other, leading to a between-task-epoch spike count difference that is consistent over time. This difference could be detected with more and more accuracy when evidence is accumulated over time through integration, leading to an efficient averaging-out of the noisy deviations over time. This configuration (firing rate consistently higher in one task-epoch) seems to qualitatively occur for dACC firing rates (main text **Fig. 1c, Fig. 3**). Along those lines, previous articles reported an absence of timing-sensitivity-

related information improvement even in the presence of category-specific temporal modulations in the spiking response [14, 13] (see in particular the fig. 2a in [13]). Hence, as pointed out in [13], the spike-train-based classification does not detect all the existing timescales of the analyzed neuronal activity. Instead, the spike-train-based classification aims at testing whether the reliability of temporal structure could allow a plausible downstream decoder to take advantage of it, which relates to the biological plausibility of temporal information transmission [15].

5 Algorithms and numerical methods

We ran all calculations on a cluster of 320 nodes (Consorzio Interuniversitario per le Applicazioni di Supercalcolo Per Università e Ricerca CASPUR), on a private cluster and on a PC laptop, using MATLAB (we adapted Victor's code, freely available at http://www-users.med.cornell.edu/~jdvicto/metricdf.html). For the single-unit decoding and response time analysis, we used Reich's c/MEX code and a modified MATLAB non-vectorized algorithm, respectively. For the multi-unit decoding analysis, we adapted Kreuz's vectorized algorithm in MATLAB code (to handle the case of empty spike trains). Unless mentioned otherwise, q was varied within $[0, 5, 10, 15, 20, 25, 30, 35, 40, 60, 80]s^{-1}$, whereas k was varied within [0,0.25,0.5,0.75,1,1.25,1.5,1.75,2].

References

[1] Rudolph M, Destexhe A. Tuning neocortical pyramidal neurons between integrators and coincidence detectors. J Comput Neurosci. 2003;14(3):239--51.

[2] Cain N, Shea-Brown E. Computational models of decision making: integration, stability, and noise. Curr Opin Neurobiol. 2012 Dec;22(6):1047--1053. Available from: `http://dx.doi.org/10.1016/j.conb.2012.04.013`.

[3] Victor JD, Purpura KP. Nature and precision of temporal coding in visual cortex: a metric-space analysis. J Neurophysiol. 1996;76(2):1310--26.

[4] Aronov D, Reich DS, Mechler F, Victor JD. Neural coding of spatial phase in V1 of the macaque monkey. J Neurophysiol. 2003;89(6):3304--27.

[5] Sindhwani V, Rakshit S, Deodhare D, Erdogmus D, Principe JC, Niyogi P. Feature selection in MLPs and SVMs based on maximum output information. 2004;15(4):937--948. Available from: `http://ieeexplore.ieee.org/stamp/stamp.jsp?arnumber=1310365`.

[6] Victor JD. Spike train metrics. Curr Opin Neurobiol. 2005 Oct;15(5):585--592. Available from: `http://dx.doi.org/10.1016/j.conb.2005.08.002`.

[7] Gerstner W, Kistler WM. Spiking Neuron Models. 2002;Available from: `http://dx.doi.org/10.1017/CBO9780511815706`.

[8] Seung HS, Lee DD, Reis BY, Tank DW. Stability of the memory of eye position in a recurrent network of conductance-based model neurons. Neuron. 2000 Apr;26(1):259--271.

[9] Lim S, Goldman MS. Balanced cortical microcircuitry for maintaining information in working memory. Nat Neurosci. 2013 Sep;16(9):1306--1314. Available from: http://dx.doi.org/10.1038/nn.3492.

[10] Victor JD, Purpura KP. Metric-space analysis of spike trains: theory, algorithms and application. Network: Computation in Neural Systems. 1997;8(2):127--164. Available from: http://informahealthcare.com/doi/abs/10.1088/0954-898X_8_2_003.

[11] van Rossum MC. A novel spike distance. Neural Comput. 2001 Apr;13(4):751--763.

[12] Paiva ARC, Park I, Príncipe JC. A comparison of binless spike train measures. Neural Computing and Applications. 2010 Apr;19(3):405–419. Available from: http://dx.doi.org/10.1007/s00521-009-0307-6.

[13] Chicharro D, Kreuz T, Andrzejak RG. What can spike train distances tell us about the neural code? J Neurosci Methods. 2011 Jul;199(1):146--165. Available from: http://dx.doi.org/10.1016/j.jneumeth.2011.05.002.

[14] Oram MW, Hatsopoulos NG, Richmond BJ, Donoghue JP. Excess synchrony in motor cortical neurons provides redundant direction information with that from coarse temporal measures. J Neurophysiol. 2001 Oct;86(4):1700--1716.

[15] London M, Roth A, Beeren L, Häusser M, Latham PE. Sensitivity to perturbations in vivo implies high noise and suggests rate coding in cortex. Nature. 2010 Jul;466(7302):123--127. Available from: http://dx.doi.org/10.1038/nature09086.
