# Supplementary material for: Spatiotemporal Spike Coding of Behavioral Adaptation in the Dorsal Anterior Cingulate Cortex
Source: PLoS Biol. 2015 Aug 12;13(8):e1002222. doi: 10.1371/journal.pbio.1002222 (PMC4534466; doi:10.1371/journal.pbio.1002222)
Supplement: S2 Text — (PDF) [file pbio.1002222.s018.pdf]

Spike-sorting relying on waveform shape (template) is reliable but does classify erroneously a small proportion of spikes. We explain below why the different types of possible artifacts are unlikely to have biased our results.

- First, coincident spikes from different neurons will create 'mixture waveforms' that will be rejected. Given that this phenomenon was very uncommon in our recordings, and given that the removal of synchronized spikes should decrease the reliability of both spike count and temporal coincidence decoding schemes, we don't expect this artifact to have a sizable impact on our analyses.

- Second, a small proportion of spikes accepted in a template are 'false positives' and belong to neurons different from the majoritary neuron. However, this is unlikely to favor spike-timing sensitive decoding over spike count decoding. Indeed, there was a bias toward having more cells firing prefentially during behavioral adaptation [1], therefore making it more likely that two randomly chosen neurons show the same firing preference over task-epochs.

  On one hand, this last and more common configuration would increase the reliability of both spike count and temporal patterns (in the latter case, provided that the included spike is not negatively interfering with the reliability of the temporal structure). Accordingly, when using spatially insensitive decoding, pairs of neurons with the same firing preference performed better compared to pairs with different spiking preferences (pairs with significant coding, rank-sum test comparing: *(i)* spatially insensitive spike-count-based decoding $\langle I\left(q=0,k=0\right)\rangle_t : p_s < 10^{-4}$; *(ii)* spatially insensitive decoding with spike-timing sensitivity $\langle I\left(q=10,k=0\right)\rangle_t : p_s < 10^{-2}$). This difference was not likely to reflect a different intrinsic property of the neurons between the pairs with same *vs.* different firing preference. Indeed, spatially separated decoding performed equivalently in the two groups ($\langle I\left(q=0,k=2\right)\rangle_t$ or $\langle I\left(q=10,k=2\right)\rangle_t$, all $p_s > 0.19$).

  On the other hand, in the less probable case when the 'noise spike' comes from a cell with a different spiking preference compared to the majoritary cell, both spike count and timing-sensitive decoding are likely to be negatively impacted, given the small probability that the noise spike can coincide with one precisely timed spike. Accordingly, when looking at pairs composed of two units with opposite firing preference, the information loss in spatially-insensitive ($k=0$) decoding compared to spatially-separated ($k=2$) decoding was not significantly distinct between timing sensitive and spike count codes (signed-rank test on $\left(\langle I\left(q=0,k=2\right)\rangle_t - \langle I\left(q=0,k=0\right)\rangle_t\right) - \left(\langle I\left(q=10,k=2\right)\rangle_t - \langle I\left(q=10,k=0\right)\rangle_t\right)$, all $ps > 0.1$). This suggests that optimal timing-sensitive (and spatially-insensitive) codes were not robustly better than spike-count at demixing two activities with opposite firing preference.

  Overall, it is very unlikely that the 'false positive' spikes in a template, that are in minority and which do not appear to robustly favor spike-timing sensitive decoding, could sizably participate in the effects of temporal structure we describe in the main text.

- Third, spikes of one neuron might pass from one template to another template (if the recording drifts), which could only potentially bias our pair of neurons analysis. The inter-electrode distance (150 μm of horizontal separation and, usually, different depths) made this phenomenon extremely unlikely between two different electrodes; this effect could only possibly affect pairs whose templates were sorted on the same electrode. Such `template exchange' could artificially produce low k_{opt} values in pairs recorded on the same electrode

as compared to pairs recorded from different electrodes, and artificially create the presence of pairs with $k_{opt} = 0$ (i.e. with the properties described in the main text, **Fig. 7**).

We tested this hypothesis by researching whether there was a consistent difference between pairs of neurons recorded from same *vs.* different electrodes. Note that such a difference may also arise if the inputs driving dACC are spatially segregated, making two closeby neurons more likely to receive similar inputs —as commonly observed, including in frontal areas [2]. In this case, the differences between pairs recorded on the same *vs.* different electrodes could be specific to, say, errors discrimination, because the inputs driving the neurons at different moments of the task may have different spatial organization. In contrast, a generalized and consistent difference between these two groups may reveal either a bias due to spike-sorting or a generalized spatial structure of inputs.

S3 Table describes the results of:

- a rank sum test comparing distributions of k_{opt} values,
- a Fisher test comparing the proportion of pairs with $k_{opt} = 0$

for significantly informative pairs recorded from the same *vs.* different electrodes.

For 1st reward discrimination, the distributions of k_{opt} values and the proportion of pairs with $k_{opt} = 0$ were statistically identical among the pairs recorded from the same or different electrodes. By contrast, for errors discrimination the k_{opt} values were higher (and the proportion of pairs with $k_{opt} = 0$ smaller) for the group of pairs recorded from different electrodes. This result appears consistent with the existence of a spatial organization of inputs driving discharges during errors, and inconsistent with a (general) influence of spike sorting artifacts.

References

[1] Quilodran R, Rothé M, Procyk E. Behavioral shifts and action valuation in the anterior cingulate cortex. Neuron. 2008 Jan;57(2):314--325. Available from: `http://dx.doi.org/10.1016/j.neuron.2007.11.031`.

[2] Schall JD, Morel A, King DJ, Bullier J. Topography of visual cortex connections with frontal eye field in macaque: convergence and segregation of processing streams. J Neurosci. 1995 Jun;15(6):4464--4487.
