# Supplementary material for: Spatiotemporal Spike Coding of Behavioral Adaptation in the Dorsal Anterior Cingulate Cortex
Source: PLoS Biol. 2015 Aug 12;13(8):e1002222. doi: 10.1371/journal.pbio.1002222 (PMC4534466; doi:10.1371/journal.pbio.1002222)
Supplement: S3 Text — (PDF) [file pbio.1002222.s019.pdf]

We verified that purely motor differences between 1st reward and repetition feedbacks were unlikely to produce the advantage of timing sensitivity for decoding. After target touch, arm-movements were largely a return from the target to the central 'lever' button occuring *after* gaze-shift. We therefore focused the analysis on eye movements, which were monitored with an infrared system (Iscan Inc., USA). We aimed at finding a threshold on the derivative of the recorded eye position which could define an eye movement. We filtered the signal with a gaussian of standard deviation 9 ms (changing this value by a few ms was not critical, see [1] for a similar approach). We then built a distribution of filtered eye-position derivatives, using peri-choice-saccade (0.1 s before to 0.5 s after targets onset) and post-reward (until +1 s) data, separately in X and Y. Distributions were gaussian-like supplemented with outliers (long tails). We used the threshold at which the data significantly differed from a gaussian — determined using the Grubbs Test implemented in the matlab file exchange function deleteoutliers [2] — to detect a movement in either X or Y. These X and Y thresholds matched well 'intuitive' saccade detection when we examined a large subset of traces. Note that we did not differentiate between saccades and blinks (which both result in large derivative values of the recorded eye position), because they can trigger spiking in the same area [3]. For simplicity, we use the expression 'eye movement' to refer to any threshold crossing for recorded eye speed.

We characterized the eye motor activity between the go signal for target touch (occuring after target fixation) and 1s post-reward. Monkey P was very often breaking fixation before reward time (not shown), while monkey M was often maintaining fixation after reward time (**S6 a Fig.**). In both monkeys, differences could be seen between 1st reward and repetition (e.g. in the number of saccades, latency of first saccade following the reward, see **S6 a,b Fig.** for monkey M). Note that while these differences accumulated earlier in monkey P in general, the advantage of temporal sensitivity for decoding appears rather slightly stronger in monkey M (**Fig. 4**). Note also that eye-movements may be correlated to attention and cognitive processing [4], a phenomenon which seemed to occur for late eye-shifts in monkey M. Indeed, trials with late post-first-reward 1st eye movement often led to a shorter response time of the monkey at the following trial (**S6 c Fig.**). Therefore, a correlation between these late saccades and neural activity would still be compatible with a cognitive correlate of the discharge.

We focused on monkey M which behavior allowed us to decode trials without any saccade or blink detected between the fixation period and the end of the analysis window (**S6 d,e,i Fig.**), or between the fixation period and 300 ms after the end of the analysis window (**S6 f,g,j Fig.**). This delay of 300 ms was chosen because it is likely to eliminate preparation activity directly triggering saccades (as the activity occuring, e.g., in the FEF [5]). We also excluded rare trials when, between saccade and reward time, the gaze had slowly drifted by more than one third of the inter-target difference. Because hand movements were almost always occuring after gaze shift, this process also minimized them. Beside, we stress that even though removing trials according to eye movements detection could induce some more pronounced differences in the proportion of the different targets between 1st reward and repetition, this was very unlikely to favor purely motor-based classification, as target reach probably happened too early (600 ms before the start of the analysis window) to still influence spiking.

Therefore, our trial-removal process would strongly reduce the advantage of temporal sensitivity for decoding if this advantage was reflecting motor-feedback activity (or premotor activity when the first movement occurs later than 300ms after the end of the analysis window). However, we found that removing putative motor-feedback or premotor activity until 600 ms post-feedback did not decrease the avantage of temporal sensitivity for decoding

(see **S6 Fig.** for full results). This strongly suggests that the advantage of temporal sensitivity for decoding was not linked to purely motor correlates in dACC spiking.

Note that eye-movement data were only available in 38 significant neurons among the 61 from monkey M in main text **Fig. 4a left**. Note also that the finite-sample bias should be similar between information values in eye-movement free data, and information values in data with identical number of trials (obtained through down-sampling, see **S6 d,e,f,g Fig.**). Therefore, this bias should not impact the comparison between these two types of information values (in **S6 e,g Fig.**). In addition, in order to consistently display bias-subtracted information in **S6 d,f Fig.** as in all figures of the manuscript, the finite-sample information bias was evaluated as the mean information in 1000 shuffle data sets for which eye-movement free trials were randomly permuted between task-epochs (Materials and Methods).

References

[1] Martinez-Conde S, Macknik SL, Hubel DH. Microsaccadic eye movements and firing of single cells in the striate cortex of macaque monkeys. Nat Neurosci. 2000 Mar;3(3):251--258. Available from: http://dx.doi.org/10.1038/72961.

[2] Shoelson B. Deleteoutliers; 2003. Available from: http://www.mathworks.fr/matlabcentral/fileexchange/3961-deleteoutliers.

[3] Bodis-Wollner I, Bucher SF, Seelos KC. Cortical activation patterns during voluntary blinks and voluntary saccades. Neurology. 1999 Nov;53(8):1800--1805.

[4] Katnani HA, Gandhi NJ. Time course of motor preparation during visual search with flexible stimulus-response association. J Neurosci. 2013 Jun;33(24):10057--10065. Available from: http://dx.doi.org/10.1523/JNEUROSCI.0850-13.2013.

[5] Hanes DP, Thompson KG, Schall JD. Relationship of presaccadic activity in frontal eye field and supplementary eye field to saccade initiation in macaque: Poisson spike train analysis. Exp Brain Res. 1995;103(1):85--96.
