# Supplementary material for: Spatiotemporal Spike Coding of Behavioral Adaptation in the Dorsal Anterior Cingulate Cortex
Source: PLoS Biol. 2015 Aug 12;13(8):e1002222. doi: 10.1371/journal.pbio.1002222 (PMC4534466; doi:10.1371/journal.pbio.1002222)
Supplement: S4 Text — (PDF) [file pbio.1002222.s020.pdf]

For the behavioral response time analysis, we wanted to quantify to which extent a spike train s was an outlier within the entire set of spike trains produced at 1st reward, i.e. how much it deviated from the discharge `typically' emitted during that epoch (for any given neuron).

1 Principle of the computation of the deviation from a prototypical spike train

In order to evaluate how much a spike train s deviates from usual 1st reward activity, we thought of taking the median of all pairwise dissimilarities between this spike train s and any other spike train s' emitted during the 1st reward epoch. We then wondered which dissimilarity measure was appropriate.

The original Victor & Purpura distance $d(s, s')$ soon appeared problematic. Indeed, it sums the costs to match any spike of train s to a spike of train s' (**S1 Text**, [1]). Thus, all pairwise distances involving a train with many spikes tend to be larger than those involving a train with little spikes. For instance, let $s = \{0.1, 0.5\}$ (i.e. it contains one spike at time $t = 0.1$ s and a second spike at $t = 0.5$ s) and $s' = \{0.11, 0.51\}$. Their distance is then $d_1(s, s') = 2 \cdot 0.01\, q$ (for an appropriate $q \leq 200$). Now, if $s = \{0.1\}$ and $s' = \{0.11, 0.51\}$, then $d_2(s, s') = 1 + 0.01\, q$. Therefore, if we take q to roughly match the temporal jitter of ± 0.01 s (i.e. $q = 100\ s^{-1}$), then $d_1 = d_2$, though during the first distance computation the spike matching was as temporally precise as, and more reliable than, during the second distance computation. In order to avoid this scaling with spike number, we divided the Victor & Purpura distance by the number of times when two spikes (from the two trials) were 'coincident' (i.e., 'matched' during dissimilarity computation). Two spikes were considered 'coincident' when they were associated with a distance $d < D_{max} = 2$. There was no coincidence both in cases when a spike was deleted and then reinserted at the right time (for $q > 0$), and in cases when a spike was simply removed or added. Note that for $q = 0$, the number of 'coincidences' (i.e., 'spike matchings') is the spike count of the trial with fewer spikes. The normalized distance was taken as:

$$d^*(s, s') = \frac{q}{N_c} \cdot \sum_{i}^{N_c} \left| t_s^i - t_{s'}^i \right| + \frac{C}{N_c} = q \cdot \langle dt \rangle + \frac{C}{N_c} \quad (1)$$

where N_c denotes the number of coincident spike pairs, t_s^i the time of the ith coincident spike in train s, $\langle dt \rangle$ the mean jitter among coincident spikes, and C the total cost for inserting and/or deleting spikes. The first term quantifies the dissimilarity due to coincident (i.e., 'matched') spikes, whereas the second term is the dissimilarity due to unmatched spikes. For $q > 0$, this measure quantifies the reliability of temporal coincidence detection between two spike trains. For $q = 0$ s^{-1}, it quantifies the absolute spike count difference relative to the shared spike count. In both cases, the normalized distance behaves similarly to an inverted signal-to-noise ratio. In this interpretation, the signal is taken as the coincident spikes. The noise is the unmatched spikes, and the temporal jitter of coincident spikes relative to the considered 'coincidence window' for $q > 0$.

In the absence of coincident spikes, we simply used the original Victor & Purpura distance. For $q = 0$ s^{-1}, the absence of coincident spikes only happens when one spike train is empty. In this case, some intuitive order relations are conserved. Let s_x denote a spike train containing x spikes. Then: $d(s_0, s_x) > d(s_0, s_y)$ when $x > y$, and $d(s_0, s_x) > d(s_1, s_x)$ when $x > 1$. For $q > 0$, the absence of matching spike could also happen when the distance between two spike trains s_x, s_y is maximum and equal to $x + y$, because no spikes are close enough in time to be

advantageously matched. In this case, the distance grows with the number of spikes that are unmatchable, i.e. very dissimilar, which appears sound. Recall that, because our results showed an increase of information driven by temporal spike matching, this 'no coincident spikes' situation was likely to be unfrequent.

Note that the new distance we designed has a different purpose and effect from the previously proposed division by the sum of spike count in the two spike trains and, more generally, from other re-scaled spike train dissimilarity measures [2]. Indeed, rather than bounding the measure, or merely averaging some jitter statistics, we tried to build a measure that would evaluate dissimilarities between spike trains as perceived by different plausible decoders which are more or less sensitive to spike timing and spike count, without being biased by the number of spikes. Notably, we did not want the spikes that could not be matched to enter in the normalization factor for the dissimilarity measure (which would happen with a simple division by spike count).

2 Classification with the new normalized metrics d^*

We expected the normalized distance $d^*(s, s')$ to show similar classification ability as compared to the classical Victor & Purpura distance d. Indeed, for any spike train s, since both the intra- and inter-task epoch distances d to s will increase with the spike count of s, a smaller d for a given task epoch still indicates a greater similarity relative to the other task epoch(s). To corroborate this hypothesis, we tested the 1st reward classification with the normalized metrics. To do so, we used the very same trials that have been extracted for the response time analysis. Both the number and the identity of the significant neurons were consistent with those found with the classical metrics (Monkey M: 65 significant neurons vs. 61, of which 57 are shared; monkey P: 50 significant neurons in both cases, 44 shared). The classification results were also equivalent, as confirmed by a rank sum test comparing the maximum (over timing sensitivity values) time-averaged information among significant neurons (all $p_s > 0.74$). In addition, the normalized metrics uncovered an increase of time-averaged information with timing sensitivity adaptation, independently in both monkeys (Friedman ANOVA on time-averaged information $< I >_t$, all $p < 10^{-8}$; $q_{opt} = 15s^{-1}$ and $10s^{-1}$ for monkey M and P respectively showed higher rank than $q = 0$ after post-hoc comparisons with Tukey's honestly significant criterion).

3 During response-time prediction, even in cases when the prediction power of $q = 0$ and q_{opt} was equivalent, these performances appeared to rely on different mechanisms

It is interesting to note that the timing-sensitive measure $d^*(q_{opt})$ is also influenced by spike count. Hence, one might wonder whether the prediction power of $d^*(q_{opt})$ could come exclusively from spike count, while spike-timing differences would act as a negligible noise. Our data actually strongly argue against this possibility and are consistent with a significant effect of spike-timing. Indeed:

1. Using $d^*(q_{opt} \approx 10s^{-1})$ allowed to significantly improve behavioral prediction in monkey M (main text fig. 8), and to significantly improve 1st reward vs. repetition classification compared to $d^*(q = 0)$ in both monkeys (signed-rank test among neurons with significant classification abilities, comparing $< I(q_{opt}) >_t$ and $< I(q = 0) >_t$, monkey M, $p = 2.9\ 10^{-4}$; monkey P, $p = 1.4\ 10^{-3}$). Hence, using $q_{opt} \approx 10s^{-1}$ on

the typical interspike intervals present in our spike trains indeed led to substantial changes of d^* compared to $q = 0$.

2. Concerning the behavioral prediction in monkey P, we can also show that even though $d^*(q_{opt})$ and $d^*(q = 0)$ both led to similar performance, they probably did it for different reasons (i.e. they probably relied on different neurons and different analysis windows). Indeed, if temporal sensitivity had a negligible impact on the $\bar{D}$ measure (Materials and Methods), then the difference $Diff_{\bar{D}} = \bar{D}(q_{opt}) - \bar{D}(q = 0/s)$ should be negligible noise. Under this hypothesis, adding a surrogate noise $Diff_{\bar{D}}^{surr}$ (with statistics similar to $Diff_{\bar{D}}$) to $\bar{D}(q = 0/s)$ should lead to a surrogate of $\bar{D}(q_{opt})$: $Diff_{\bar{D}}^{surr} + \bar{D}(q = 0/s) = \bar{D}(q_{opt})^{surr}$. This $\bar{D}(q_{opt})^{surr}$ should then have a similar bias score b (see Materials and Methods) to the bias score of the original $\bar{D}(q_{opt})$. We tested this hypothesis, creating 1000 surrogates $Diff_{\bar{D}}^{surr}$ from $Diff_{\bar{D}}$ by randomly shuffling the values of $Diff_{\bar{D}}$ between neurons (identical conclusions were also reached when shuffling between analysis windows or between both neurons and analysis windows). Importantly, only 2 % of these surrogates had bias scores b superior or equal to the one of the original $\bar{D}(q_{opt})$ (using analysis windows ending between 0.1 and 1s by steps of 0.1 s for bias score computation, as in main text). In other words, the null hypothesis (stating that temporal sensitivity at $q_{opt} = 10/s$ was only producing spurious negligible changes in d^* relative to $q = 0$) could be rejected with a p-value of 0.02. Similar results were reached when computing the bias score using only analysis windows during which $q = 0$ was leading to a substantial value of $\bar{D}$ (analysis windows between 250 and 450 ms, increasing in steps of 50 ms, p=0.011). Hence, the bias scores of $q_{opt} = 10/s$ and $q = 0$ appeared to reach similar values by relying on different neurons and analysis windows. This suggests that in monkey P, there is a subset of neurons for which the deviation from the prototypical spike count better predicts behavior, and there are other neurons for which the deviation from the prototypical temporal pattern better predicts behavior.

These results further emphasize that, in both monkeys, the significant bias score obtained when using $q_{opt} = 10/s$ indeed suggest the relevance of dACC post-first-reward spike timing during downstream stages of processing.

References

[1] Victor JD, Purpura KP. Nature and precision of temporal coding in visual cortex: a metric-space analysis. J Neurophysiol. 1996;76(2):1310--26.

[2] Naud R, Gerhard F, Mensi S, Gerstner W. Improved similarity measures for small sets of spike trains. Neural Comput. 2011 Dec;23(12):3016--3069. Available from: http://dx.doi.org/10.1162/NECO_a_00208.
